# Burden of diabetes and hyperglycaemia in adults in the Americas, 1990–2019: a systematic analysis for the Global Burden of Disease Study 2019

**DOI:** 10.1016/S2213-8587(22)00186-3

**Published:** 2022-09

**Authors:** GBD 2019 Diabetes in the Americas Collaborators, Ewerton Cousin, Maria Inês Schmidt, Kanyin Liane Ong, Rafael Lozano, Ashkan Afshin, Abdelrahman I Abushouk, Gina Agarwal, Marcela Agudelo-Botero, Ziyad Al-Aly, Jacqueline Elizabeth Alcalde-Rabanal, Nelson Alvis-Guzman, Nelson J Alvis-Zakzuk, Benny Antony, Malke Asaad, Till Winfried Bärnighausen, Sanjay Basu, Isabela M Bensenor, Zahid A Butt, Ismael R Campos-Nonato, Vijay Kumar Chattu, Michael H Criqui, Parnaz Daneshpajouhnejad, Claudio Alberto Dávila-Cervantes, Edgar Denova-Gutiérrez, Samath Dhamminda Dharmaratne, Daniel Diaz, Irina Filip, Mohamed M Gad, MA Garcia-Gordillo, Shakiba Ghasemi Assl, Sameer Vali Gopalani, Rafael Alves Guimarães, Rajat Das Gupta, Nima Hafezi-Nejad, Maryam Hashemian, Simon I Hay, Tanvir Kahlon, Jagdish Khubchandani, Ruth W Kimokoti, Adnan Kisa, Barthelemy Kuate Defo, Iván Landires, Ted R Miller, Ali H Mokdad, Linda Morales, Shane Douglas Morrison, Yeshambel T Nigatu, Virginia Nuñez-Samudio, Andrew T Olagunju, Seithikurippu R Pandi-Perumal, Urvish K Patel, Amir Radfar, Maria Rios-Blancas, Leonardo Roever, Seyedmohammad Saadatagah, Juan Sanabria, Itamar S Santos, Thirunavukkarasu Sathish, Mahsima Shabani, Omid Shafaat, Sara Sheikhbahaei, Diego Augusto Santos Silva, Ambrish Singh, Jasvinder A Singh, Marcos Roberto Tovani-Palone, Diana Zuleika Velazquez, Siddhesh Zadey, Mohsen Naghavi, Theo Vos, Bruce B Duncan

## Abstract

**Background:**

High prevalence of diabetes has been reported in the Americas, but no comprehensive analysis of diabetes burden and related factors for the region is available. We aimed to describe the burden of type 1 and type 2 diabetes and that of hyperglycaemia in the Americas from 1990 to 2019.

**Methods:**

We used estimates from GBD 2019 to evaluate the burden of diabetes in adults aged 20 years or older and high fasting plasma glucose in adults aged 25 years or older in the 39 countries and territories of the six regions in the Americas from 1990 to 2019. The main source to estimate the mortality attributable to diabetes and to chronic kidney disease due to diabetes was vital registration. Mortality due to overall diabetes (ie, diabetes and diabetes due to chronic kidney disease) was estimated using the Cause of Death Ensemble model. Years of life lost (YLLs) were calculated as the number of deaths multiplied by standard life expectancy at the age that the death occurred, years lived with disability (YLDs) were estimated based on the prevalence and severity of complications of diabetes. Disability-adjusted life-years (DALYs) were estimated as a sum of YLDs and YLLs. We assessed the association of diabetes burden with the level of development of a country (according to the Socio-demographic Index), health-care access and quality (estimated with the Healthcare Access and Quality Index), and diabetes prevalence. We also calculated the population attributable fraction (PAF) of diabetes burden due to each of its risk factors. We report the 95% uncertainty intervals for all estimates.

**Findings:**

In 2019, an estimated total of 409 000 (95% uncertainty interval 373 000–443 000) adults aged 20 years or older in the Americas died from diabetes, which represented 5·9% of all deaths. Diabetes was responsible for 2266 (1930–2649) crude DALYs per 100 000 adults in the Americas, and high fasting plasma glucose for 4401 DALYs (3685–5265) per 100 000 adults, with large variation across regions. DALYs were mostly due to type 2 diabetes and distribution was heterogeneous, being highest in central Latin America and the Caribbean and lowest in high-income North America and southern Latin America. Between 1990 and 2019, age-standardised DALYs due to type 2 diabetes increased 27·4% (22·0–32·5). This increase was particularly high in Andean Latin America and high-income North America. Burden for both type 1 and type 2 diabetes across countries increased with higher diabetes prevalence and decreased with greater Socio-demographic and Healthcare Access and Quality Indices. Main risk factors for the burden were high BMI, with a PAF of 63·2% and dietary risks, with a PAF of 27·5%. The fraction of burden due to disability has increased since 1990 and now represents nearly half of the overall burden in 2019.

**Interpretation:**

The burden of diabetes in the Americas is large, increasing, heterogeneous, and expanding. To confront the rising burden, population-based interventions aimed to reduce type 2 diabetes risk and strengthening health systems to provide effective and cost-efficient care for those affected are mandatory.

**Funding:**

Bill & Melinda Gates Foundation.

## Introduction

The challenge of the non-communicable disease (NCD) burden has led the UN and WHO to prioritise NCD control with a focus on five disease groups: cardiovascular diseases, cancer, chronic obstructive pulmonary disease, diabetes, and mental health.^1^ Of these diseases, only diabetes has shown a continuing major increase in burden, assessed by disability-adjusted life-years (DALYs) from 1990 to 2019.^2^

Studies looking specifically at global diabetes burden, considering both mortality and disability, are sparse. A Global Burden of Diseases, Injuries, and Risk Factors Study (GBD) 2015 analysis in the Eastern Mediterranean region showed a major and heterogeneous burden of diabetes and chronic kidney disease.^3^ However, few evaluations are available for other regions of the world.^4^ In addition, to our knowledge, no such study has been done separately by type of diabetes, which is feasible with the GBD 2019 cycle.

Diabetes in the Americas, composed of six GBD regions—high-income North America, the Caribbean, and central, Andean, tropical, and southern Latin Americas—which have great cultural and socioeconomic heterogeneity, is an important and growing problem.^5^ Major overall diabetes burden has been reported for some of its countries.^6^, ^7^ This finding is particularly relevant considering the heterogeneous rates of diabetes reported for the Americas, with very high prevalence in the English-speaking Caribbean.^8^ A broader analysis of the diabetes burden throughout the Americas could highlight gaps and challenges for specific subregions and identify areas that are in greater need of intervention.

**Research in context**

**Evidence before this study**

We searched PubMed on March 1, 2021, using the terms [diabetes mellitus AND “disease burden” AND America], without restrictions for language or article type. We also searched the Pan American Health Organization website for relevant publications on diabetes in the region on Aug 2, 2021. Although we found data regarding prevalence, incidence, and mortality, little data on burden of diabetes was available for the Americas, and we found no data addressing separately the burden of type 1 and type 2 diabetes.

**Added value of this study**

Using standard estimates from the Global Burden of Diseases, Injuries, and Risk Factors Study (GBD) 2019, we document a large burden of diabetes and hyperglycaemia in the Americas, mostly due to type 2 diabetes, with ample heterogeneity across its regions. A cluster of countries within central Latin America and the Caribbean presented a remarkably high burden in 2019. The main driver of the increase in burden was the increasing prevalence of diabetes. Greater development and, most notably, greater health-care access and quality mitigate part of the burden, as illustrated by high-income North America, which presents a large diabetes prevalence but only a mid-level diabetes burden. From 1990 to 2019, the age-standardised burden of diabetes increased in all regions for both type 1 and type 2 diabetes, and the burden of type 2 diabetes was particularly increased in high-income North America and Andean Latin America.

**Implications of all the available evidence**

The burden of diabetes continues to increase and expand throughout the Americas. Unless strong actions are taken, this burden is likely to increase further in the foreseeable future, given the ever-growing prevalence of diabetes. Although better access to high-quality health care is much needed in the region, vigorous implementation of public health policies aimed at primary prevention of type 2 diabetes is of essence.

In this study our aim was to describe the burden of type 1 and type 2 diabetes and hyperglycaemia across the Americas, and to evaluate trends and causes of this burden from 1990 to 2019. This manuscript was produced as part of the GBD Collaborator Network and in accordance with the GBD Protocol.

## Methods

### Overview

We analysed the burden of diabetes in adults aged 20 years or older in 39 countries and territories, hereafter called countries, across the six regions (appendix p 74) of the Americas from 1990 to 2019 using estimates from GBD 2019. To measure the disease burden, we used DALYs, calculated as the sum of premature mortality (years of life lost [YLLs]) and disability (years lived with disability [YLDs]). We also reported deaths, prevalence, and incidence. We briefly describe these estimations here, but they are more extensively described in the appendix (pp 20, 38) and elsewhere.^2^, ^9^, ^10^

GBD characterises the burden of diabetes through two distinct approaches (appendix p 9). The first, which considers diabetes as a disease, estimates the burden directly due to diabetes and due to chronic kidney disease resulting from diabetes. For this report, we joined these two, considering their sum as the burden due to diabetes. The second, expressed by the risk factor high fasting plasma glucose, joins the burden due to diabetes and chronic kidney disease resulting from diabetes with that caused indirectly by diabetes (and by a lesser degree of hyperglycaemia) through other diseases (cardiovascular diseases, neoplasms, dementia, vision impairment related to cataract and glaucoma, and tuberculosis).

### Burden of diabetes and of chronic kidney disease due to diabetes

To estimate all-cause mortality, GBD gathers data from vital registration, sample registration, and censuses and surveys. Annual vital registration data were available for most countries (see the appendix p 18 for more detail). To estimate the mortality attributable to diabetes and to chronic kidney disease due to diabetes, the fundamental resource is vital registration, based on the International Classification of Diseases (ICD), versions 9 (codes 250 and 755.1) and 10 (E10–E14 and P70.2). Adjustments for under-reporting of deaths are done before modelling. Ill-defined causes of deaths (eg, sepsis) and deaths due to improbable or poorly described causes, the latter including for IDC-10 code E14 (unspecified diabetes mellitus), are redistributed before modelling using previously defined algorithms (appendix p 14), with resultant fractions of these deaths being added to deaths attributed to type 1 or type 2 diabetes or chronic kidney disease due to either type of diabetes. GBD then estimates cause-specific mortality rates separately by type of diabetes using the Cause of Death Ensemble model. The Cause of Death Ensemble model combines results from different statistical models, weighing each model based on its out-of-sample predictive validity. After this process, cause of death correction (CoDCorrect) makes the estimates for all causes of death collectively exhaustive and mutually exclusive. During this step, GBD also calculates YLLs as the number of deaths multiplied by standard life expectancy at the age that death occurred.^10^

To estimate the non-fatal burden expressed through YLDs, GBD gathers data on diabetes and the likelihood of complications (ie, sequelae) and performs the following steps. First, prevalence of diabetes was obtained from population-based surveys, with diabetes being defined by the reference case definition of fasting plasma glucose of more than 126 mg/dL (7 mmol/L) or on treatment. When studies used other criteria—eg, based on glycated haemoglobin or the oral glucose tolerance test—their results were adjusted to this reference (appendix p 45). GBD then applies DisMod-MR 2.1, a Bayesian meta-regression tool, to produce estimates of diabetes prevalence and incidence. To separate the prevalence due to diabetes by type, the prevalence due to type 1 diabetes is calculated using all available data related to population-representative estimates. The prevalence of type 2 diabetes is then calculated by subtracting the estimated type 1 diabetes prevalence from the overall diabetes prevalence. Second, the likelihood of sequelae is estimated from a relevant literature review. Prevalence of specific diabetes sequelae (eg, amputation) are calculated in age and sex strata by multiplying the prevalence of diabetes times the likelihood of the sequela. Third, YLDs for a sequela are calculated as the prevalence of the sequela multiplied by the level of disability it causes. Level of disability (as assessed by disability weights; appendix p 49) is estimated based on surveys done in several countries worldwide, including Peru and USA from the Americas.^2^ YLDs are then summed across age and sex strata and sequelae to produce total YLDs due to diabetes. The derived YLDs can be conceived of as health loss from living in a less than optimum health state due to diabetes and its complications. The appendix (p 75) provides the number of the studies used for these non-fatal estimations. Studies specific to type 1 diabetes were less frequent, particularly for Andean and central Latin America and for the Caribbean. Finally, YLDs are summed with YLLs to produce burden in DALYs for overall and for each type of diabetes.

### Burden of high fasting plasma glucose

To estimate this wider burden when diabetes is also considered as a risk factor for various other diseases, GBD uses the construct of high fasting plasma glucose (available only for adults aged >25 years) as a risk factor. To calculate the burden due to high fasting plasma glucose for causes for which meta-analyses of the literature describe increasingly greater risk at progressively higher glycaemic levels (ischaemic heart disease, ischaemic stroke, subarachnoid haemorrhage, intracerebral haemorrhage, and peripheral vascular disease), GBD estimates each population's fasting plasma glucose distribution from studies presenting individual glucose data or population mean and standard deviation of fasting plasma glucose. When these values are not available, the distribution is predicted from diabetes prevalence. The population attributable fraction (PAF) for these outcomes is then estimated by contrasting risk at this distribution against that expected at the theoretical minimum-risk exposure level, based on a continuous risk–outcome curve derived using non-parametric Bayesian spline methods.^11^ The theoretical minimum-risk exposure level is defined as the level of fasting plasma glucose that minimises risk at the population level. For causes for which the relative risk has been characterised principally in meta-analyses comparing risk in the presence or absence of diabetes (pancreatic, ovarian, colorectal, bladder, lung, breast, and liver cancer; tuberculosis; glaucoma; cataracts; and dementia), the PAF is calculated based on that relative risk. The attributable burden for all relevant outcomes (as mentioned previously), calculated using their respective PAFs, is summed with that directly related to diabetes to produce the overall burden due to high fasting plasma glucose, expressed here in crude DALYs. The appendix (p 75) provides the number of studies used to obtain these estimations.

### Other definitions and analytical approaches

GBD gathers data on behavioural, environmental and occupational, and metabolic risk factors for diabetes, as previously described.^9^ The prevalence of continuously expressed risk factors such as high BMI are estimated by their summary exposure value. The summary exposure value weighs the prevalence of different levels of the risk factor by the severity of the burden caused at each level. The PAF of diabetes burden due to each of its risk factors is the sum of the excess burden from premature death and disability due to the specific risk factor divided by the total diabetes burden.

GBD expresses the level of development of a country by the Socio-demographic Index (SDI), which is a composite measure of income per capita, total fertility rate (age <25 years), and average educational attainment (for those aged ≥15 years).^9^ GBD uses the Healthcare Access and Quality (HAQ) Index to estimate personal health-care access and quality in each country based on risk-standardised mortality estimates from causes that should not result in death in the presence of high-quality health care.^12^

We report the 95% uncertainty intervals (UIs) obtained by running 1000 draws of each step of the estimation processes described previously, propagating these values through the overall analysis, and taking the final 2·5th and 97·5th percentile values as UI limits.

GBD 2019 analyses were done with Python (version 3.6.2), Stata (version 13), and R (version 3.5.0). The analyses specific to this study were done with R (version 3.6).

### Role of the funding source

The funder of this study had no role in study design, data collection, data analysis, data interpretation, or the writing of the report. The corresponding author had full access to the data in the study and final responsibility for the decision to submit for publication.

## Results

### Burden of type 1 and type 2 diabetes

In 2019, an estimated total of 409 000 (95% UI 373 000–443 000) adults aged 20 years or older in the Americas died from diabetes of both types, which represented 5·9% of all deaths, and age-standardised DALYs had increased 26·2% (20·8–31·3) from 1990 (appendix pp 77–79). Additionally, diabetes of both types was responsible for 2266 (1930–2649) crude DALYs per 100 000 adults in the Americas. For type 2 diabetes, the age-standardised death rate in the Americas in 2019 was 46·8 (42·8–50·5) per 100 000 adults, which was 25% higher than the global age-standardised death rate of 37·4 (34·3–40·3) per 100 000 adults (table). The pattern was heterogeneous across regions: central Latin America and the Caribbean had the highest age-standardised death rates, and high-income North America had the lowest. From 1990 to 2019, death rates increased by 19·0% (12·3–26·2) in the Americas, in contrast to a global average increase of 13·5% (6·9–20·5; table). This increase was highest in Andean Latin America (30·9% [8·5–55·5]; table), but in other regions, such as tropical Latin America, the death rate had decreased by as much as 10·1% (6·1–14·5).

**Table:** Age-standardised rates of deaths and DALYs due to type 1 and type 2 diabetes in adults (20 years or older) in the Americas in 1990 and 2019, and percentage changes from 1990 to 2019

		**Age-standardised death rate per 100 000**			**Age-standardised DALY rate per 100 000**		
		1990	2019	Percentage change, 1990–2019	1990	2019	Percentage change, 1990–2019
**Type 1 diabetes**							
Global		3·2 (2·6 to 3·9)	2·9 (2·4 to 3·6)	−9·3% (−19·8 to 3·1)	139·0 (114·8 to 164·0)	134·5 (110·6 to 159·7)	−3·2% (−12·3 to 6·7)
The Americas		2·9 (2·4 to 3·5)	3·3 (2·6 to 4·2)	13·5% (0·7 to 27·1)	141·9 (119·5 to 166·9)	161·1 (130·9 to 191·6)	13·5% (2·5 to 23·1)
	High-income North America	2·0 (1·7 to 2·5)	2·2 (1·7 to 2·6)	11·6% (−6·6 to 22·8)	135·1 (111·1 to 163·4)	151·4 (122·0 to 185·6)	12·1% (1·4 to 18·5)
	Central Latin America	4·0 (3·2 to 5·0)	5·8 (4·1 to 8·0)	45·3% (19·3 to 73·0)	147·8 (120·8 to 181·1)	220·5 (164·5 to 289·0)	49·1% (26·2 to 73·0)
	Caribbean	5·9 (4·6 to 7·0)	5·5 (4·2 to 7·0)	−7·6% (−25·8 to 13·4)	218·4 (173·7 to 259·4)	216·4 (170·3 to 271·4)	−0·9% (−17·9 to 19·1)
	Andean Latin America	3·2 (2·5 to 4·1)	3·4 (2·4 to 4·9)	5·6% (−17·5 to 31·0)	123·3 (99·0 to 153·4)	130·6 (96·6 to 171·6)	5·9% (−13·6 to 28·3)
	Tropical Latin America	3·9 (3·1 to 4·8)	2·8 (2·2 to 3·6)	−27·8% (−35·6 to −20·5)	150·9 (124·9 to 182·7)	132·8 (107·1 to 157·8)	−12·0% (−22·1 to −4·2)
	Southern Latin America	3·4 (2·5 to 4·2)	2·7 (2·0 to 3·5)	−21·3% (−29·9 to −9·2)	132·1 (107·1 to 156·8)	119·8 (95·9 to 145·4)	−9·3% (−16·6 to −1·4)
**Type 2 diabetes**							
Global		32·9 (30·8 to 35·1)	37·4 (34·3 to 40·3)	13·5% (6·9 to 20·5)	1151·8 (1001·7 to 1326·4)	1454·5 (1235·1 to 1714·4)	26·3% (20·2 to 31·8)
The Americas		39·3 (36·9 to 41·0)	46·8 (42·8 to 50·5)	19·0% (12·3 to 26·2)	1471·5 (1281·0 to 1684·5)	1874·6 (1589·6 to 2210·7)	27·4% (22·0 to 32·5)
	High-income North America	23·9 (22·2 to 25·3)	27·5 (24·9 to 29·6)	14·7% (9·6 to 20·5)	1018·3 (861·9 to 1204·6)	1361·8 (1111·8 to 1658·2)	33·7% (27·8 to 39·4)
	Central Latin America	79·8 (75·3 to 83·0)	91·6 (80·4 to 103·4)	14·8% (1·9 to 28·1)	2747·0 (2419·8 to 3105·2)	3264·0 (2799·3 to 3829·4)	18·8% (10·1 to 27·8)
	Caribbean	72·6 (67·7 to 77·4)	66·5 (56·0 to 78·6)	−8·4% (−22·0 to 7·2)	2411·6 (2113·6 to 2760·7)	2597·8 (2161·5 to 3116·6)	7·7% (−3·8 to 19·2)
	Andean Latin America	41·0 (37·0 to 45·9)	53·6 (44·6 to 64·0)	30·9% (8·5 to 55·5)	1252·2 (1099·7 to 1437·6)	1694·3 (1420·8 to 2009·6)	35·3% (18·3 to 52·6)
	Tropical Latin America	57·6 (53·8 to 60·3)	51·8 (46·8 to 55·0)	−10·1% (−14·5 to −6·1)	1876·1 (1657·0 to 2117·8)	1780·8 (1531·7 to 2057·4)	−5·1% (−9·1 to −1·4)
	Southern Latin America	40·2 (37·5 to 42·7)	39·2 (35·5 to 42·7)	−2·6% (−7·6 to 2·6)	1155·5 (1023·0 to 1309·7)	1368·8 (1149·7 to 1629·4)	18·5% (10·6 to 26·1)

Data in parentheses are 95% uncertainty intervals. DALYs=disability-adjusted life-years.

Type 2 diabetes caused 1874·6 (95% UI 1589·6–2210·7) age-standardised DALYs per 100 000 adults in 2019 in the Americas, which is 29% higher than the burden seen globally (1454·5 [1235·1–1714·4]; table). From 1990 to 2019, age-standardised DALYs in type 2 diabetes increased by 27·4% (22·0–32·5; table) in the Americas. Crude DALYs, which are not adjusted for population ageing, rose 59·2% (52·8–65·5; appendix p 80). In 2019, regions with the highest mortality (Central Latin America and the Caribbean) also presented the greatest rates of DALYs. Similarly, regions with greatest increase in mortality over the period (Andean and Central Latin America and high-income North America) also showed the greatest increase in DALYs.

For type 1 diabetes, the age-standardised death rate was higher (3·3 [95% UI 2·6 to 4·2] per 100 000 adults) for the Americas than the world's average (2·9 [2·4 to 3·6] per 100 000 adults), with a pattern of variation across regions resembling that of type 2 diabetes (table). From 1990 to 2019, the death rate increased in the Americas by 13·5% (0·7 to 27·1), in contrast to a decreasing global rate of −9·3% (−19·8 to 3·1; table). Increases occurred in the central Latin America, high-income North America, and Andean Latin American regions, with highest increase in central Latin America. Death rates decreased in the remaining regions. Age-standardised DALY rates per 100 000 adults due to type 1 disease in 2019 and trends over the period were also higher in the Americas (161·1 [130·9 to 191·6]) than globally (134·5 [110·6 to 159·7]; table). Age-standardised DALYs due to type 1 disease were highest in central Latin America and the Caribbean, and the increase was greatest in central Latin America (table). In the Americas, the age-standardised DALY rate due to type 1 diabetes increased 13·5% (2·5 to 23·1), and the all-ages DALY rate increased 21·4% (9·4 to 33·2; appendix p 80).

**Figure 1:**
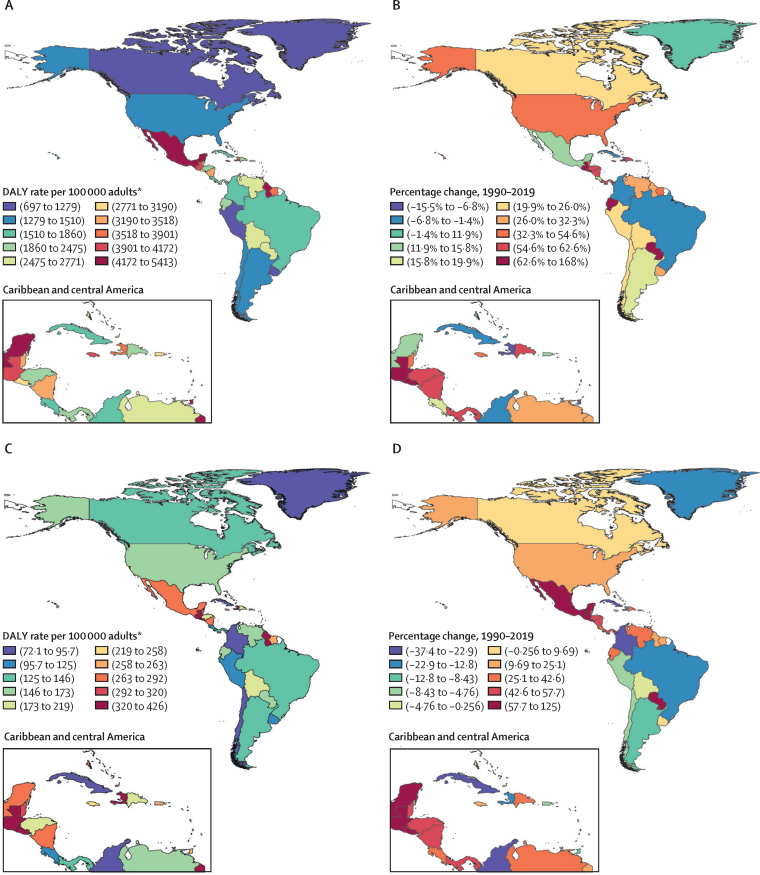
Age-standardised DALY rate in 2019 and percentage change in DALY rate from 1990–2019 for type 1 and type 2 diabetes, both sexes (A) Age-standardised type 2 diabetes DALY rate per 100 000 adults aged 20 years or older in 2019. (B) Percentage change in age-standardised type 2 diabetes DALY rate, 1990–2019. (C) Age-standardised type 1 diabetes DALY rate per 100 000 adults aged 20 years or older in 2019. (D) Percentage change in age-standardised type 1 diabetes DALY rate, 1990–2019. DALY=disability-adjusted life-year. *Aged 20 years or older.

The highest age-standardised DALY rates for type 2 diabetes burden in 2019 were in countries of central Latin America and the Caribbean (figure 1A; appendix p 81). The highest percentage changes in age-standardised DALY rates of type 2 diabetes from 1990 to 2019 were also generally seen in countries of central Latin America and the Caribbean (figure 1B). Of note, a few countries of other regions (Paraguay and Ecuador) had large increases in the burden of type 2 diabetes. Age-standardised type 1 diabetes DALY rates across countries were less heterogeneous (figure 1C, 1D). Again, some countries of the central Latin America and Caribbean regions had notably higher burden. Increases over the period were seen for various countries, notably Mexico, Guatemala, and Paraguay (appendix p 81). Countries showing greatest decreases in type 1 burden included Brazil, Colombia, and Cuba. Considering type 1 and type 2 diabetes together, the greatest increases in burden were in Guatemala (162·0 % [95% UI 119·4 to 215·1]) and El Salvador (118·7% [79·7 to 163·9]), and the largest decreases in Bermuda (−16·8% [–26·7 to −6·4]) and Trinidad and Tobago (−11·5% [–27·9 to 6·7]; appendix pp 78–79). The appendix (pp 78–79) presents the change in age-standardised DALYs from 1990 to 2019 for all countries and regions. For the Americas, 92·6% of the diabetes burden was due to type 2 diabetes (appendix pp 78–79).

**Figure 2:**
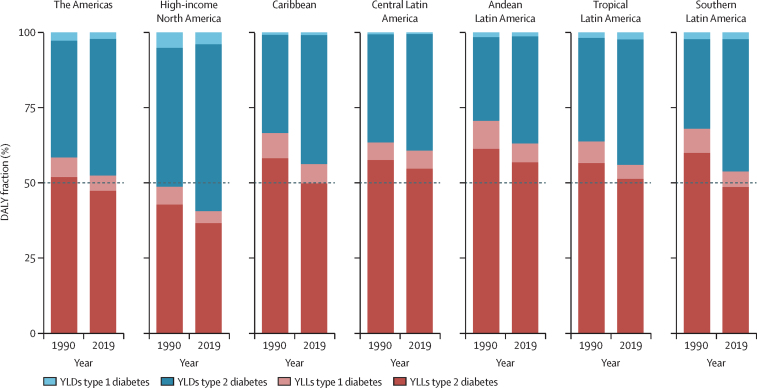
Percentage of total all-age diabetes burden expressed as disability (YLDs) and premature mortality (YLLs) among regions of the Americas in 1990 and 2019, both sexes YLDs=years lived with disability. YLLs=years of life lost. *Aged 20 years or older.

Most of the diabetes burden in the Americas in 2019 derived from premature mortality, although the fraction due to disability increased from 1990 to 2019 in all regions, approaching 50% in most regions and reaching 59% in high-income North America (figure 2). In 2019, the type 2 diabetes burden was almost equally divided between premature death (YLLs) and disability (YLDs). However, for type 1 diabetes, mortality produced considerably more than 50% of the burden, except in high-income North America (figure 2; appendix p 82). From 1990 to 2019, age-standardised YLLs remained stable or decreased in all regions except Andean and central Latin America (appendix p 82). By contrast, age-standardised YLDs progressively increased in all regions, particularly in the Caribbean, as well as in central Latin America and high-income North America (appendix p 82).

### Burden of high fasting plasma glucose

**Figure 3:**
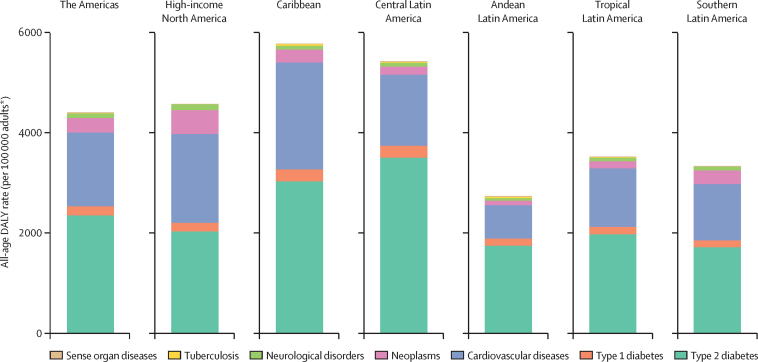
All-age DALY rate due to high fasting plasma glucose in adults (older than 25 years) in the Americas by cause in 2019 The burden includes that resulting directly from diabetes (deaths; acute decompensations, microvascular complications including chronic kidney disease, and the burden of living with diabetes) and indirectly from diabetes and lesser degrees of hyperglycaemia via the occurrence of outcomes in other disease groups. DALY=disability-adjusted life-year. *Aged older than 25 years.

In 2019, 4401 (95% UI 3685–5265) crude DALYs per 100 000 adults older than 25 years were due to high fasting plasma glucose, the metric expressing the full burden of the metabolic derangements of diabetes and lesser states of hyperglycaemia (appendix p 83). About 57% of this burden in the Americas was due to causes directly related to both type 1 and type 2 diabetes (deaths; acute decompensations, microvascular complications including chronic kidney disease, and the burden of living with diabetes; figure 3). The remaining 43% was due to indirect causes, expressed by GBD 2019 as the burden of hyperglycaemia (the majority of this being diabetes) manifested through cardiovascular diseases, neoplasms, neurological disorders (dementia), tuberculosis, and additional sense organ diseases (cataract and glaucoma; figure 3).

The level of burden was notably higher in the Caribbean, central Latin America, and North America, the three northern-most regions, and lower in Andean, southern, and tropical Latin America, the southern-most three regions.

### Determinants of diabetes burden: SDI, HAQ Index, and diabetes prevalence

**Figure 4:**
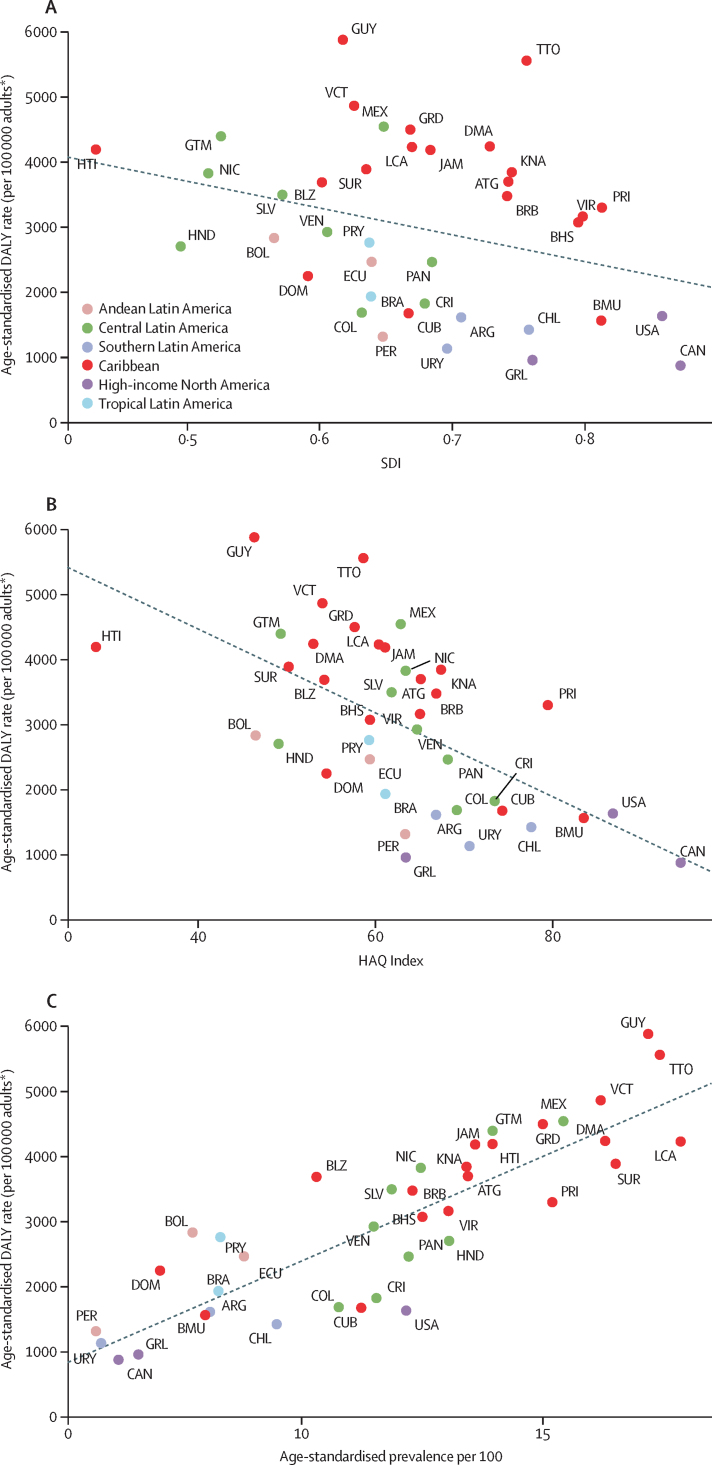
Correlation of the SDI (A), the HAQ Index (B), and prevalence of diabetes (C) with the age-standardised DALY rate due to diabetes in the Americas, 2019 DALY=disability-adjusted life-year. HAQ Index=Healthcare Access and Quality Index. SDI=Socio-demographic Index. *Aged 20 years or older.

The level of development of a country, assessed by diabetes burden is shown in figure 4A. Age-standardised DALY rates decreased 412 per 100 000 for each 10% increase in SDI (p=0·054, *r*^2^=9·7%; appendix p 84).

Access to and quality of health care was also associated with diabetes burden (figure 4B). DALYs decreased 645 per 100 000 with each 10% increase in the HAQ Index (p<0·0001, *r*^2^=35·7%; appendix p 84). Overall diabetes burden was highest for nations with HAQ Index values of less than 60% (Haiti, Guyana, Guatemala, Suriname, Belize, and several smaller Caribbean countries). These associations were generally similar when only type 1 diabetes burden was considered (appendix p 88).

Burden of diabetes was strongly associated with diabetes prevalence (p<0·0001, *r*^2^=0·72; figure 4C). Countries with highest prevalence and burden were concentrated in the Caribbean and central Latin America.

Given this strong association between diabetes burden and prevalence, we present findings on diabetes prevalence across the region. Age-standardised prevalence for both type 1 and type 2 diabetes in the Americas was higher than that seen globally in 2019. Type 2 diabetes prevalence in Americas (10·4 [95% UI 9·6–11·1]) was 25% higher than that seen globally (8·3 [7·6–9·1]) and ranged from 6·7% (6·1–7·3) in Andean Latin America to 13·4% (12·3–14·4) in central Latin America. Type 1 diabetes prevalence in the Americas (0·6 [0·5–0·7]) was 50% higher than global prevalence (0·4 [0·3–0·5]). The pattern of type 1 diabetes prevalence across regions differed considerably from that of type 2 diabetes, being highest in high-income North America (1·0%), mid-level in southern (0·5%) and tropical (0·5%) Latin America, and lowest (0·2–0·3%) in the remaining regions (appendix p 85).

From 1990 to 2019, the prevalence of type 2 diabetes increased in the Americas by 45·2% (95% UI 42·5–48·4), which was similar to the increase seen globally (48·9% [47·0–50·6]). The increase was highest in southern Latin America (79·8% [68·5–91·3]), Andean Latin America (68·3% [61·7–75·1]), and high-income North America (54·0% [48·1–60·8]). Regarding type 1 diabetes, a condition with much lower prevalence, the increase ranged from 12·0% (4·2–19·4) to 45·9% (38·3–52·9) across regions, compared with a 29·5% (24·6–34·8) increase globally. These increases in prevalence were accompanied by increases in the incidence of both type 1 and type 2 diabetes, 24·2% and 36·7%, respectively (appendix p 87).

### Determinants of diabetes burden: risk factors

**Figure 5:**
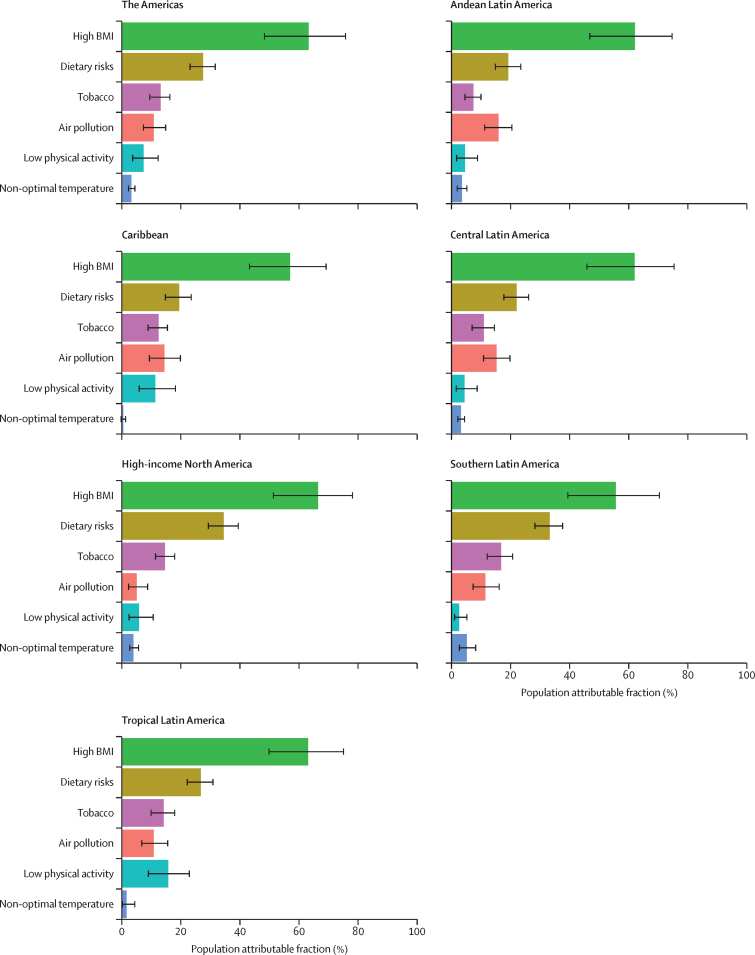
Crude population attributable fraction of DALYs due to type 2 diabetes for the main risk factors for diabetes identified by GBD 2019, by region Error bars indicate 95% uncertainty intervals. GBD=Global Burden of Diseases, Injuries, and Risk Factors Study.

As indicated by the PAF of burden (figure 5), the main risk factor for type 2 diabetes was high BMI in 2019 in the Americas, ranging from 55·8% (39·6–70·5) in the Southern LA to 66·4% (51·3–78·1) in high income North America. Dietary factors—particularly excess consumption of red and processed meat and sweetened beverages, and insufficient consumption of whole grains, fruits, fibre, and seeds and nuts—were second in importance, responsible for 27·5% (23·1–31·6) of DALYs. Air pollution and tobacco alternated as the next most important factors in most regions, with low physical activity being third in importance in tropical Latin America (15·7% [9·0–22·8]) and fourth in high-income North America (5·8% [2·4–10·6]; appendix p 87).

Although BMI was the most important risk factor for type 2 diabetes burden in 2019, we found no correlation between type 2 diabetes prevalence and the prevalence of obesity in 2019 (*r*^2^=0·013 for men and 0·03 for women; appendix p 90) or in 1990 (*r*^2^=0·05 for men and 0·055 for women).

## Discussion

The burden of diabetes in the Americas is large, heterogeneous, and increasing, especially for type 2 diabetes. Several countries in central Latin America and the Caribbean are particularly affected. Prevalence of diabetes explained most of the variability in diabetes burden across countries, with access to better quality health care and sociodemographic development also being important determinants. Well characterised risk factors for type 2 diabetes—high BMI and dietary risks—were the major determinants of type 2 diabetes burden. Although mortality continued to constitute most of the diabetes burden, the burden from disability had progressively increased from 1990 to 2019.

The burden of diabetes for the Americas in 2019—5·9% of all deaths and 2266 DALYs per 100 000 adults directly from diabetes and chronic kidney disease due to diabetes, and approximately double that considering the whole burden of high fasting plasma glucose—places diabetes among the principal causes of loss of health in the Americas. The burden was considerably higher in the most affected regions of central Latin America and the Caribbean. Our findings for the Americas parallel the published GBD 2016 estimation that diabetes (when also considering burden from chronic kidney disease due to diabetes) will be, by 2040, the second leading disease causing deaths in the low-income and middle-income countries of Latin America and the Caribbean,^13^ and that high fasting plasma glucose is currently the world's third leading risk factor for disease burden.^9^

The current burden of diabetes and hyperglycaemia, as fully expressed through high fasting plasma glucose and without adjustment for age, is currently centred in the northern three regions. The bulk of this burden (92·6%) is due to type 2 diabetes, the prevalence of which increased by 45·2% since 1990. Considering the tight association of diabetes burden with its prevalence, the fact that the greatest increases in prevalence were observed in currently lower-prevalence Andean (68·3%) and southern (79·8%) Latin American regions indicates that the diabetes burden in the Americas is now expanding.

The scenario of high and increasing prevalence of both diabetes types, especially type 2, observed in the Americas is consistent with population-based, pooled analyses of the NCD Risk Factor Collaboration showing a rising prevalence in the Americas^8^ and globally.^14^ The epidemiological transition, which extends longevity, is in high gear in low-income and middle-income countries,^15^ including most nations of the Americas. Population ageing raised the 27·4% increase in the type 2 diabetes age-standardised burden to a 59·2% increase in its crude burden, demonstrating that the increase in true burden is even larger. Because incidence is an important driver of prevalence, it is particularly concerning that, in contrast to the downturn in incidence in some high-income countries since 2010,^16^ the regions of the Americas show large increases in the incidence of type 2 diabetes.

Similar to the findings of the NCD Risk Factor Collaboration,^8^ we found no association between the prevalence of obesity and diabetes. We do not believe that the lack of this ecological association means that obesity does not affect diabetes development in the region. First, the narrower range of national obesity prevalences found across nations contrasts with the broad range of BMIs seen across individuals in cohort studies. In fact, when we examined this association globally, which produced a wider range of obesity prevalence, the *r*^2^ values were slightly larger. Confounders such as poverty, early life deprivation, and other diabetes risk factors could also be at play.

The age-adjusted fraction of the overall diabetes burden due to type 1 diabetes was small. Of note, however, type 1 diabetes death and DALY rates in the Americas increased at a rate greater than the global average. The high prevalence of type 2 diabetes risk factors such as smoking, dietary inadequacies, and physical inactivity might explain part of this excess, as these risk factors are also important prognostic factors for type 1 diabetes.^17^ Thus, population-based approaches to reduce these risk factors are also relevant to control the burden of type 1 diabetes.

Lesser socioeconomic development, as measured by the SDI, most present in central Latin America and the Caribbean, explained part of the difference in burden across countries, suggesting that gain can be achieved by further broad socioeconomic development. But the finding also raises concern, as the less socioeconomically developed countries expected to experience the largest increases in burden from diabetes and its complications will be those least prepared to deal with the consequences, given their limited ability to increase spending on health, and their health systems, which are ill prepared to manage diabetes and its complications.^18^ If these countries cannot reduce burden, the resultant direct and indirect costs of diabetes and other NCDs are estimated to be of sufficient size to significantly decrease their rate of economic growth.^19^, ^20^

Access to and quality of health care explained an important part of the differences in burden across countries in the Americas. Additionally, advances in health care seem to have blunted the translation of increased prevalence over time into increased premature mortality (YLLs) in most regions. Expansion of national health systems providing greater access to evidence-based diabetes care, especially at the primary-care level, could help to minimise the burden due to increasing prevalence. WHO has provided protocols for minimal, cost-effective interventions^21^ that can help with organising care in the most resource-constrained countries.

A special focus should be on decreasing the most amenable cause of burden due to type 1 diabetes—deaths from acute complications—through improved access and quality of care in most countries. Policies aimed at universal access to low-cost insulin and oral antidiabetic medications, and to basic diabetes care are mandatory. Additionally, as mortality declines, the number of older individuals living with the multiple complications common to long-standing diabetes will increase, and cost-effective approaches to attending their needs will be necessary.

Given the large role of type 2 diabetes prevalence in causing burden, more attention to the primary prevention of type 2 diabetes is of utmost importance. The deleterious effects of the main type 2 diabetes risk factors here addressed—excess weight, dietary risks, tobacco, physical inactivity, and air pollution—are well documented in systematic reviews of multiple cohort studies.^9^, ^22^, ^23^ Obesity, documented in cohort studies to be the most important driver, is increasing in all the regions of the Americas.^8^, ^22^, ^24^ Dietary risks, second in importance, reflect dietary patterns of insufficient consumption of foods shown to be protective against diabetes, such as whole grains and fruits, coupled with an excess consumption of deleterious foods, especially red and processed meat and sweetened beverages.^9^, ^23^ This dietary profile has occurred in parallel with the growing globalisation of the food industry. Food, while increasingly available, is also increasingly different from its traditional presentations, given the rising consumption of ultra-processed foods, which are vectors of dietary risks.^25^, ^26^, ^27^ The region of the Americas is the epicentre of this phenomenon. Canada and the USA lead the world in annual retail sales (kg per capita) of ultra-processed foods.^25^ Sales over 2000–2013 increased around the world (3·1% per year in Latin American countries), with sales of carbonated beverages in Latin America exceeding those in North America by 2013. Discounting high-income regions of the world, Latin America was the region with highest per-capita sales of ultra-processed foods in 2013.^25^

Low agency (ie, requiring little or no personal effort), population-based interventions such as taxes and incentives, combined with expanded health education to combat diabetes risk factors, seem to offer the greatest promise of success.^28^, ^29^ WHO's Best Buys^30^ include several examples of such actions. Policies aimed to decrease obesity and control dietary risks must be the major focuses of such primary prevention programmes. Greater actions to increase physical activity and decrease smoking are also mandatory.

Legislation leading to stricter control of air pollution is also necessary, as demonstrated by recent findings showing air pollution to be a major diabetes risk factor.^31^ Additionally, multiple studies suggest that persistent organic pollutants, which are proven endocrine disruptors, increase the risk of diabetes.^32^ If these findings are confirmed and causative pathways elucidated, further actions to expand control of pollutants are necessary.

Our study has limitations, many of which are inherent to the GBD global approach. As shown in the appendix (p 75), many locations in the Americas lack high-quality data, leading to imprecision in results when they are calculated based on data from other countries adjusted through covariates, which is more often the case for type 1 diabetes and for the assessment of non-fatal burden. Although our focus on region, rather than country, reduces the effect of sparse data, the frequently wide UIs we report indicate that caution is necessary when interpreting comparisons across countries. The addition of new and better-quality data will be important to produce more reliable estimates in the future. By making data adjustments for less specific ICD codes, we try to take away changes over time that may be due to improved diagnostic capabilities. However, all data adjustments can lead to so-called measurement noise. In GBD, we make a conscious choice to make use of as many data sources as we can and accept the additional noise from these adjustments as preferable to an approach that would make use of a much more limited set of high-quality data sources only. Although use of alternative case definitions of diabetes, once adjusted, makes more data available for analysis, the data adjustments could have introduced measurement bias. Finally, as the GBD uses the HAQ Index as a covariate to predict the fatal and non-fatal burden of diabetes in countries with sparse data, the association of this index with burden might be overestimated.

Despite these limitations, the analyses of diabetes burden for the Americas based on GBD data here presented offer important information for policy making in the region. More than 100 years after the discovery of insulin, much remains to be done to ensure access to quality diabetes care to prevent complications and minimise disability. This is particularly relevant for many countries in the Caribbean and central Latin America because these two regions have the greatest burden of diabetes in the Americas. A greater focus on population-wide diabetes prevention is urgently needed, as screening for intermediate hyperglycaemia and subsequent lifestyle modification for these individuals at high risk, although cost-effective, has little population impact. For example, the early diagnosis and treatment strategy applied in the UK was estimated to produce a relative decrease in the long-term incidence of diabetes of less than 3·5%.^33^

Thus, curtailing the diabetes epidemic, a major challenge for the Americas, will require greater use of population-based strategies, given their wide reach and low costs. Ways to defuse the political controversy about the extent to which government should regulate market activity in the interest of public health and to constrain the economic interests that block the implementation of these policies must be developed.

Finally, the major gaps in data here identified highlight the need for improved surveillance capacity, especially with regard to the characterisation of type of diabetes and to the assessment of non-fatal diabetes burden.

## Data sharing

To download the data used in these analyses, please visit the Global Health Data Exchange http://ghdx.healthdata.org/gbd-2019/data-input-sources.

## Declaration of interests

T Bärnighausen reports support for the present manuscript from Alexander von Humboldt Foundation, Wellcome Trust, German Research Foundation, and US National Institutes of Health (National Institute of Allergy and Infectious Diseases, National Institute on Aging, and Fogarty International Center), paid to his institution. I Filip and A Radfar report support from Avicenna Medical and Clinical Research Institute, outside the submitted work. J A Singh reports consulting fees from Crealta/Horizon, Medisys, Fidia, PK Med, Two labs, Adept Field Solutions, Clinical Care Options, Clearview Healthcare Partners, Putnam Associates, Focus Forward, Navigant Consulting, Spherix, MedIQ, Jupiter Life Science, UBM, Trio Health, Medscape, WebMD, and Practice Point Communications, the National Institutes of Health, and the American College of Rheumatology; payment or honoraria for lectures, presentations, speakers' bureaus, manuscript writing or educational events from Simply Speaking; support for attending meetings or travel from the steering committee of OMERACT; participation on a Data Safety Monitoring Board or Advisory Board with the US Food and Drug Administration Arthritis Advisory Committee; leadership or fiduciary role in board, society, committee or advocacy group, paid or unpaid, with OMERACT as a steering committee member, with the Veterans Affairs Rheumatology Field Advisory Committee as Chair (unpaid), and with the UAB Cochrane Musculoskeletal Group Satellite Center on Network Meta-analysis and editor and director (unpaid); stock or stock options in TPT Global Tech, Vaxart Pharmaceuticals, Atyu Biopharma, Adaptimmune Therapeutics, GeoVax Labs, Pieris Pharmaceuticals, Enzolytics, Seres Therapeutics, Tonix Pharmaceuticals and Charlotte's Web Holdings, and previously owned stock options in Amarin, Viking and Moderna Pharmaceuticals; all outside the submitted work.
