# Supplementary material for: Burden of diabetes and hyperglycaemia in adults in the Americas, 1990–2019: a systematic analysis for the Global Burden of Disease Study 2019
Source: Lancet Diabetes Endocrinol. 2022 Sep;10(9):655–67. doi: 10.1016/S2213-8587(22)00186-3 (PMC9399220; doi:10.1016/S2213-8587(22)00186-3)

THE LANCET
Diabetes & Endocrinology

Supplementary appendix

This appendix formed part of the original submission and has been peer reviewed. We post it as supplied by the authors.

Supplement to: GBD 2019 Diabetes in the Americas Collaborators. Burden of diabetes and hyperglycaemia in adults in the Americas, 1990–2019: a systematic analysis for the Global Burden of Disease Study 2019. *Lancet Diabetes Endocrinol* 2022; published online July 15. https://doi.org/10.1016/S2213-8587(22)00186-3.

Appendix to "Burden of diabetes and hyperglycaemia in adults in the Americas: a Global Burden of Disease 2019 study"

Portions of this Appendix have been reproduced or adapted from Vos et al.,[1] Wang et al.,[2] Lozano et al.,[3] and Murray et al.[4]

Table of Contents

Methods Appendix 5

Section 1. Overview of GBD methodology 7

Section 2. Multiple approaches to the burden of diabetes and high fasting plasma glucose 8

Section 3. All-cause mortality 9

Section 4. GBD 2019 Causes of Death database 11

Section 4.1. Steps in data input 12

Section 4.1. Step 1: Standardise input data 12

Section 4.1. Step 2: Map to GBD cause list 12

Section 4.1. Step 3: Split age-sex groups 13

Section 4.1. Step 4: Correct for miscoding of Alzheimer's and other dementias, Parkinson's disease, and atrial fibrillation and flutter 13

Section 4.1. Step 5: Redistribute 14

Section 4.1. Step 6: Correct HIV/AIDS misclassification 14

Section 4.1. Step 7: Scale strata to province 14

Section 4.1. Step 8: Correct post-redistribution problems 14

Section 4.1. Step 9: Drop VR country-years or mark as non-representative 14

Section 4.1. Step 10: Aggregate causes 15

Section 4.1. Step 11: Remove shocks and HIV/AIDS maternal adjustments 15

Section 4.1. Step 12: Apply noise-reduction algorithms 15

Section 4.1. Step 13: Identify outliers in the Cause of Death database 15

Section 4.2. Data star rating for the quality of VR 16

Section 5. Cause of death modelling methods 19

Section 5.1. CODEm 19

Section 5.2 Causes modelled outside of CODEm 20

Section 5.3. CoD Correct 20

Section 5.4. Years of life lost calculation 20

Section 5.5 GBD world population age standard 21

Section 5.6 Statistical analyses 21

Section 6. Specific CoD modelling descriptions 21

Section 6.1 Diabetes mellitus 21

Section 6.1.1. Overall diabetes mellitus 22

Section 6.1.2. Diabetes mellitus type 1 and type 2 27

Section 6.2. Chronic kidney disease 33

Section 6.2.1. Input data ... 33
Section 6.2.2. Modelling strategy ... 33
Section 6.2.3. Covariate influences ... 34
Section 6.2.4. Chronic kidney disease subtypes, including those due to type 1 and type 2 diabetes 35
Section 7. Non-fatal outcome estimation ... 36
Section 7.1. Estimation of prevalence and incidence by cause and sequelae by using DisMod-MR 2.1 36
Section 7.1.2. Severity distribution ... 37
Section 7.1.3 Disability weights ... 37
Section 7.1.4. Comorbidity adjustment ... 37
Section 7.1.5. YLD computation and uncertainty ... 38
Section 7.2. DALY computation and uncertainty ... 38
Section 7.3. Specific non-fatal modelling descriptions ... 38
Section 7.3.1. Diabetes mellitus ... 38
Section 7.3.2. Chronic kidney disease ... 51
Section 8. Risk factor estimation ... 56
Section 8.1. Overview ... 56
Section 8.2. Steps in calculation of burden ... 57
Section 8.3. Diagrams of steps involved in the estimation of attributable burden ... 58
Section 8.4. High fasting plasma glucose ... 60
Section 8.4.1. Case definition ... 60
Section 8.4.2. Data seeking ... 60
Section 8.4.3. Data inputs ... 61
Section 8.4.4. Data processing ... 62
Section 8.4.5. Exposure modelling ... 62
Section 8.4.6. Theoretical minimum-risk exposure level ... 63
Section 8.4.7. Relative risks ... 63
Section 8.4.7.1. Relative risks for high fasting plasma glucose (continuous risk) ... 64
Section 8.4.7.2. Relative risks for diabetes mellitus (categorical risk) ... 64
Section 8.5. Risk factors for diabetes ... 64
Section 9. Socio-demographic Index ... 64
Section 10. Universal Health Coverage (UHC) Effective Care Index ... 65
GATHER checklist ... 67
Authors' Contributions ... 70

Managing the overall research enterprise ... 70
Writing the first draft of the manuscript ... 70
Primary responsibility for applying analytical methods to produce estimates ... 70
Primary responsibility for seeking, cataloguing, extracting, or cleaning data; designing or coding figures and tables ... 70
Providing data or critical feedback on data sources ... 70
Developing methods or computational machinery ... 70
Providing critical feedback on methods or results ... 70
Drafting the work or revising is critically for important intellectual content ... 70
Managing the estimation or publications process ... 71
References ... 72
Supplementary tables and figures ... 73

Methods Appendix

List of Methods Appendix figures

Appendix Figure 1. Characterisation of burden due to diabetes and lesser states of hyperglycaemia

Appendix Figure 2. Analytical flowchart for the estimation of all-cause mortality by age and sex, and HIV/AIDS incidence, prevalence, and mortality for GBD 2019

Appendix Figure 3. Classification of national time series of vital registration and verbal autopsy data, 2010−2018

Appendix Figure 4. GBD 2019 DisMod-MR 2.1 analytical cascade

Appendix Figure 5. Analytical flowchart of the comparative risk assessment for the estimation of population attributable fractions. Ovals represent data inputs, rectangular boxes represent analytical steps, cylinders represent databases, and parallelograms represent intermediate and final results. GBD=Global Burden of Disease. SEVs=summary exposure values. TMREL=theoretical minimum-risk exposure level. PAFs=population attributable fractions. YLLs=years of life lost. YLDs=years lived with disability. DALYs=disability-adjusted life-years

Appendix Figure 6: Spatiotemporal Gaussian process regression (ST-GPR) flowchart

List of Abbreviations

Abbreviation	Meaning
5q0	probability of death from birth to age 5 years
BTL	basic tabulation list
CKD	chronic kidney disease
CoD	causes of death
CODEm	Cause of Death Ensemble modelling
COMO	comorbidity correction
CSMR	cause-specific mortality rate
CSV	comma-separated values
DALYs	disability-adjusted life-years
DisMod-MR	disease model—Bayesian meta-regression
DW	disability weights
EMR	excess mortality rate
GATHER	Guidelines for Accurate and Transparent Health Estimates Reporting
GBD	Global Burden of Diseases, Injuries, and Risk Factors Study
GHDx	Global Health Data Exchange
HAQ Index	Healthcare Access and Quality Index
ICD	International Classification of Diseases
IHME	Institute for Health Metrics and Evaluation
LDI	lag-distributed income per capita
LMER	linear mixed effects regression
MEPS	Medical Expenditure Panel Surveys
MR-BRT	meta-regression—Bayesian, regularised, trimmed
PAF	population attributable fraction
SDI	Socio-demographic Index
ST-GPR	spatiotemporal Gaussian process regression
UI	uncertainty interval
VA	verbal autopsy
VR	vital registration
WHO	World Health Organization
YLDs	years lived with disability
YLLs	years of life lost

This Appendix provides methodological details and supplemental figures and tables. Briefly, it summarises details presented principally in the Methods Appendix to "Global Burden of 369 diseases, injuries, and impairments, 1990–2019: a systematic analysis for the Global Burden of Disease Study 2019" (Named hereafter Capstone Appendix),[1] but also in the methods appendices of additional GBD 2019 publications.[2,3] Our aim is to give a comprehensive description of the analytical steps taken, with tables, figures, and specific details to make transparent our estimation processes.

Section 1. Overview of GBD methodology

The GBD 2019 applies a standard methodological approach to generate estimates for mortality and causes of death for diseases for 204 countries and territories. We grouped countries and territories into 21 regions and these into seven super-regions: 1) central Europe, eastern Europe, and central Asia; 2) high income; 3) Latin America and the Caribbean; 4) north Africa and the Middle East; 5) south Asia; 6) southeast Asia, east Asia and Oceania; and 7) sub-Saharan Africa. GBD organises causes of death based on the GBD cause list, which is hierarchical, comprising four levels:

At Level 1, there are three cause groups: communicable, maternal, neonatal, and nutritional diseases; non-communicable diseases, including diabetes and chronic kidney disease; and injuries.

At Level 2, these Level 1 groups are subdivided into 22 cause groups, with diabetes and chronic kidney disease (CKD) grouped together.

At Level 3, diabetes mellitus and chronic kidney diseases are disaggregated.

At Level 4, type 1 diabetes, type 2 diabetes, chronic kidney disease due to type 1 diabetes, and chronic kidney disease due to type 2 diabetes are disaggregated to contains the finest detail for these causes captured in GBD 2019.

GBD publications comply with the Guidelines for Accurate and Transparent Health Estimates Reporting (GATHER) recommendations.[5] The steps in our analytical procedures and detailed data sources can be found in the appendices of the GBD 2019 publications cited above (with Table 1 for the GATHER checklist). To check the GATHER Statement, visit the GATHER website under GATHER Statement.

GBD 2019 synthesises a large and growing number of data input sources, including surveys, censuses, vital statistics, and other health-related data sources which are used to estimate mortality. The input sources are accessible through an interactive citation tool available in the Global Health Data Exchange (GHDx; http://ghdx.healthdata.org/). This tool allows users to view and access GHDx records for input sources and export a comma-separated value (CSV) file that includes metadata, citations, and information on where data were used in GBD. Citations for specific GBD components, causes and risks, and locations can also be found with this tool. As required by GATHER, additional metadata for input sources are available through the citation tool as well.

The GBD permits visualisation of its results online. All GBD 2019 online data visualisations are available at https://vizhub.healthdata.org/gbd-compare/, which provides results for all GBD health metrics. Core summary GBD 2019 results, including for deaths, can be downloaded in tabular form with the GBD's data download tool, available at http://ghdx.healthdata.org/gbd-results-tool. Data above a certain size cannot be viewed online but can be downloaded. Depending on the size of the download, users may

need to enter an e-mail address; a download location will be sent to them when the files are prepared. Input data used in the cause specific models by location can be found at the links below:

http://ghdx.healthdata.org/gbd-2019/data-input-sources?components=4&causes=587&locations=103

http://ghdx.healthdata.org/gbd-2019/data-input-sources?components=4&causes=589&locations=103

http://ghdx.healthdata.org/gbd-2019/data-input-sources?components=5&causes=587&locations=103

https://vizhub.healthdata.org/epi/

https://vizhub.healthdata.org/cod/

Section 2. Multiple approaches to the burden of diabetes and high fasting plasma glucose

As shown in Appendix Figure 1, the GBD framework 8ecognizes type 1 and type 2 diabetes as diseases with their own complications and as distinct causes of chronic kidney disease (CKD). Within its list of risk factors, GBD includes hyperglycaemia, encompassing diabetes range and lesser levels, as an all-inclusive measure, capturing the total burden of elevated glucose metabolism – that directly caused by diabetes, that due to CKD caused by diabetes, and that resulting from other diseases (eg, ischaemic heart disease and stroke) for which diabetes increases risk of occurrence. It also encompasses the much smaller effects of levels of hyperglycaemia conferring risk but not reaching the threshold for diabetes.[6] The risk factor measuring this conjoint disease burden is called high fasting plasma glucose (HFPG). Supplementary Figure 1 summarises this multiple approach to burden.

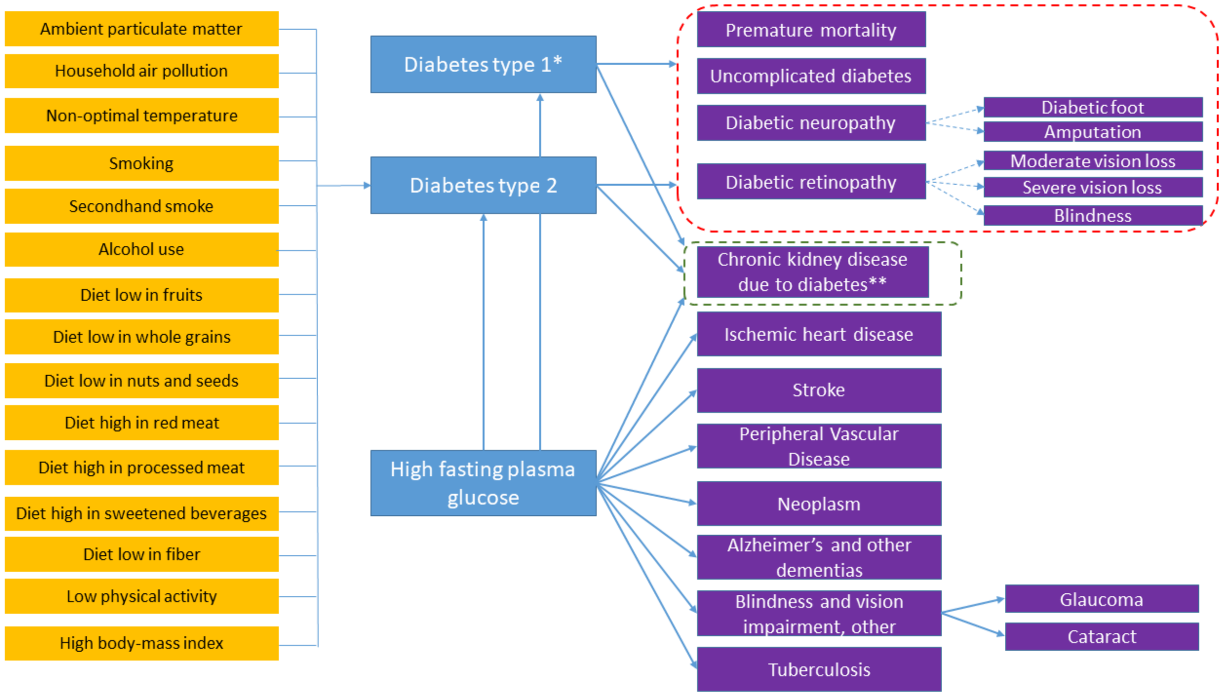

Appendix Figure 1. Characterisation of burden due to diabetes and lesser states of hyperglycaemia. The burden of diabetes and lesser-range hyperglycaemia through the additional diseases which are linked with solid lines emanating from high fasting plasma glucose is captured only through high fasting plasma glucose.

*Non-optimal temperature is considered a risk factor for type 1 diabetes.

** Chronic kidney disease due to diabetes is modelled within the chronic kidney disease framework.

Section 3. All-cause mortality

The calculation of all-cause mortality estimates for all GBD age groups, by sex, for all locations and years is described in detail in the Methods Appendix to the 2019 GBD publication “Global, regional, and national age-sex-specific fertility, mortality, and population estimates, 1950–2019: a comprehensive demographic analysis for the Global Burden of Disease Study 2019”.[2]

We calculated all-cause mortality based on the integration of data from a diverse set of sources. To estimate child mortality, we used data from vital registration (VR) systems, sample registration systems, and disease surveillance point systems, household surveys (complete and summary birth histories), censuses (summary, and on rare occasions, complete birth histories), and demographic surveillance sites. To estimate adult mortality, we used, among others, VR systems and surveys and censuses from which we extracted household death recall data.[2]

Calculations were complicated by the fact that not all countries and territories have complete vital registration (VR) systems recording the event of death or periodic censuses. Thus, our processes adjusted for the completeness (quality) of available VR data.[2]

We estimated incompleteness in VR sources for deaths under age 5 in mixed effects non-linear models, as described in section 2.2.6 of the Methods Appendix to “Global, regional, and national under-5 mortality, adult mortality, age-specific mortality, and life expectancy, 1950–2017: a systematic analysis for the Global Burden of Disease Study 2017”.[7] In this process, for each country, we initially relied on expert opinion to choose a source, or combination of sources, which were believed to be the least biased. If a country had a VR system which we deemed to be complete, this was the reference source. If a country did not have a complete VR system, but had estimates from complete birth histories, these were used as the reference source. If a country had neither of these types of data, or complete birth histories estimates were deemed unreliable, we assigned the surveys conducted after 1950 (in combination) as the reference. Incomplete VR data were not included. Additionally, in many countries, we chose alternate surveys as the reference. For accurate estimation, it was important to have local knowledge on specific data sources’ accuracy. All-cause mortality experts drew from their familiarity with data quality to help us to choose the reference category.[2]

To determine incompleteness of sources at other ages, we next combined our findings of under-5 VR data completeness with death distribution methods to estimate completeness for adults aged 15 to 59. Here, we used the three death distribution methods most common in demography: generalised growth balance, synthetic extinct generation, and a combined approach, which estimates completeness by comparing the age distribution of the population between two censuses with the age distribution of deaths between those same censuses. We also applied two additional death distribution methods that utilise the GBD Bayesian population model.[2]

As shown in Appendix Figure 2, aside from estimating completeness, five major methodological tasks were executed in estimating all-cause mortality: estimating the probability of death between birth and age 5 years (5q0); estimating the probability of death between age 15 years and 60 years (45q15); estimating a complete set of age-specific mortality rates; estimating HIV mortality; and producing final estimates of age-specific mortality, including HIV mortality and fatal discontinuities. Estimates of overall mortality by age, sex, location, and year were the outputs of this process. These estimates were used for ages 15 and above, and a combination of these under-5 and adult estimates produced completeness estimates to be used for ages 5 to 9 and 10 to 14.[2]

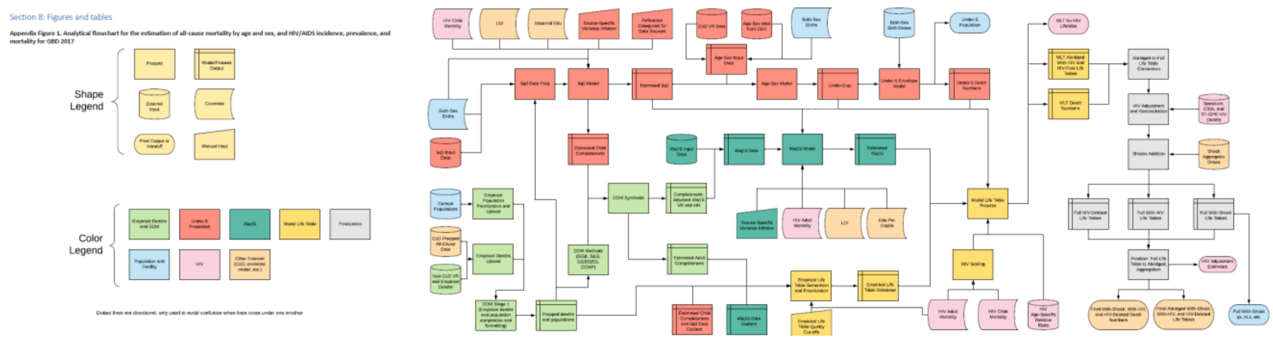

Appendix Figure 2. Analytical flowchart for the estimation of all-cause mortality by age and sex, and HIV/AIDS incidence, prevalence, and mortality for GBD 2019

Section 4. GBD 2019 Causes of Death database

Data sources for causes of death were obtained from vital registration systems, verbal autopsies, and other surveillance systems for 1990–2019.[8]

All available data on causes of death (CoD) data are standardised, based on International Classification of Diseases (ICD) 9 and 10 code mapping for diabetes and other GBD causes, and pooled into a single database used to generate cause-specific mortality estimates by age, sex, year, and geography. This process passes through several steps which are outlined below. Appendix Figures 1 and 2 of the Capstone Appendix show the high-level view of data inputs, analytical steps, and outputs of the causes of death (CoD) analysis frame.[1]

The CoD database contains seven types of data sources (Capstone Appendix Table 3), including vital registration (VR), verbal autopsy (VA), sibling history, and survey/census. In countries with complete VR systems, there is no need to use any other data source. Less than half the world's population has deaths captured in a VR system, therefore, for countries with incomplete VR systems, vital statistics for causes of death may be supplemented withother data types (Capstone Appendix Figure 3).[1]

A majority of the CoD data is VR data obtained from the World Health Organization (WHO) Mortality Database, a compilation of data submitted to WHO by individual countries. VR is also obtained from country-specific mortality databases operated by official offices. Each cause is coded directly to the most detailed CoD when possible, whereas cause codes in data tabulated by International Classification of Disease (ICD) are coded to aggregated cause groups.

Many countries use ICD Tabulation lists. The ICD tabulation lists include the ICD-9 Basic Tabulation List (BTL), the ICD-10 Mortality Tabulation, the Russia Tabulation, and the India Medical Certification of Cause of Death. Two of the drawbacks in using tabulation lists are discrepancies in the accuracy of death counts and lack of detail due to aggregated cause groups. There are instances where the sum of deaths in chapter subtotals are not equal to the sum of cause groups within the chapter. To account for any missing or duplicate deaths reported within the cause groupings, death counts are systematically adjusted by calculating the differences between subtotals and sub-causes within the cause groups. Any differences are assigned to a remainder cause group. To account for the lack of cause code detail, select cause groups are disaggregated to create a complete cause list.[1]

Sample registration systems are expanding in several countries and are key sources of data in Indonesia and India, as further detailed in the Capstone Appendix. In countries without VR systems, verbal autopsy (VA) studies are a viable data source to inform CoD. Data are obtained by trained interviewers who use a standardised questionnaire to ask relatives about the signs, symptoms, and demographic characteristics of recently deceased family members. CoD is assigned based on the answers to the questionnaires. VA data are highly heterogeneous: studies use different instruments, different cause lists (from single causes to full ICD-cause lists), different methods for assigning CoD, different recall periods, and different age groups. Cultural differences may also affect the interpretation of specific questions. CoD validity must be considered when mapping to a GBD cause. VAs are likely accurate in assigning CoD to road injury or homicide but less accurate for causes requiring medical certification, such as diabetes or chronic kidney disease.[1]

Section 4.1. Steps in data input

Processing of input data involves several steps, as follows:

Section 4.1. Step 1: Standardise input data

The input data to the CoD database are received in various formats and must be standardised to run through central CoD machinery to then upload to the database. Raw data inputs come from data sources such as mortality databases, literature reviews, or reports. Usable data sources must have a clear sample size of the number of deaths in the population and exhaustive cause lists. The complexity of the data cleaning process varies drastically across data sources. For VR microdata with the location, age, sex, year, and ICD-coded cause of every death, very little effort is necessary to standardise it into a consistent structure. Other sources may require weeks of careful review to accurately extract scans of hardcover CoD reports into spreadsheets that can be transformed and standardised.[1]

At this point, data are assigned source identifiers so that they can be linked to the GHDx and cited appropriately. Any aggregate age and sex categories are flagged for age-sex splitting. The methods of cause-of-death assignment and data collection are reviewed to determine which source type to assign;for example, we distinguish sibling history data from surveys with a VA module. Only data at the most detailed level of the GBD location hierarchy are used. Documentation from the source is reviewed to determine if the population is representative of the location or only a subset of the population in that location. Data sources representing a subset of the population are flagged as non-representative; this flag is used by Cause of Death Ensemble modelling (CODEm) to increase the variance associated with such datapoints.[1]

Finally, diagnostics are reviewed at this stage to avoid sending cleaning errors downstream. We review cause-specific deaths for each demographic group to ensure the data are reasonable. For example, it is unlikely that deaths from neonatal causes occur in age groups over 1 year. All death totals are compared with the sum of cause-specific deaths to ensure the observed deaths are accounted for and sample size is complete.

CoD in tabulated VR data are condensed into aggregated groups, some of which can be mapped directly to GBD causes, while other aggregated cause groups are not informative and cannot be mapped to them. To correct for this, aggregated causes were mapped and split onto multiple ICD-8, ICD-9, and ICD-10 detail causes, or targets, based on the ICD groupings within the aggregated causes.ICD-8, ICD-9, and ICD-10 detail codes serve as targets because they are the highest-quality VR data and enable the calculation of proportions used to split the aggregated cause data into detailed causes. The proportions of deaths from nearby countries within the super-region were used to fill in data gaps as they were likely to have similar CoD trends.[1]

We determined the targets based on detailed causes missing from the tabulated cause list. For any cause and demographic group for which we lacked ICD detail, global proportions were used. State splitting and calculation of non-maternal deaths complete this step.[1]

Section 4.1. Step 2: Map to GBD cause list

In GBD 2019, we used 439 maps to translate causes found in the input data to the GBD 2019 cause list. This included 31 maps for VR data, 314 for VA data sources, and 98 for other data types. The largest, and

most universal, maps used were those for ICD-9 and ICD-10 VR data. Our mapping process enabled us to compare these various data sources across demographic groups.[1]

In GBD 2019, we developed additional maps to translate ICD codes found in the input data that are non-underlying causes to appropriate target codes based on the levels of the GBD cause list.[8] These garbage codes were mapped to Levels 1-4 of the GBD cause list according to the following criteria:

- Level 1 garbage codes include all codes for which a Level 1 GBD cause cannot be directly assigned. For example, the underlying causes of "sepsis" or "peritonitis", if not specified in the data, could be an injury, a non-communicable disease, or a type of communicable disease. In these cases, deaths will be redistributed across all three of the Level 1 causes. In addition, deaths coded to impossible or ill-defined causes of death (including "senility" and "unspecified causes") fall into this category, as they will be redistributed onto all causes.
- Level 2 garbage codes include all codes that can be assigned within the same Level 1 GBD cause, being redistributed onto Level 2 causes.
- Level 3 garbage codes include all codes that can be assigned within the same Level 2 GBD cause, being redistributed onto Level 3 causes.
- Level 4 garbage codes include all codes (eg, "unspecified diabetes mellitus") that can be assigned within the same Level 3 GBD cause, being redistributed onto Level 4 causes.

Section 4.1. Step 3: Split age-sex groups

Different sources, particularly VA studies, report deaths for a wide range of age groups with varying intervals. For the analysis of CoD, we mapped these different age intervals to the GBD standard set of age groups. The Capstone Appendix displays formulas used for this purpose. In some cases, deaths are reported for an aggregate age group for both sexes combined. The task in thiscase is more complicated, but the same principle can be applied. In this case, we assumed that the relative risks of death by age and sex are constant.[1]

We next adjusted separately for estimated adult and child VR completeness. Location-year-age-sex-cause-specific deaths and population were then aggregated across all location-years, to produce cause-specific mortality rates by age and sex. These were used to determine the risk of death at any age relative to any reference age group[1].

Occasionally, data sources include deaths by a cause for which medical consensus exists that death is impossible for the sex and age. For example, some number of deaths may be attributed to cervical cancer in males, or to maternal causes in children younger than 10 years. We have constructed a conservative list of age-sex restrictions. When deaths violate these restrictions, we redistribute them proportionally onto all causes. All restrictions are included, in the Capstone Appendix, in Appendix Table 5, Restrictions on age and sex by cause for GBD 2019.[1]

Section 4.1. Step 4: Correct for miscoding of Alzheimer's and other dementias, Parkinson's disease, and atrial fibrillation and flutter

This step, less relevant for diabetes and CKD calculations, is described in the Capstone Appendix.[1]

Section 4.1. Step 5: Redistribute

A crucial aspect of enhancing the comparability of data for CoD is to deal with uninformative, so-called garbage codes. Garbage codes to which deaths were assigned should not be considered as the underlying CoD – for example: "heart failure", "ill-defined cancer site", "senility", "ill-defined external causes of injuries", and "septicaemia". The methods for redistributing these garbage-coded deaths were outlined in detail in Johnson SC.[8] Because of the disparate nature of HIV/AIDS mortality across space and time, dynamic redistribution of HIV/AIDS-related garbage codes was applied.[8]

For each redistribution package, we defined the "universe" of data as all deaths coded to either the package's garbage codes or the package's redistribution targets for each country, year, age, and sex. We then ran a regression, the formula for which is given in the Capstone Appendix, separately for each target group and sex. In GBD 2019, we updated the regressions for stroke and diabetes. We dropped the proportion of garbage from the regression formula and ran regression on high-quality, low proportion garbage data (4/5 stars, <50% GC). We also included all covariates included in the CODEm models for both stroke and diabetes.[8]

Section 4.1. Step 6: Correct HIV/AIDS misclassification

This step, little relevant to diabetes and CKD calculations, is described in detail in the Capstone Appendix.[1]

Section 4.1. Step 7: Scale strata to province

This step, related specifically to calculations related to China, is described in detail in the Capstone Appendix.[1]

Section 4.1. Step 8: Correct post-redistribution problems

This step ensures that the detail of the cause list at this point in the data prep process is reasonable given the detail of the original data source and the methods by which the CoD was assigned. Two primary corrections are applied. First, any cause that is purely an artifact of the redistribution machinery targeting too detailed a cause is aggregated up to the parent cause. Second, a "bridge map" is applied over a certain set of sources to ensure that they do not contain causes that could not reliably be determined by the methods used.[1]

Section 4.1. Step 9: Drop VR country-years or mark as non-representative

Lozano and colleagues[9] describe the negative impact that low-completeness VR data could have on CoD modelling for GBD 2010. In settings where a data source does not capture all deaths in a population, the cause composition of deaths captured might be different from those that are not. For GBD 2019, VR location-years with completeness less than 50% were dropped, while location-yearswith completeness between 50% and 69% were marked as non-representative. In addition, any country-year with a number of deaths registered to major garbage codes greater than 50% of the deaths registered was dropped.[1]

Section 4.1. Step 10: Aggregate causes

The cause list is organised in a top-down hierarchical format containing four levels. Deaths are divided into three broad groupings (Level 1 causes): “communicable, maternal, neonatal, and nutritional diseases”; “non-communicable diseases”; and “injuries”. Within the Level 1 grouping of non-communicable diseases is the Level 2 cause “Diabetes and kidney diseases” which aggregates the Level 3 causes “Diabetes mellitus” and “Chronic kidney disease”. “Diabetes mellitus” aggregates the Level 4 causes “Diabetes mellitus type 1” and “Diabetes mellitus type 2”. “Chronic kidney disease” aggregates five Level 4 causes: “CKD due to diabetes type 1”, “CKD due to diabetes type 2”, “Hypertensive CKD”, “Glomerulonephritis CKD” and “Other CKD”. The mortality estimate for a parent cause in the hierarchy represents the sum of the mortality due to causes under that rubric. Included in the parent Level 3 cause estimate are deaths mapped directly to the parent and any Level 4 sub-causes.[1]

Section 4.1. Step 11: Remove shocks and HIV/AIDS maternal adjustments

For GBD 2019, CODEm models use an HIV/AIDS- and shock-free envelope. To be comparable, cause fractions must also be HIV/AIDS- and shock-free. Cause fractions were uploaded to the CoD database as the number of deaths due to the cause over an adjusted sample in which the number of deaths due to “HIV/AIDS”, “conflict and terrorism”, “executions and police conflict”, and “exposure to forces of nature” were removed.[1]

Section 4.1. Step 12: Apply noise-reduction algorithms

To deal with problems of zero counts in VR, VA, or sibling histories for a given age group in a given year, we use a Bayesian noise-reduction algorithm. For this algorithm, we assume a normal prior and a normal data likelihood. We estimate the normal prior for a given country-series of data by running a Poisson regression to estimate the number of deaths due to each respective cause and sex with dummy variables for age and year. With two exceptions, these regressions are sex-, cause-, and country-specific, so borrowing strength over age and year is only within a given data type, country, cause, and sex. Formula and greater detail are offered in the Capstone Appendix. The first exception is that country-years with populations under 1 million are pooled with the regional data to prevent over-dispersion and provide a stronger signal. The second is that handling of VA data diverges from the above description in two ways. First, all data for a given super-region are pooled together and a study dummy variable is added, allowing for different studies and surveillance sites to borrow strength from one another within a super-region. Second, unless the data are part of a time series (eg, the Matlab Health and Demographic Surveillance System), the regression has no year component.[1]

Section 4.1. Step 13: Identify outliers in the Cause of Death database

Death rates for different CoD generally have a stable age pattern. In large populations, these patterns will not change very rapidly over time. We can assume a relatively stable pattern in death rates for all causes except for some epidemic diseases and specific types of injuries. Rare causes in large populations and prevalent causes in small populations usually have stochastic patterns. To correct for these stochastic patterns, we implemented a noise-reduction process, explained in Step 12.[1]

In VR data, we infrequently find one or more datapoints for specific geography/age/sex/year combinations that lie very far from the stable pattern of death rates. In these situations, the model usually ignores the datapoint(s). If the model fails to ignore these data, dramatic jumps or drops can occur in the death rates. When no logical explanation exists for variation in the death rates to this degree, we regard the datapoint(s) as outlier(s). The selection of datapoints to regard as outliers occurs after data have been prepped for modelling, as well as during preliminary reviews of the models.[1]

In non-VR sources, data-collection methods and data quality can vary widely from source to source. Where datapoints in each age-sex-geography-year are very sparse, extreme datapoints can have a bad effect on regional estimation. In these situations, we investigate the study's methods and consider lower-quality datapoints as outliers.[1]

Identifying outliers in the CoD data occurs prior to finalisation of models for each cause. We do not automate the selection of outliers but investigate the source of the offending data as well as reviewing other data sources for the same cause, geography, and year. Ultimately, outliers are identified based on the judgement of the modeller and senior faculty. Outlier decisions are reversible and may be revisited.[1]

Section 4.2. Data star rating for the quality of VR

GBD estimates are most accurate when computed with a full time series of complete VR with a low percentage of garbage codes. Even countries with the highest-quality mortality registration systems continue to have major problems related to ill-defined causes of death. To deal with the inadequacies of vital registration, GBD developed a 5-star rating system to characterise quality of death reporting in terms of the fraction of deaths accurately certified. Countries improve in the star rating as they increase availability, completeness, and detail oftheir mortality data and reduce the percentage of deaths coded to ill-defined garbage codes or highly aggregated causes. Location- and year-specific information on completeness and data quality are listed in Capstone Appendix Figures 2 (Vital Registration and Verbal Autopsy data availability by country, 1980−2018) and Figure 4 (Percentage of vital registration deaths assigned to major garbage codes for all ages and sexes by country, 1980–2018).[1]

We assign "star" ratings to rate the quality of data for any given location-year. The inputs that determine this star rating are the percentage of total deaths determined to be major garbage codes (such as All, Ill-defined), and the level of completeness in the dataset. Causes such as "injuries" or "cancer" will also be included in the major garbage percentage because this percentage includes use of highly aggregated causes. These three values were used to create a "percent well-certified" value between 0 and 1, determined as:

Percent well certified = Completeness x (1 – Percent major garbage)

The mapping of percent well certified to star rating is as followed:

- 5 stars if percent of data well certified equaled or exceeded 85%
- 4 stars for 65% to less than 85%
- 3 stars for 35% to less than 65%
- 2 stars for 10% to less than 35%
- 1 star for greater than 0% to less than 10%

- 0 stars for 0% (no verbal autopsy or vital registration data were available over the period from 1980 to 2019)

Once percent well-certified is calculated for each location-year of VR and each VA study-year, we then combine these into one measurement for each five-year time interval and the full time series 1980–2019. For each five-year time interval, we assign the star level corresponding to that of the year with highest rating within the interval. Then for 1980–2019, we take the average of the maximum percentages well-certified for the seven five-year time intervals. Any five-year time interval in which no data were available were given a percent well-certified value of zero.

The number of countries at each star level over the over the full time series for all countries and the Americas' countries:

Number of countries at each star level

Star level	All countries	The Americas' countries
5 stars	30	7
4 stars	43	19
3 stars	30	10
2 stars	29	1
1 star	43	2
0 stars	29	0

Appendix Figure 3 presents the average star rating for locations for the period 2010–2018.

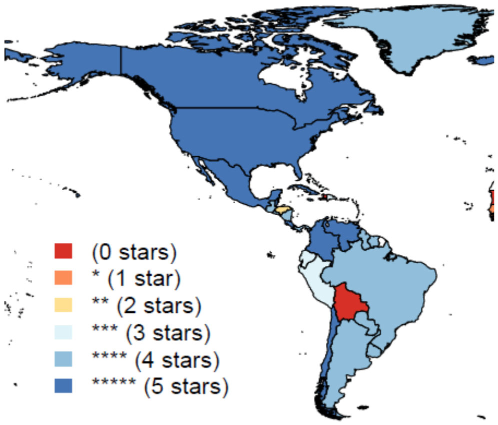

Appendix Figure 3. Classification of national vital registration and verbal autopsy data 2010–2018

The GBD 2019 Diseases and Injuries Capstone Appendix Figures 3 and 4 provide details of vital registration type and completeness, and percentage of recorded deaths whose cause was identified as a major (level 1 or 2) garbage code for each of the countries analysed.[1]

The Table S1 shows the fatal source counts for diabetes in the Americas, by location.

Table S1. Fatal source counts for diabetes by country in the Americas

Country	Source count
Argentina	37
Chile	36
Uruguay	35
Canada	36
United States of America	109
Antigua and Barbuda	31
Bahamas	27
Barbados	30
Belize	34
Cuba	37
Dominica	36
Dominican Republic	32
Grenada	28
Guyana	28
Jamaica	25
Saint Lucia	31
Saint Vincent and the Grenadines	29
Suriname	32
Trinidad and Tobago	33
Ecuador	38
Peru	30
Colombia	36
Costa Rica	37
El Salvador	28
Guatemala	35
Honduras	5
Mexico	38
Nicaragua	29
Panama	31
Venezuela	31
Brazil	38
Paraguay	35
Bermuda	35
Greenland	21

Saint Kitts and Nevis	35
Virgin Islands	22

Section 5. Cause of death modelling methods

Section 5.1. CODEm

Cause of Death Ensemble modelling (CODEm) is the framework used to model most cause-specific death rates in the GBD.[10] It relies on four key components:

First, all available data are identified and gathered to be used in the modelling process. Although the data may vary in quality, they all contain some signal of the true epidemiological process. Second, a diverse set of plausible models are developed to capture well-documented associations in the estimates. Using a wide variety of individual models to create an ensemble predictive model has been shown to outperform techniques using only a single model both in CoD estimation[11] and in more general prediction applications.[12] Third, the out-of-sample predictive validity is assessed for all individual models, which are then ranked for use in the ensemble modelling stage. Finally, differently weighted combinations of individual models are evaluated to select the ensemble model with the highest out-of-sample predictive validity.

For some causes, evidence exists that the relationship between covariates and death rates might differ between children and adults. Separate models are therefore run for different age ranges, when applicable. Specifically, in the case of these analyses, deaths under age 15 are assumed to be due to type 1 diabetes, and above that age, due to type 2 diabetes. Additionally, separate models are developed for countries with extensive, complete, and representative VR for every cause to ensure that uncertainty can better reflect the more complete data in these locations.

Because many factors may co-vary with any given CoD, a range of plausible statistical models are developed for each cause. In the CODEm framework, four families of statistical models are used: linear mixed effects regression (LMER) models of the natural log of the cause-specific death rate, LMER models of the logit of the cause fraction, spatiotemporal Gaussian process regression (ST-GPR) models of the natural logarithm of the cause-specific death rate, and ST-GPR models of the logit of the cause fraction. The component models are weighted based on their predictive validity rank to determine their contribution to the ensemble estimate. A set of ensemble models is then created by using the weights.

The performance of all models (individual and ensemble) is evaluated by means of out-of-sample predictive validity tests. 30% of the data are randomly excluded from the initial model fits. Individual model fits are evaluated and ranked by using half of the excluded data (15% of the total), then used to construct the ensembles based on their performance. These ensembles are tested by using the predictive validity metrics on the remaining 15% of the data, and the ensemble with the best performance in out-of-sample trend and root mean square error is chosen as the final model. Greater details of this process, including development of the model pool, data variance estimation, the testing of the model pool on a 15% sample, and ensemble development and testing are given in the Capstone Appendix.

Once a weighting scheme has been chosen, 1000 draws are created for the final ensemble, with the number of draws contributed by each model proportional to its weight. The mean of the draws is then used as the final estimate for the CODEm process, and a 95% uncertainty interval (UI) is created from the 0·025 and 0·975 quantiles of the draws. The validity of the UI can be checked via its coverage of the out-of-sample data; ideally, the 95% UI would capture 95% of these data. Higher coverage suggests that the UIs are too large, and lower coverage suggests overfitting.

Section 5.2 Causes modelled outside of CODEm

CODEm is used to model both types of diabetes as well as CKD. However, the distribution of CKD deaths due to diabetes into the separate categories type 1 and type 2 diabetes is performed with DisMod-MR 2.1, which permits adjustment based on the prevalence of each type. Until GBD 2010, non-fatal estimates such as prevalence were based on a single data source on prevalence, incidence, remission, or a mortality risk selected by the researcher as most relevant to a particular location and time. Beginning with GBD 2010, a more ambitious goal was set: to evaluate all available information on a disease that passes a minimum quality standard. That required a different analytical tool that would be able to pool disparate information presented in varying age groupings and from data sources by using different methods. The DisMod-MR tool evaluates and pools all available data, adjusting data for systematic bias associated with methods that varied from the reference, and produces estimates with UIs by world regions.

Flow of data and settings is organised in an analytical cascade across different levels. The sequence of estimation occurred at five levels: global, super-region, region, country, and, where applicable, subnational locations. The super-region priors were generated at the global level with mixed-effects, non-linear regression by using all available data; the super-region fit, in turn, informed the region fit, and so on down the cascade. The DisMod-MR 2.1 "wrapper" gives analysts the choice to branch the cascade in terms of time and sex at different levels depending on data density. The default used in most models was to branch by sex after the global fit but to retain all years of data until the lowest level in the cascade. Greater detail on DisMod-MR 2.1 is available in the Capstone Appendix.

Section 5.3. CoD Correct

The CoD models are cause-specific. As such, there is no guarantee that the sum of these models will equal the results of the all-cause mortality estimates or that model results of child causes add up to the parent model results. The CoDCorrect process is used to make the CoD and all-cause mortality estimates internally consistent. The CoDCorrect process starts by rescaling the Level 1 causes to match the all-cause mortality estimates. Level 2 causes are then rescaled to their corrected parent causes. This process continues until all levels of the hierarchy have been rescaled.

Section 5.4. Years of life lost calculation

Years of life lost (YLLs) owing to premature mortality were computed for 1082 locations and 39 years. First, we used the lowest observed age-specific mortality rates by location and sex across all estimation years from locations with total populations greater than 5 million in 2016 to establish a theoretical minimum risk reference life table.

The YLL is a metric that is computed by multiplying the number of estimated deaths by the standard life expectancy at age of death. The metric therefore highlights premature deaths by applying a larger weight to deaths that occur in younger age groups. We propagated uncertainty from CoDCorrected deaths for all demographics. The core equation can be written as follows:

$$YLL = \sum_{c=1,a=0,s=1}^{\infty} d_{cas} e_a$$

Section 5.5 GBD world population age standard

Age-standardised populations in the GBD were calculated by using the GBD world population age standard. We used the non-weighted mean of 2019 age-specific proportional distributions from the GBD 2019 population estimates for all national locations with a population greater than 5 million people in 2019 to generate an updated standard population age structure.[2]

Section 5.6 Statistical analyses

GBD analyses were conducted with Python version 3.6.2, Stata version 13, and R version 3.5.0.

Section 6. Specific CoD modelling descriptions

The following text, flowcharts, and tables, as presented in the Capstone Appendix, describe details of modelling for diabetes, overall and by type, and CKD, overall and that due to type 1 and type 2 diabetes.

Section 6.1 Diabetes mellitus

Diabetes mortality was estimated for overall diabetes, diabetes type 1, and diabetes type 2 in GBD 2019.

The following ICD codes were mapped to diabetes[1]:

Disease	ICD-9	ICD-10
Diabetes mellitus	250.00-250.99, 775.1	E10-E10.11, E10.3-E11.1, E11.3-E12.1, E12.3-E13.11, E13.3-E14.1, E14.3-E14.9, P70.2
Diabetes mellitus type 1	250-250.0, 250.01, 250.03-250.1, 250.11, 250.13-250.2, 250.21, 250.23-250.3, 250.31, 250.33-250.39, 250.5, 250.51, 250.53-250.6, 250.61, 250.63-250.7, 250.71, 250.73-250.8, 250.81, 250.83-250.9, 250.91, 250.93-250.99, 775.1	E10-E10.11, E10.3-E10.9, P70.2
Diabetes mellitus	250.00, 250.02, 250.10, 250.12,	E11-E11.1, E11.3-E11.9

type 2	250.20, 250.22, 250.30, 250.32, 250.50, 250.52, 250.60, 250.62, 250.70, 250.72, 250.80, 250.82, 250.90, 250.92

The GBD map the ICD codes to the GBD cause list. In its analysis, the GBD does not use the details of coding in modelling (eg, .0x–.9x) of codes 250 and E10-14.

Section 6.1.1. Overall diabetes mellitus

Flowchart

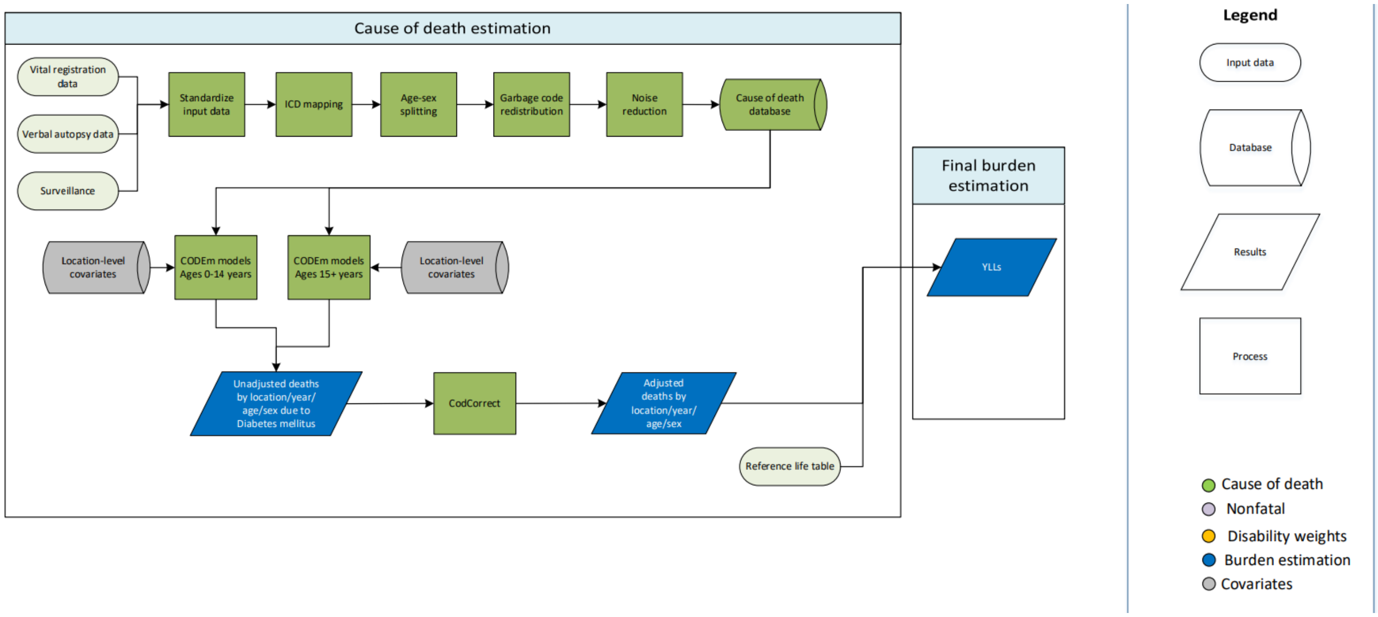

Section 6.1.1.1. Input data

Overall diabetes mellitus mortality was estimated using deaths directly attributed to diabetes mellitus. We used verbal autopsy and vital registration data as inputs into the model.

Verbal autopsy data: We outliered datapoints from sources where there were zero deaths estimated in an age group as this was not realistic for deaths due to diabetes and we determined that these data sources were unreliable.

Vital registration data: We outliered all data from the India Medical Certification of Cause of Death report since the source of the data was unreliable according to expert opinion. We also outliered ICD9BTL datapoints that were inconsistent with the rest of the data series and created unlikely time trends.

Section 6.1.1.2. Modelling strategy

The Cause of Death Ensemble model (CODEm) was used for deaths due to diabetes mellitus estimation. In the overall diabetes mellitus model, we used two models to estimate overall diabetes deaths with different age restrictions. This is because deaths in younger age groups are almost exclusively due to

type 1 diabetes, while deaths in older ages are primarily due to type 2 diabetes. This allowed us to select predictive covariates that are specific to the pathophysiology of diabetes type 1 and type 2. We set the younger age model from 0-14 years and the older age model from 15-95+ years. We determined the age threshold based on evidence of the onset age of diabetes type 2 occurring at younger ages.

Section 6.1.1.3. Covariate selection

The following table lists the covariates included in the model. This requires that the covariate selected for the model must have the directional relationship with diabetes mellitus deaths. In GBD 2019, we made two updates. First, we changed four covariates to reflect the most current covariate available, proportion underweight to age-standardised underweight (weight-for-age) summary exposure variable, proportion stunting to age-standardised stunting (height-for-age) summary exposure variable, energy-adjusted grams of fruits to age- and sex-specific summary exposure variable for low fruit, and energy-adjusted grams of vegetables to age- and sex-specific summary exposure variable for low vegetables.

Second, we selected a direction on covariates for which we did not set a direction in previous GBD. We determined the direction based on the strength of the evidence.

Model	Level	Covariate	Direction
0-14 years	1	Healthcare Access and Quality Index	-
	3	Education years per capita	-
	2	Age-standardised fertility rate	+
	2	Latitude	+
	2	Age-standardised underweight (weight-for-age) summary exposure variable	-
	2	Percentage of births occurring in women >35 years old	+
	2	Percentage of births occurring in women >40 years old	+
	3	Socio-demographic Index	-
	2	Age-standardised stunting (height-for-age) summary exposure variable	-
	2	Mean birthweight	-
15 + model	1	Age-standardised mean fasting plasma glucose (mmol/L)	+
	1	Age-standardised prevalence of diabetes	+
	3	Education years per capita	-
	3	Lag-distributed income per capita	+
	1	Mean BMI	+
	2	Mean cholesterol	+
	2	Mean systolic blood pressure	+
	1	Prevalence of obesity	+
	2	Age- and sex-specific summary exposure variable for low fruit	-

	2	Energy-adjusted grams of sugar	+
	2	Age- and sex-specific summary exposure variable for low vegetables	-
	3	Healthcare Access and Quality Index	-
	2	Age- and sex-specific summary exposure variable for alcohol use	+

Section 6.1.1.4. Covariate influences

The following plots show the influence of each covariate on the four CODEm models (male global, male data-rich, female global, and female data-rich). A positive standardised beta (to the right) means that the covariate was associated with increased death. A negative standardised beta (to the left) means the covariate was associated with decreased death.

Data rich | **Global**

Female

0-14

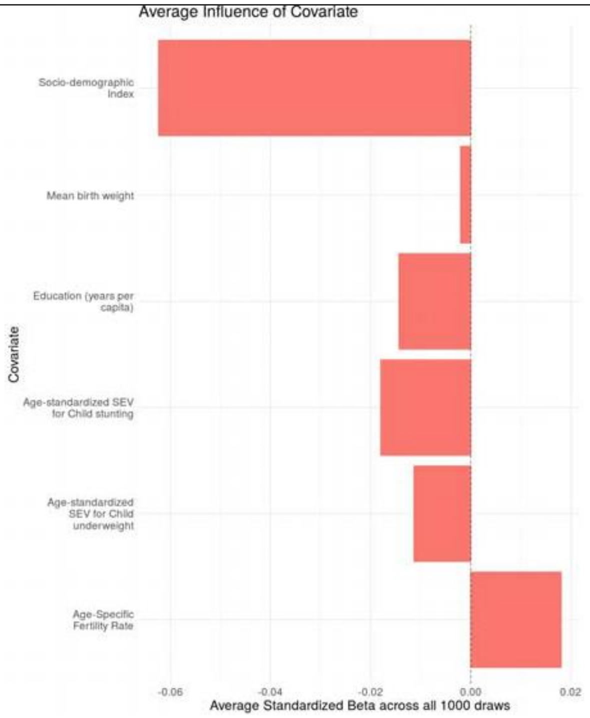

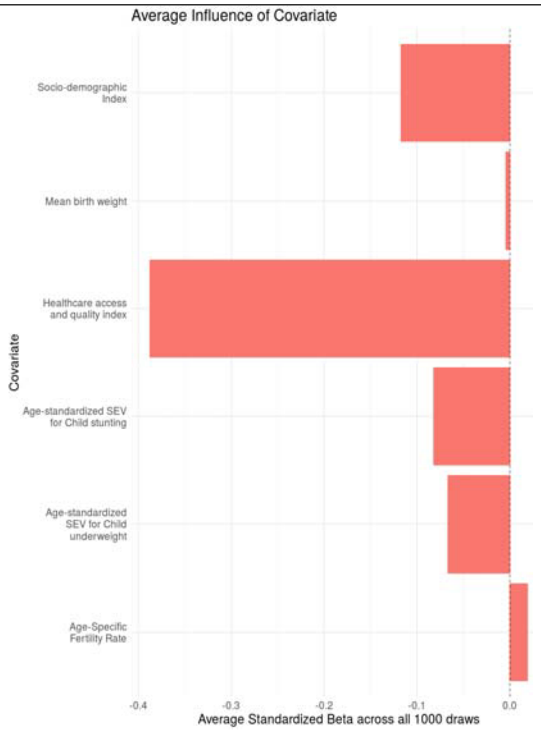

<table>
<tr><th></th><th>Data rich</th><th>Global</th></tr>
<tr><td>Male
0 -14
model</td><td>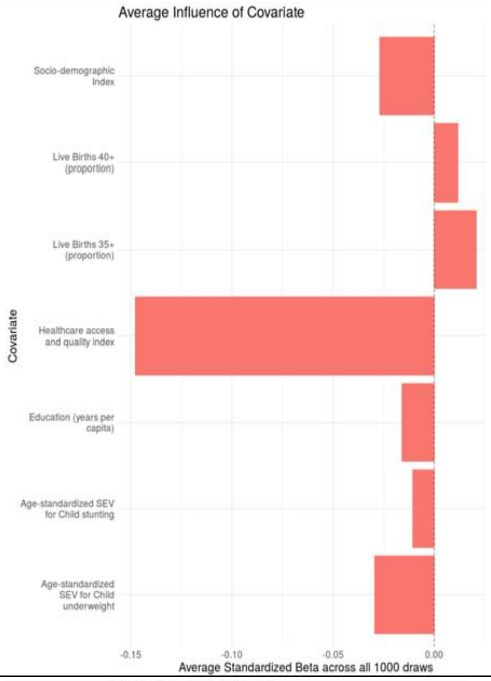
</td><td>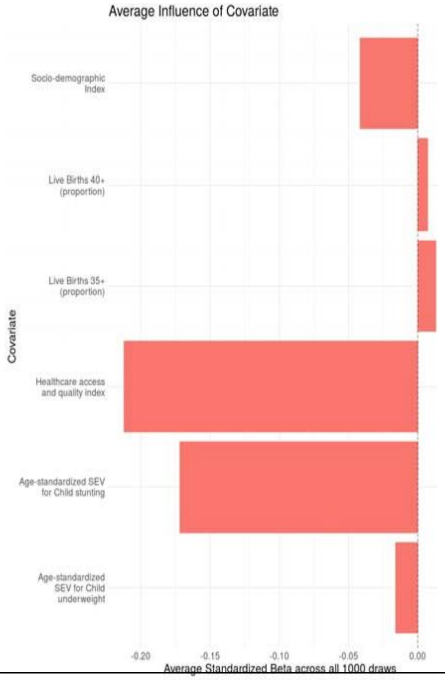
</td></tr>
<tr><td>Female 15+
model</td><td>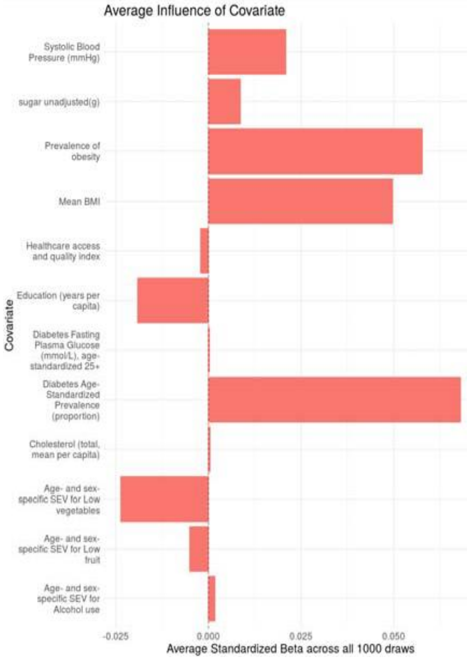
</td><td>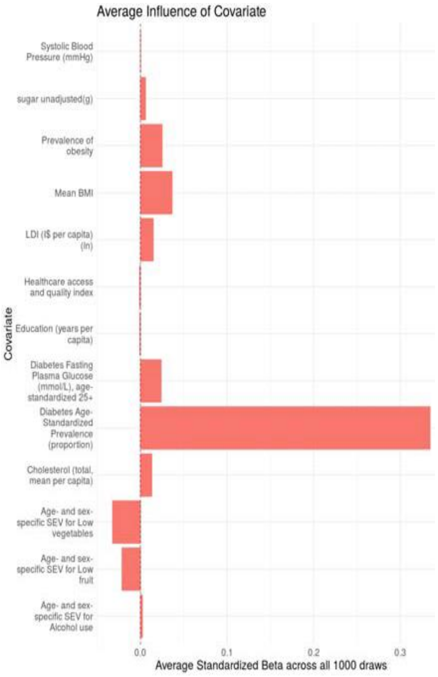
</td></tr>
</table>

<table>
<tr><th></th><th>Data rich</th><th>Global</th></tr>
<tr><td>Male15+
model</td><td>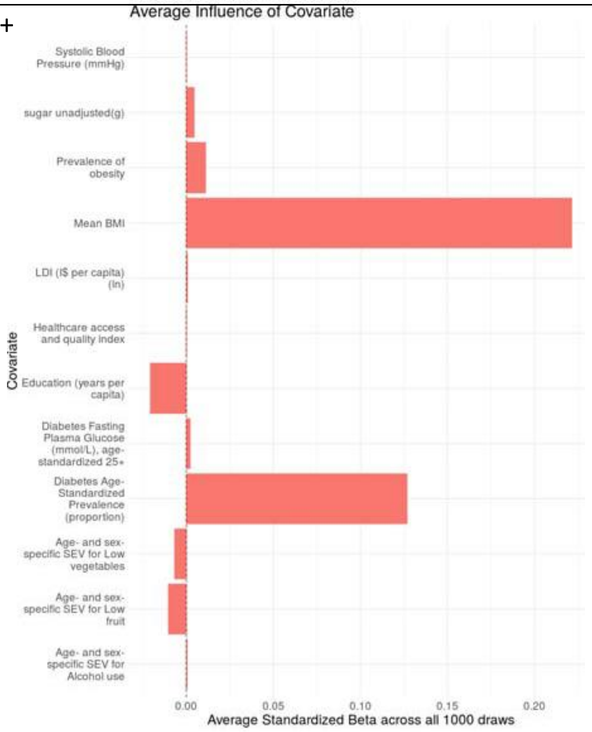
</td><td>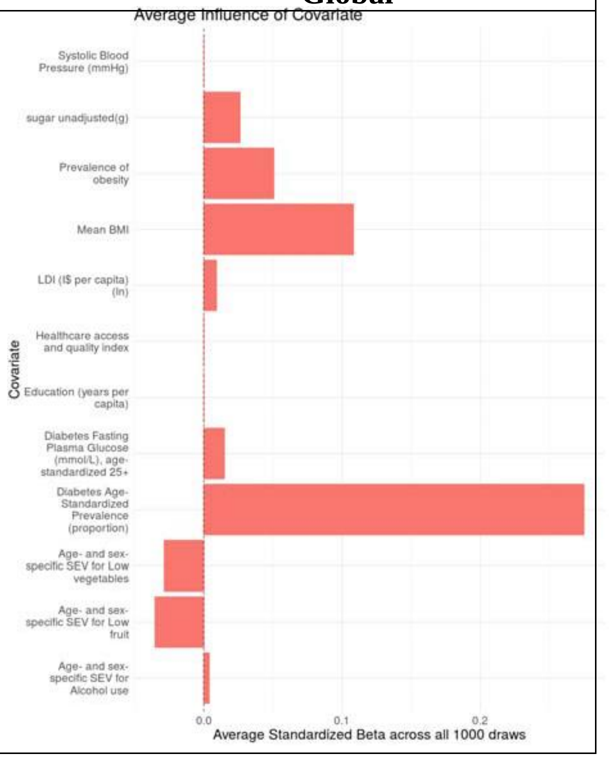
</td></tr>
</table>

Section 6.1.2. Diabetes mellitus type 1 and type 2

Flowchart

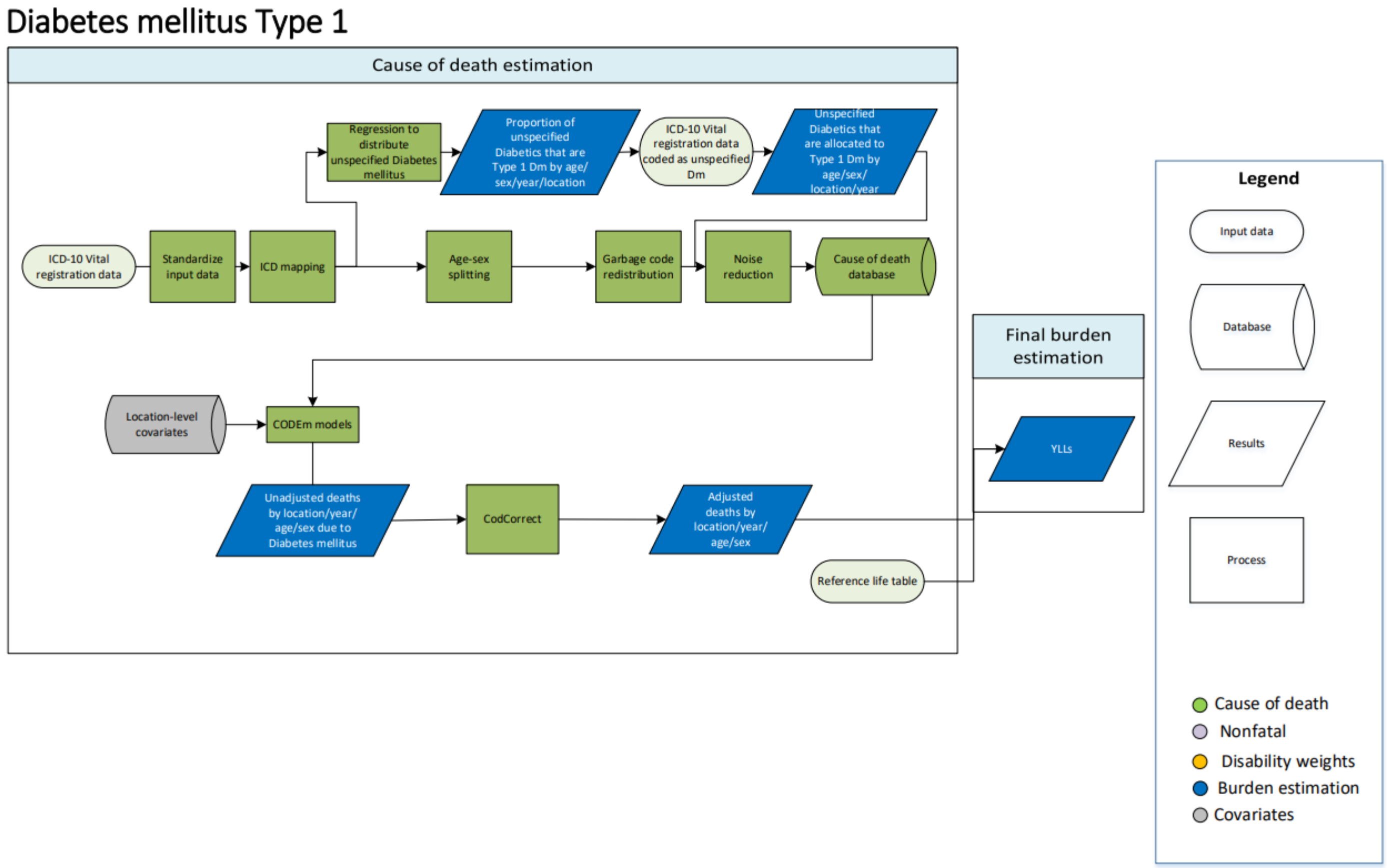

Diabetes mellitus Type 2

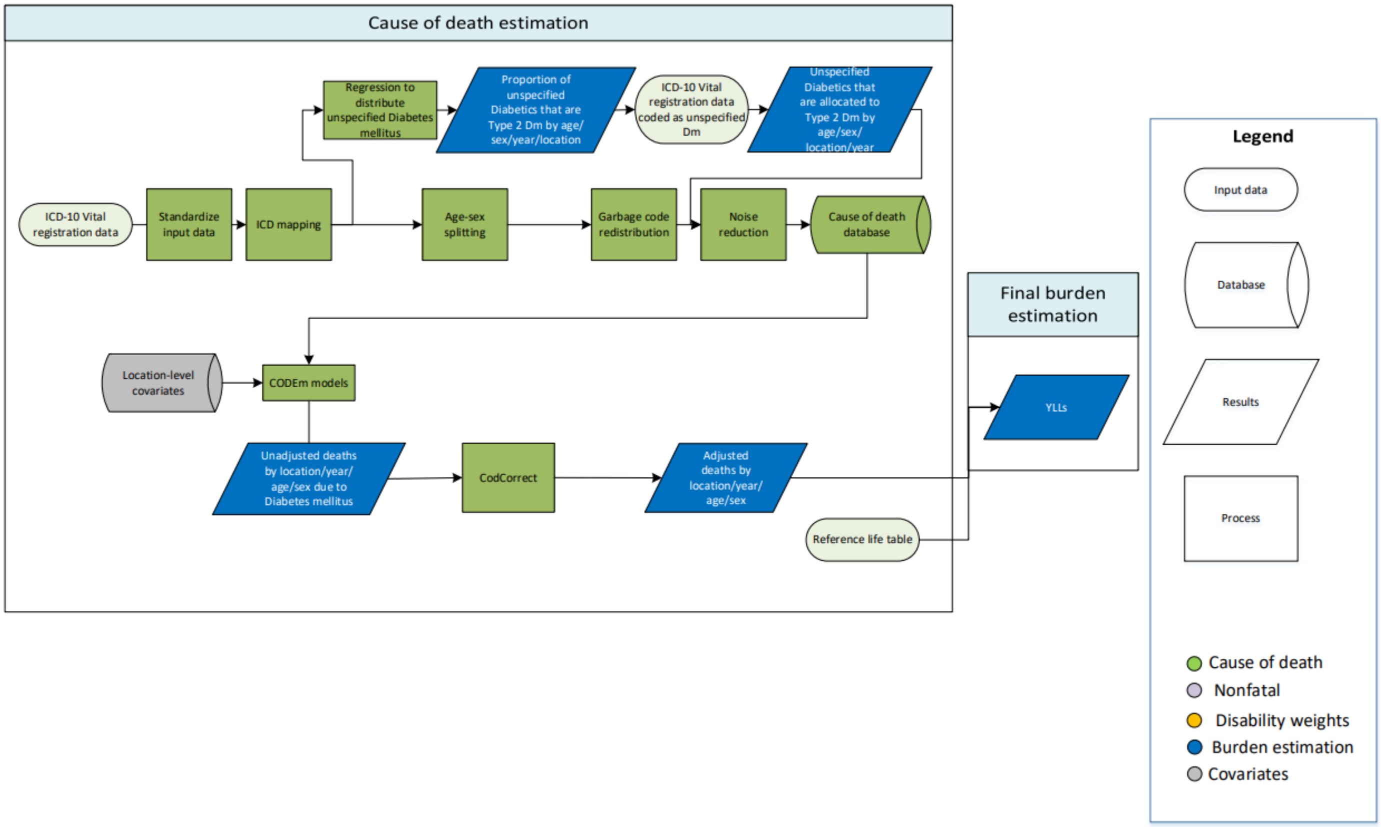

Section 6.1.2.1. Input data

Type-specific diabetes mellitus mortality was estimated using deaths from vital registration sources in ICD-10 codes only. Diabetes type-specific information was not available in ICD-9 codes or deaths determined by verbal autopsy.

Section 6.1.2.2. Modelling strategy

The Cause of Death Ensemble model (CODEm) was used for deaths due to diabetes mellitus estimation.

Deaths in younger age groups are almost exclusively due to type 1 diabetes, while deaths in older ages are primarily due to type 2 diabetes. To account for this age pattern, we set the age range of the diabetes type 1 model to 0–95+ years and the age range of the diabetes type 2 model to 15–95+ years. We used the same covariates in the diabetes type 1 model and diabetes type 2 model as the 0–14 year and 15–95+ year in the overall diabetes models, respectively.

There were two unique data manipulation steps that occurred to prepare the data as part of the modelling process.

1. We assumed that all deaths <15 years were due to type 1 regardless of the ICD-10 code assigned to the death. We imposed 100% attribution of diabetes mellitus deaths in <15 years to type 1 diabetes mellitus.
2. ICD-10 diabetes data were reported as type 1, type 2, or unspecified. We developed a regression to estimate the fraction of unspecified diabetes mellitus that was type 1 and type 2. We only used data from 703 country-years to inform the regression. This is because these country-years had more than 50% of the deaths typed to type 1 or type 2 AND at least 70% of type-specific deaths in people >25 years were coded to type 2. Since there was a separate regression to estimate the proportion of type 1 diabetes mellitus and type 2 diabetes mellitus, we scaled the predicted proportions to one. These scaled proportions were then applied to number of deaths coded to unspecified diabetes in each location, year, sex where ICD-10 data were reported.

<u>Regression equations</u>

Type 1:

$$logit\left(\frac{number\ type\ 1\ DM}{number\ total\ DM}\right) \sim logit\left(\frac{number\ unspecified\ DM}{number\ total\ DM}\right) + \beta_1 age\ group + \beta_2 age\text{-}st\ prev\ obesity * age\ group + age\text{-}st\ prev\ obesity$$

Type 2:

$$logit\left(\frac{number\ type\ 2\ DM}{number\ total\ DM}\right) \sim logit\left(\frac{number\ unspecified\ DM}{number\ total\ DM}\right) + \beta_1 age\ group + \beta_2 age\text{-}st\ prev\ obesity * age\ group + age\text{-}st\ prev\ obesity$$

Section 6.1.2.3. Covariate selection

The following are the covariates included in the model. We selected the same covariates for the type 1 diabetes model as the 0–14 year diabetes model and the type 2 diabetes model as the 15–95+ year diabetes model.

Model	Level	Covariate	Direction
Type 1	1	Healthcare Access and Quality Index	-
	3	Education years per capita	-
	2	Age-standardised fertility rate	+
	2	Latitude	+
	2	Age-standardised underweight (weight-for-<br>age) summary exposure variable	-
	2	Percentage of births occurring in women >35 years old	+
	2	Percentage of births occurring in women >40 years old	+
	3	Socio-demographic Index	-
	2	Age-standardised stunting (height-for-age)<br>summary exposure variable	-
	2	Mean birthweight	-
Type 2	1	Age-standardised mean fasting plasma glucose (mmol/L)	+
	1	Age-standardised prevalence of diabetes	+
	3	Education years per capita	-
	3	Lag-distributed income per capita	+
	1	Mean BMI	+
	2	Mean cholesterol	+
	2	Mean systolic blood pressure	+
	1	Prevalence of obesity	+
	2	Age- and sex-specific summary exposure variable for low fruit	-
	2	Energy-adjusted grams of sugar	+
	2	Age- and sex-specific summary exposure variable for low vegetables	-
	3	Healthcare Access and Quality Index	-
	2	Age- and sex-specific summary exposure variable for alcohol use	+

Section 6.1.2.4. Covariate influences:

The following plots show the influence of each covariate on the four CODEm models (male global, male data-rich, female global, and female data-rich). A positive standardised beta (to the right) means that the

covariate was associated with increased death. A negative standardised beta (to the left) means the covariate was associated with decreased death.

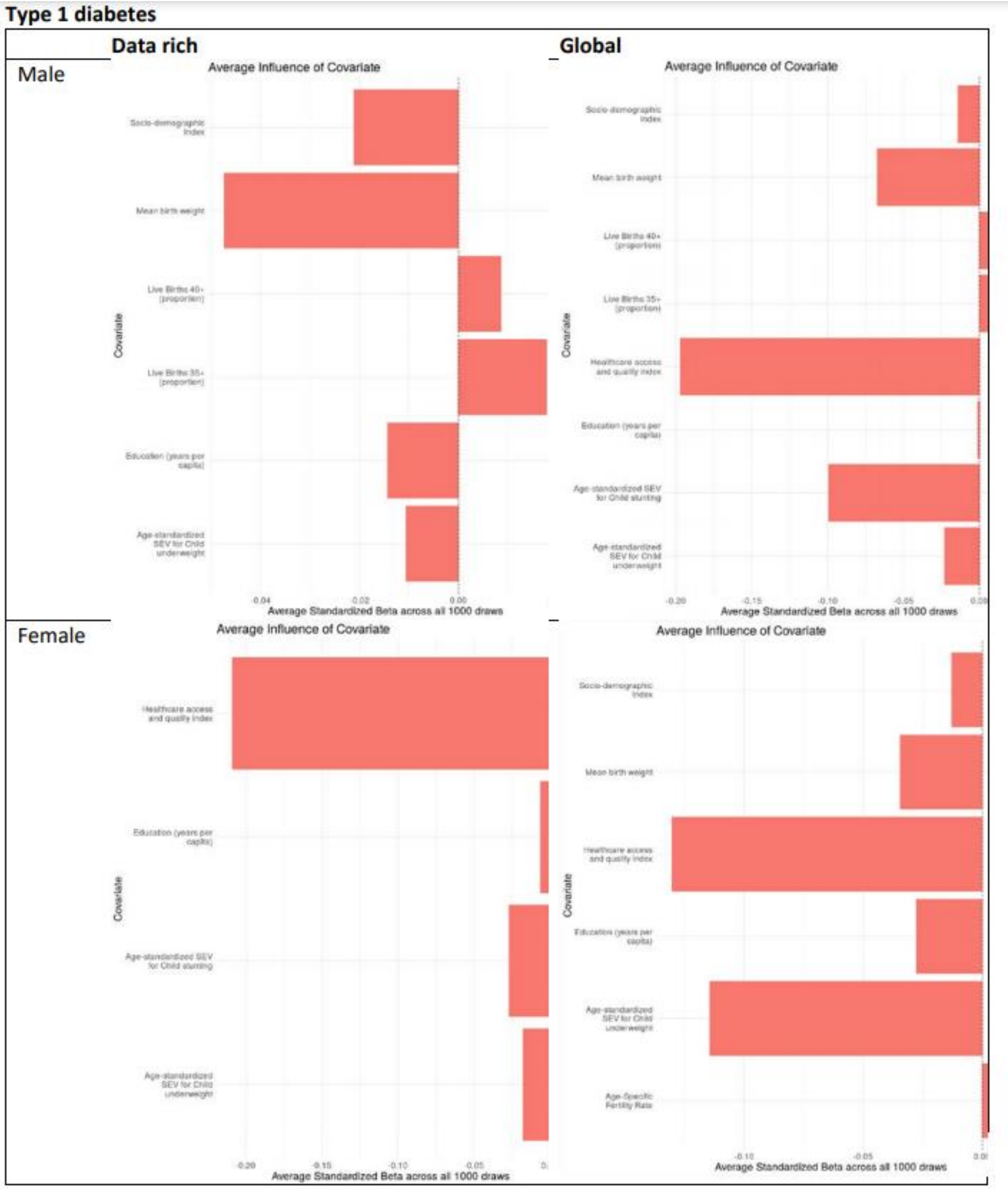

Type 2 diabetes

	Data rich	Global
Male	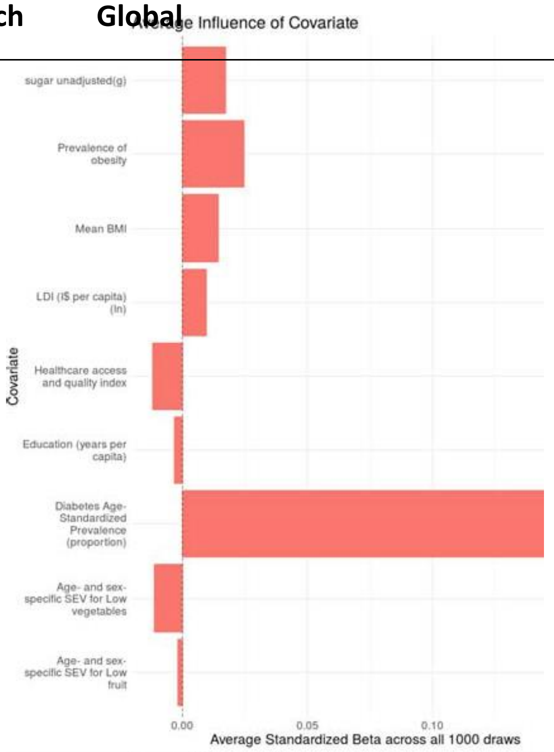	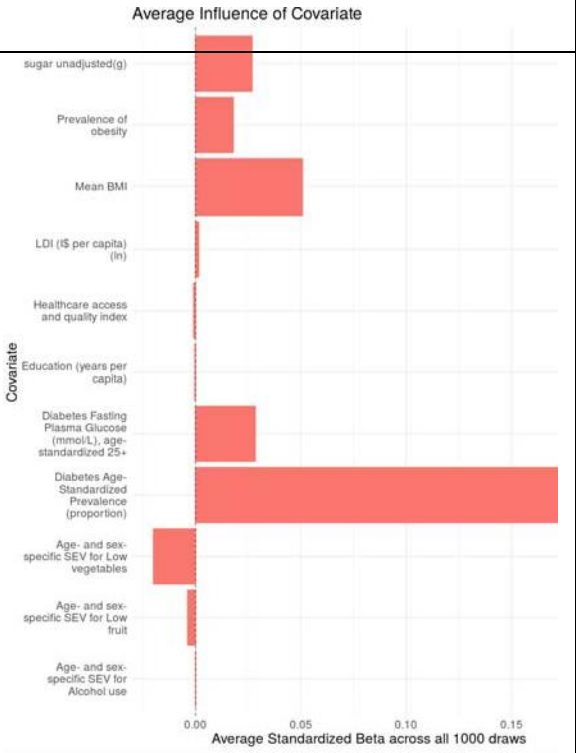
Female	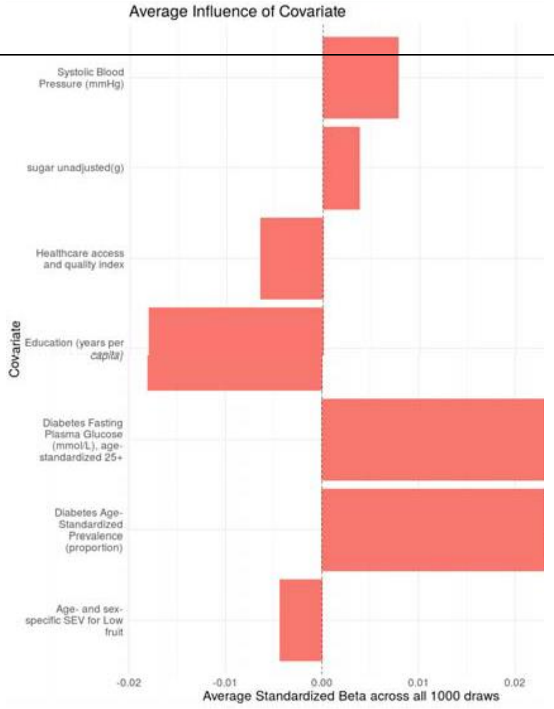	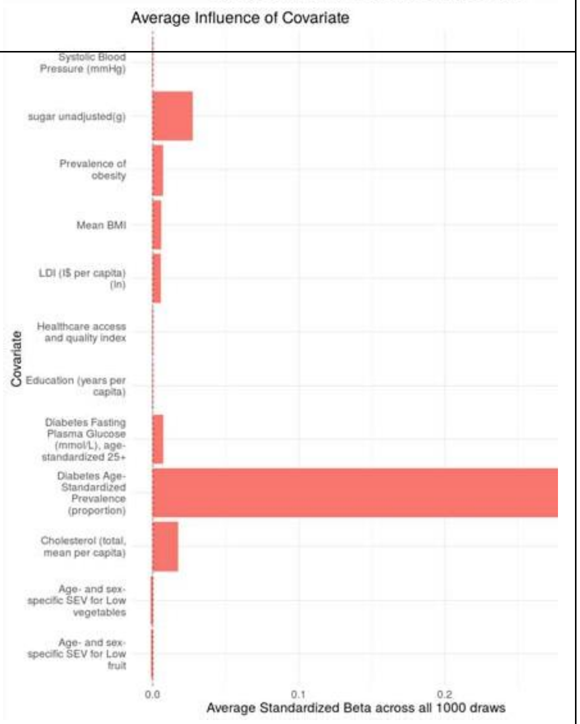

Section 6.2. Chronic kidney disease

Flowchart

Chronic Kidney Disease

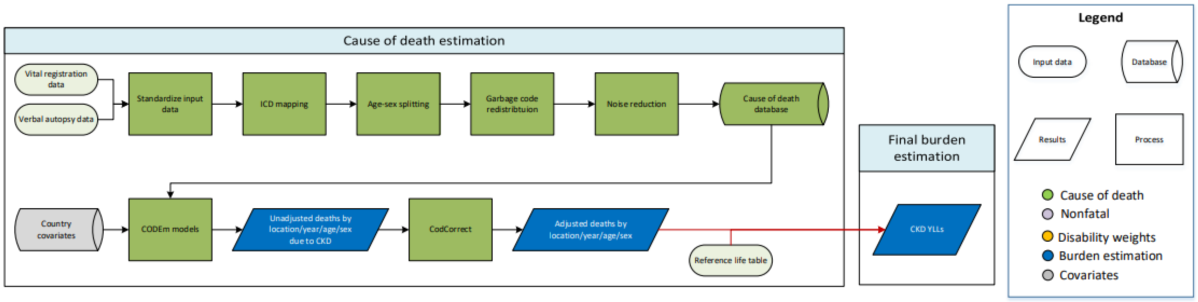

Section 6.2.1. Input data

Vital registration and verbal autopsy data were used to model mortality due to chronic kidney disease. Data were standardised and mapped according to the GBD causes of death ICD mapping method. These data were then age-sex split, and appropriate redistribution of garbage code data was performed. Datapoints that violated well-established age or time trends or that resulted in extremely high or low cause fractions were marked as outliers and excluded.

Section 6.2.2. Modelling strategy

The estimation strategy used for fatal chronic kidney disease is largely similar to methods used in GBD 2017. A standard CODEm model with location-level covariates was used to model deaths due to chronic kidney disease.

The full list of covariates used in the GBD 2019 model is displayed below.

Level	Covariate	Direction
1	Diabetes fasting plasma glucose (mmol/L)	+
	Diabetes age-standardised prevalence (proportion)	+
	Mean systolic blood pressure (mmHg)	+
	Mean BMI	+
	Healthcare access and quality index	–
2	Mean cholesterol	+
	Total Calories available per capita per day	+
	Red meat unadjusted (kcal per capita)	+
3	Socio-demographic Index	–
	Education (years per capita)	–
	LDI (I$ per capita)	–

Section 6.2.3. Covariate influences

The following plots show the influence of each covariate on the four CODEm models (male global, male data-rich, female global, and female data-rich). A positive standardised beta (to the right) means that the covariate was associated with increased death. A negative standardised beta (to the left) means the covariate was associated with decreased death.

Male Global

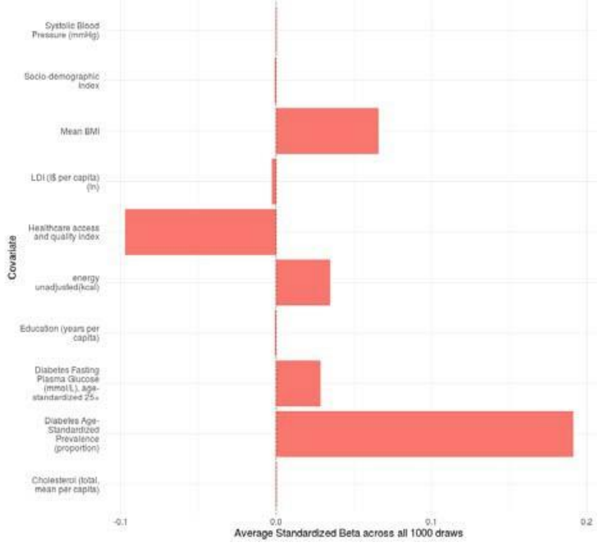

Male Data Rich

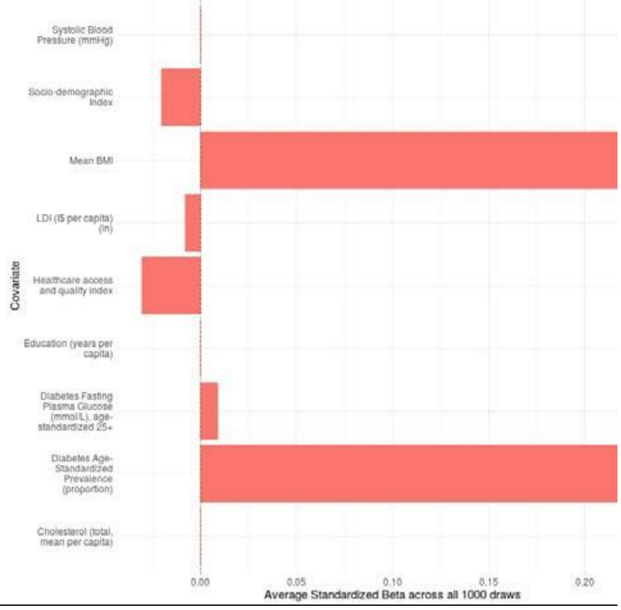

Female Global

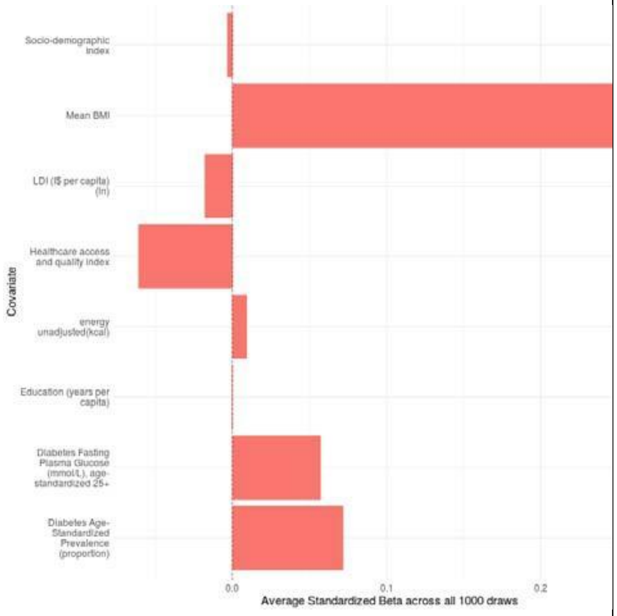

Female Data Rich

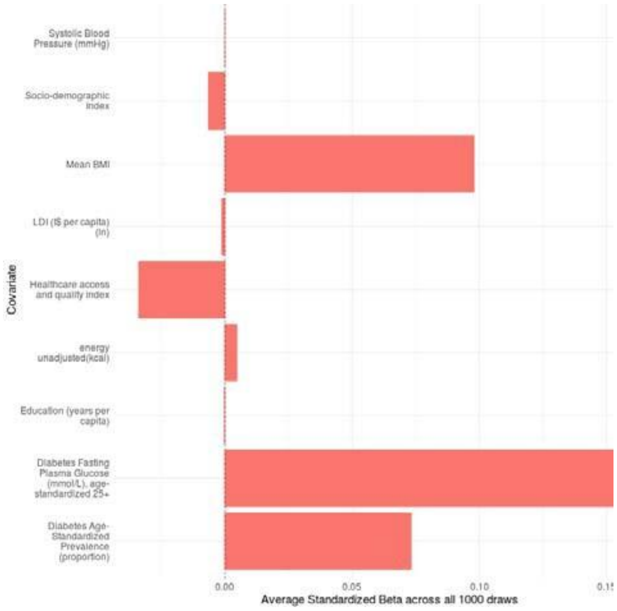

Section 6.2.4. Chronic kidney disease subtypes, including those due to type 1 and type 2 diabetes

Flowchart

Chronic Kidney Disease subtypes

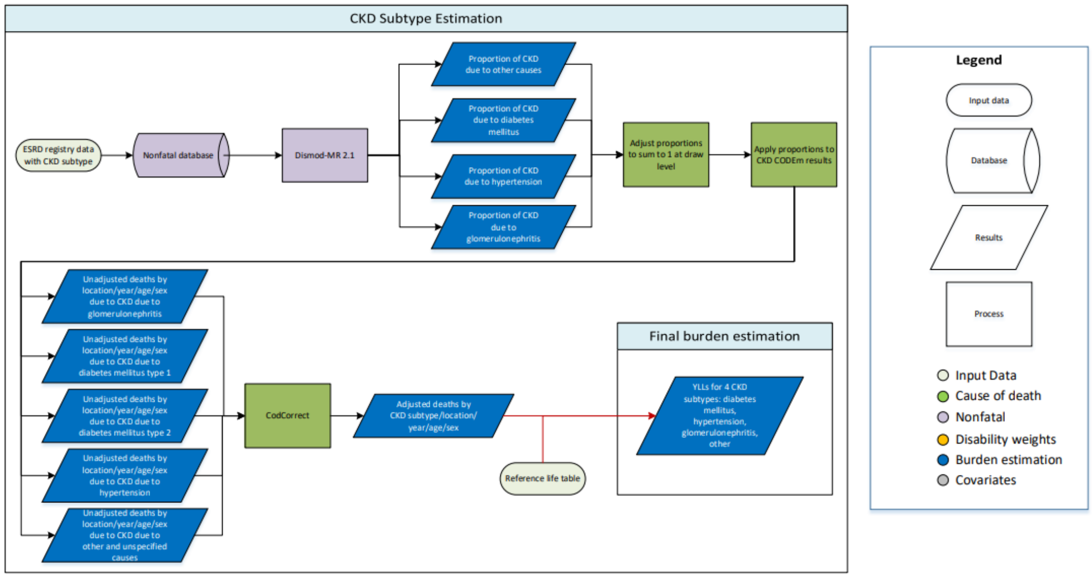

Section 6.2.4.1. Input data

We estimated deaths due to five subtypes of chronic kidney disease: diabetes mellitus type 1, diabetes mellitus type 2, hypertension, glomerulonephritis, and other causes.

The following codes were used to identify CKD due to diabetes:

Aetiology	ICD-9 Codes	ICD-10 Codes
Type 1 diabetes	250.41, 250.43	E10.2, E10.21, E10.22, E10.29
Type 2 diabetes	250.40, 250.42	E11.2, E11.21, E11.22, E11.29

Deaths due to congenital kidney anomalies (cystic kidney disease and reflux hydronephrosis) were included in the latter category. Data from end-stage renal disease registries were used to estimate proportion of CKD mortality attributable to each CKD subtype. Age-specific data on the proportion of ESRD by subtype was available from the USA, Australia, New Zealand, Nigeria, and Russia.

Vital registration (VR) data were excluded from subtype-specific estimates, as aetiology coding in VR sources was considered to be of highly variable quality between countries.

Section 6.2.4.2. Modelling strategy

We utilised data primarily from end-stage kidney registries that included CKD aetiologies to model CKD-death aetiology proportions.

Data for CKD due to overall diabetes were more widely available than data by type of diabetes. In order to make use of all available data, we modelled the proportion of CKD due to overall diabetes, diabetes type 1, and diabetes type 2. We ran DisMod-MR 2.1 models including diabetes prevalence and mean systolic blood pressure as country-level covariates to obtain estimates of proportions for

each subtype by location, year, age, and sex. Proportion of CKD due to diabetes type 1 and diabetes type 2 were then scaled to sum to the proportion of overall diabetes at the gender-, age-, and country-matched level. The results from all subtype-specific modelswere adjusted so that estimates across the subtypes equaled 1 at each of 1000 draws. These adjusted proportions were applied to the parent CKD CODEm model to obtain type-specific estimates of CKD mortality.

Section 7. Non-fatal outcome estimation

The GBD 2019 non-fatal estimation process describes the steps necessary to estimate incidence, prevalence, and years lived with disability (YLDs) for disease and injury sequelae in GBD 2019. Conceptually, the estimation effort is divided into eight major components: (1) compiling data sources through data identification and extraction; (2) data adjustment; (3) estimation of prevalence and incidence by cause and sequelae by using DisMod-MR 2.1 or alternative modelling strategies for selected cause groups; (4) estimation by impairment; (5) severity distributions; (6) incorporation of disability weights (DWs); (7) comorbidity adjustment; and (8) the estimation of YLDs by sequelae and causes. An overview of some of these steps is provided below. The methods appendix of the capstone Disease and Injuries[1] contains additional detail as well as details specific to each non-fatal disease, impairment, and injury, and their sequelae. Non-fatal modelling strategies vary significantly between causes.

Section 7.1. Estimation of prevalence and incidence by cause and sequelae by using DisMod-MR 2.1

The most extensively used estimation method is the Bayesian meta-regression method DisMod-MR 2.1. based on the underlying three-state model (susceptible, cases, dead). For diseases with a range of sequelae differentiated by severity, such as diabetes mellitus, DisMod-MR 2.1 was used to meta-analyse the data on overall prevalence with separate DisMod-MR 2.1 models of the proportions of cases with different severity levels or sequelae. Estimation occurred at the five levels of the GBD location hierarchy—global, super-regional, regional, national, and subnational—with results of each higher level providing guidance for the analysis at the lower geographical level, as described in the figure below.

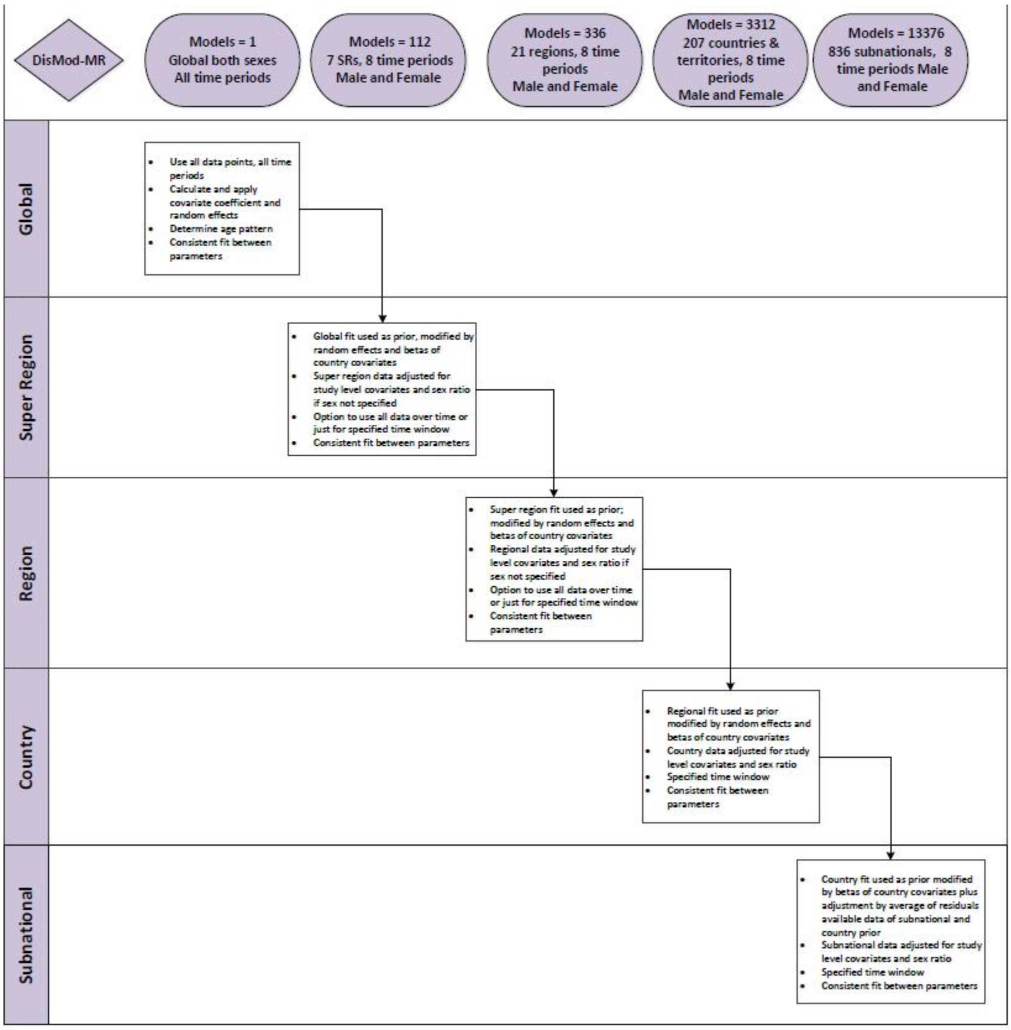

Appendix Figure 4. GBD 2019 DisMod-MR 2.1 analytical cascade

Section 7.1.2. Severity distribution

Sequelae were defined in terms of severity. In cases in which severity was related to a particular impairment, such as mild, moderate, and severe blindness due to diabetic retinopathy, the analysis was driven by impairment estimation methods. Severity levels for causes such as chronic kidney disease were modelled using DisMod-MR 2.1 or ST-GPR.

Section 7.1.3 Disability weights

To compute YLDs for a particular health outcome in a given population, the number of people living with that outcome is multiplied by a disability weight (DW) that represents the magnitude of health loss associated with the outcome. DWs are measured on a scale from 0 to 1; 0 implies a state equivalent to full health, and 1, a state equivalent to death.

Section 7.1.4. Comorbidity adjustment

The final stage in the estimation of YLDs is a micro-simulation, which adjusts for comorbidity, referred to as "COMO" (for comorbidity correction). For GBD 2019, we estimated the co-occurrence of different diseases by simulating 40,000 individuals in each location-age-sex-year combination as exposed to the independent probability of having any of the sequelae included in GBD 2019 based on disease prevalence. We tested the contribution of dependent and independent comorbidity in the USA MEPS data and found that independent comorbidity was the dominant factor even though well-known examples of dependent comorbidity exist, such as clustering of conditions like diabetes

and stroke. Age was the main predictor of comorbidity, such that age-specific micro-simulations accommodated most of the required comorbidity correction.

Section 7.1.5. YLD computation and uncertainty

We computed YLDs by sequela as prevalence multiplied by the DW for the health state associated with that sequela. The uncertainty ranges reported around YLDs incorporate uncertainty in prevalence and uncertainty in the DW. To do this, we take the 1000 samples of comorbidity-corrected YLDs and 1000 samples of the DW to generate 1000 samples of the YLD distribution. We assume no correlation in the uncertainty in prevalence and DWs. The 95% uncertainty interval is reported as the 25th and 975th values of the distribution.

Section 7.2. DALY computation and uncertainty

To estimate DALYs for GBD 2019, we started by estimating cause-specific mortality and non-fatal health loss. For each year for which YLDs have been estimated, we computed DALYs by adding YLLs and YLDs for each age-sex-location. Uncertainty in YLLs was assumed to be independent of uncertainty in YLDs. We calculated 1000 draws for DALYs by summing the first draw of the 1000 draws for YLLs and YLDs and then repeating for each subsequent draw. 95% UIs were computed by using the 25th and 975th ordered draw of the DALY uncertainty distribution. We calculated DALYs as the sum of YLLs and YLDs for each cause, location, age group, sex, and year. For more information, please refer to the following figure A.

Figure A. DALY burden estimation for GBD 2019

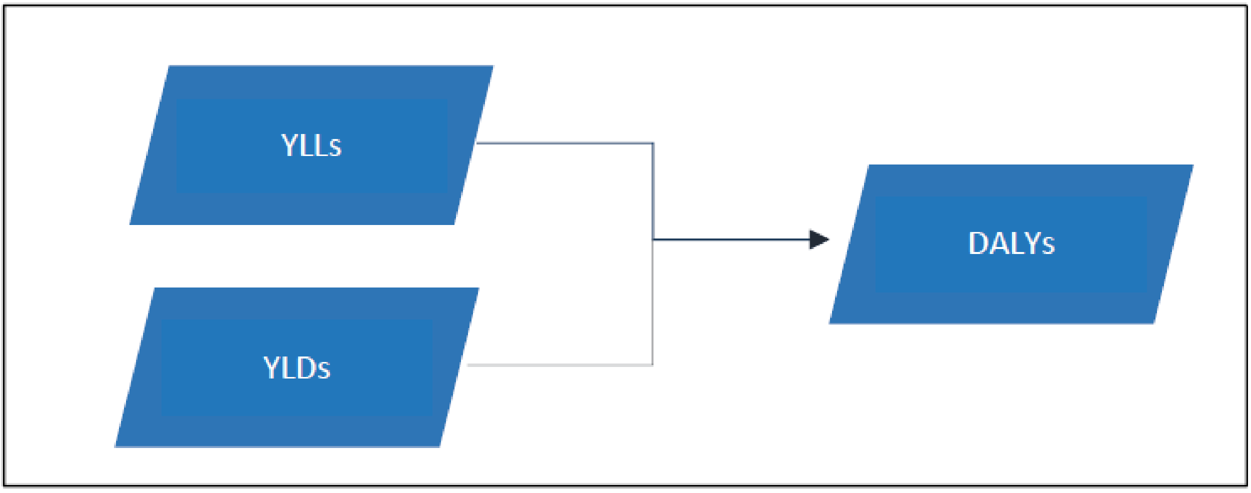

Section 7.3. Specific non-fatal modelling descriptions

Section 7.3.1. Diabetes mellitus

Section 7.3.1.1. Prevalence

The flow diagrams displayed below show how the steps taken in calculating the prevalence of diabetes mellitus

Prevalence is calculated for overall diabetes mellitus, then type 1 diabetes and finally type 2 diabetes. The approach to calculating prevalence of overall diabetes mellitus is shown in the following flowchart:

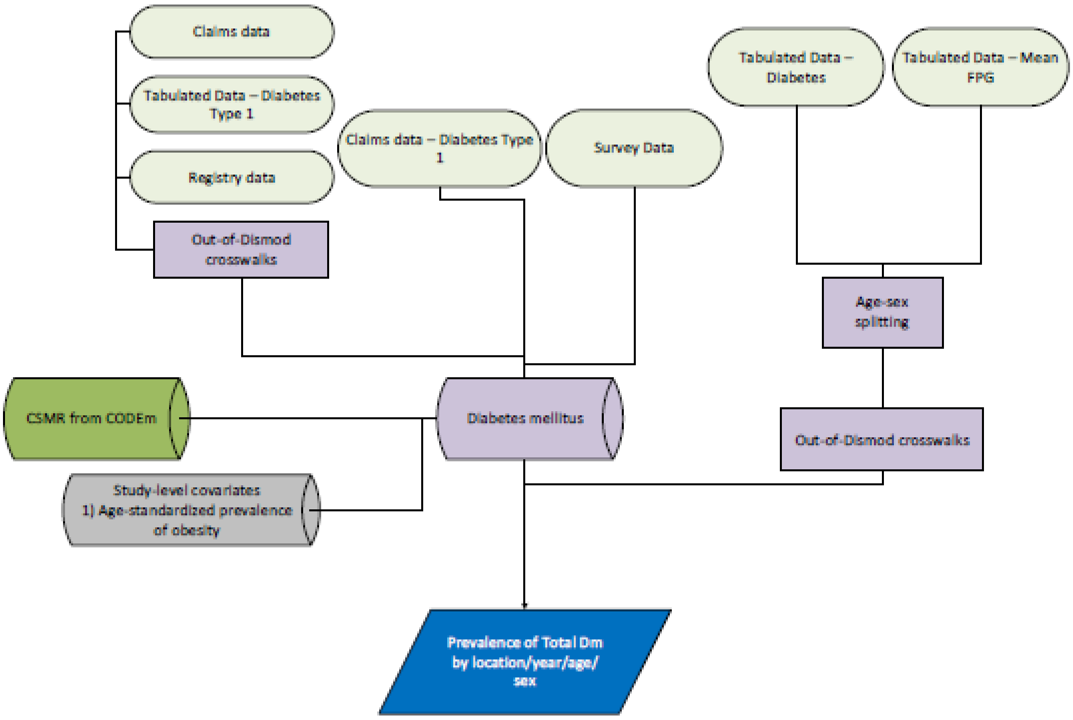

CSMR=cause-specific mortality ratio.

The approach to calculating the prevalence of type 1 diabetes mellitus is shown in the following flowchart:

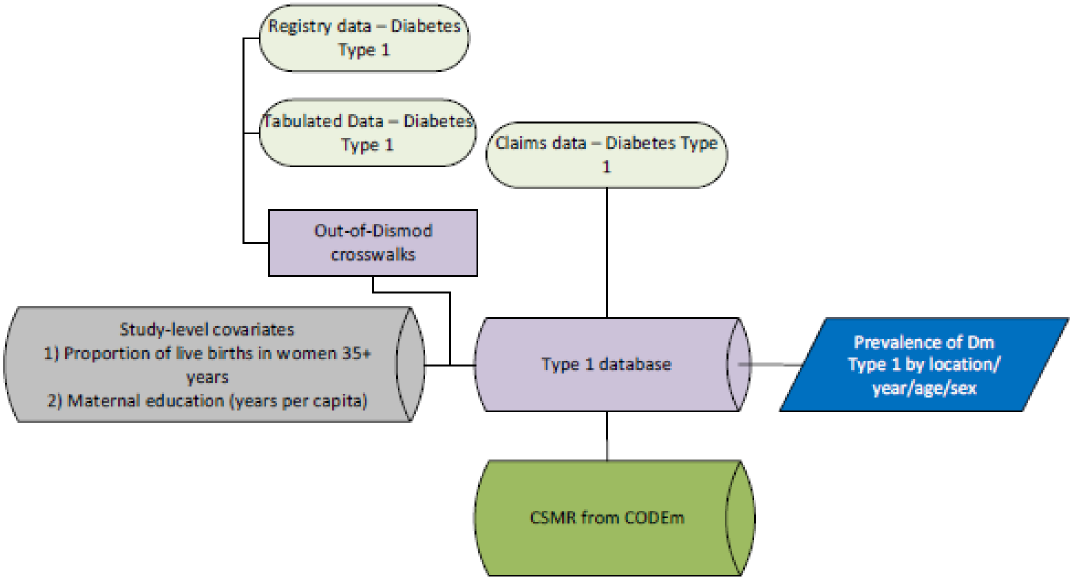

Finally, the prevalence of type 2 diabetes is determined subtracting that of type 1 from total diabetes.

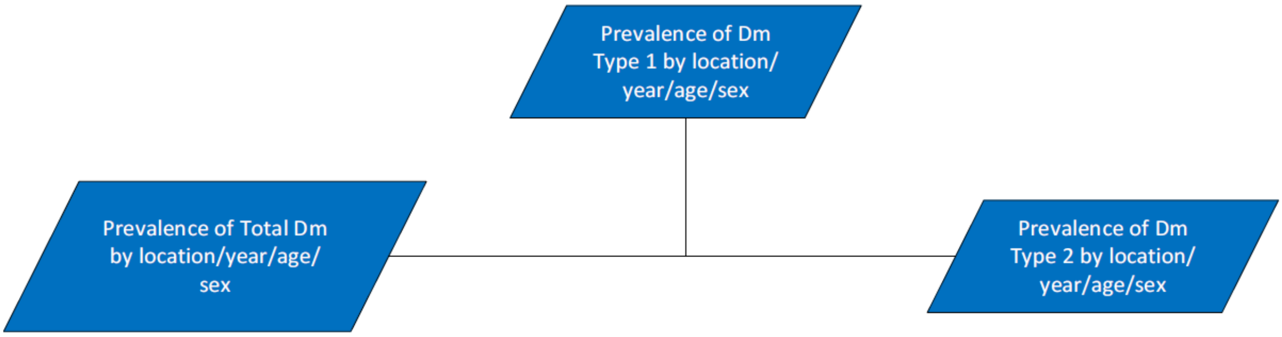

Section 7.3.1.2. Sequelae of diabetes

The following flow diagram shows the process, once prevalence has been determined, of calculating the frequency of sequelae of diabetes and their incorporation into YLDs and DALYs.

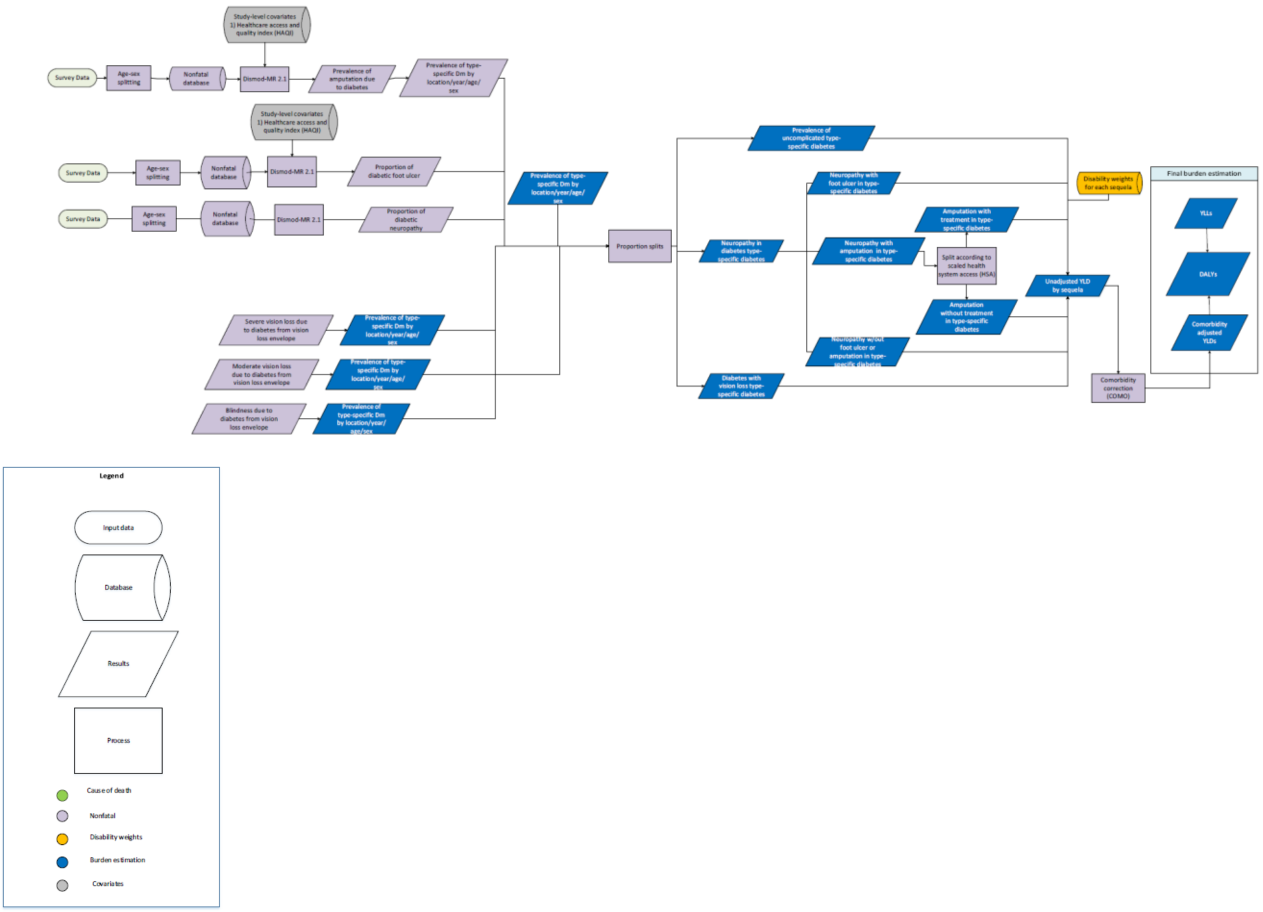

Section 7.3.1.3. Case definition

The case definitions and diagnostic criteria for diabetes, overall and by type, are presented below.

<u>Overall diabetes mellitus</u>

Criterion	Definition
1. Diabetes mellitus parent	Fasting plasma glucose (FPG) ≥126 mg/dL (7 mmol/L), or reporting to be on treatment with drugs or insulin for diabetes, or persons <15 years who are diagnosed by physicians and identified through a diabetic registry or hospital records

Diabetes mellitus type 1

Criterion	Definition
1. Diabetes mellitus type 1	Cases of type 1 DM diagnosed by physicians and identified through a diabetic registry or hospital records
2. Uncomplicated diabetes mellitus type 1	Cases of type 1 DM that do not have any of the following complications: neuropathy, foot ulcer, leg amputation, or vision loss
3. Diabetic neuropathy among diabetes mellitus type 1	Cases of type 1 DM that experience diagnosable neuropathy
4. Diabetic foot due to neuropathy among diabetes mellitus type 1	Cases of type 1 DM that currently have a foot ulcer
5. Diabetic neuropathy and amputation with treatment among diabetes mellitus type 1	Cases of type 1 DM that have had a leg amputation above or below the knee, with treatment consisting of a prosthetic limb
6. Diabetic neuropathy and amputation without treatment among diabetes mellitus type 1	Cases of type 1 DM that have had a leg amputation above or below the knee, with no prosthetic limb
7. Moderate vision impairment due to diabetes mellitus type 1	Cases of type 1 DM that have moderate vision loss due to diabetic retinopathy
8. Severe vision impairment due to diabetes mellitus type 1	Cases of type 1 DM that have severe vision loss due to diabetic retinopathy
9. Blindness due to diabetes mellitus type 1	Cases of type 1 DM that have blindness due to diabetic retinopathy

Diabetes mellitus type 2

Criterion	Definition
1. Diabetes mellitus type 2 parent	Fasting plasma glucose (FPG) ≥126 mg/dL (7 mmol/L) or reporting to be on drug or insulin treatment for type 2 diabetes
2. Uncomplicated diabetes mellitus type 2	Cases of DM type 2 that do not have any of the following complications: neuropathy, foot ulcer, leg amputation, or vision loss
3. Diabetic neuropathy among diabetes mellitus type 2	Cases of DM type 2 that experience diagnosable neuropathy
4. Diabetic foot due to neuropathy among diabetes mellitus type 2	Cases of DM type 2 that currently have a foot ulcer

5. Diabetic neuropathy and amputation with treatment among diabetes mellitus type 2	Cases of DM type 2 that have had a leg amputation above or below the knee, with treatment consisting of a prosthetic limb
6. Diabetic neuropathy and amputation without treatment among diabetes mellitus type 2	Cases of DM type 2 that have had a leg amputation above or below the knee, with no prosthetic limb
7. Moderate vision impairment due to diabetes mellitus type 2	Cases of DM type 2 that have moderate vision loss due to diabetic retinopathy
8. Severe vision impairment due to diabetes mellitus type 2	Cases of DM type 2 that have severe vision loss due to diabetic retinopathy
9. Blindness due to diabetes mellitus type 2	Cases of DM type 2 that have blindness due to diabetic retinopathy

Section 7.3.1.4 Data seeking

A systematic review of the literature for Diabetes mellitus, type 1 diabetes mellitus, and type 2 diabetes mellitus was done for GBD 2019 with the following search terms:

Diabetes mellitus search string: (diabetes[TI] AND (prevalence[TIAB] OR incidence[TIAB])) OR ('Diabetes Mellitus'[MeSH Terms] AND 'epidemiology'[MeSH Terms]) OR (diabetes[TI] AND 'epidemiology'[MeSH Terms]) NOT gestational[All Fields] NOT ('neoplasms'[MeSH Terms] OR 'neoplasms'[All Fields] OR 'cancer'[All Fields]) NOT ('mice'[MeSH Terms] OR 'mice'[All Fields]) NOT ('schizophrenia'[MeSH Terms] OR 'schizophrenia'[All Fields]) NOT ('emigrants and immigrants'[MeSH Terms] OR ('emigrants'[All Fields] AND 'immigrants'[All Fields]) OR 'emigrants and immigrants'[All Fields] OR 'immigrants'[All Fields]) NOT ('pregnancy'[MeSH Terms] OR 'pregnancy'[All Fields] OR 'gestation'[All Fields]) NOT ('rats'[MeSH Terms] OR 'rats'[All Fields] OR 'rat'[All Fields]) NOT ('kidney'[MeSH Terms] OR 'kidney'[All Fields]) NOT renal[All Fields] NOT ('vitamins'[Pharmacological Action] OR 'vitamins'[MeSH Terms] OR 'vitamins'[All Fields] OR 'vitamin'[All Fields])

and

FPG search string: ((“glucose”[Mesh] OR “hyperglycemia”[Mesh] OR “prediabetic state”[Mesh]) AND "Geographic Locations"[Mesh] NOT "United States"[Mesh]) AND ("humans"[Mesh] AND "adult"[MeSH]) AND ("Data Collection"[Mesh] OR "Health Services Research"[Mesh] OR "Population Surveillance"[Mesh] OR "Vital statistics"[Mesh] OR "Population"[Mesh] OR "Epidemiology"[Mesh] OR surve*[TiAb]) NOT Comment[ptyp] NOT Case Reports[ptyp]) NOT "hospital"[TiAb]

New sources identified are added to information in the Global Health Data Exchange (GHDx) for multi-country survey programs, national surveys, and longitudinal studies that were tagged with either fasting plasma glucose (FPG) or diabetes mellitus.

To capture any remaining sources not identified in the GHDx or in PubMed, we looked to other leaders in the field to ensure our datasets were as comprehensive as possible. These included data sources used by other research groups that report on the global burden of diabetes, microdata from not-yet-published national studies, and publications that were not captured in the PubMed search string.

Section 7.3.1.4.1. Source counts (global and the Americas)

Global sources counts for non-fatal estimations for diabetes mellitus are shown overall and for type 1 diabetes below:

Diabetes mellitus

Measure	Total sources	Countries with data
All measures	1289	171
Incidence	214	77
Prevalence	1020	155
Proportion	75	42
Relative risk	1	1
Standardised mortality ratio	5	4
With-condition mortality rate	6	5

Type 1 diabetes mellitus

Measure	Total sources	Countries with data
All measures	193	74
Incidence	163	73
Prevalence	32	14
Standardised mortality ratio	4	4

Non-fatal source counts in the Americas are shown in the Table S2:

Table S2. Non-fatal source counts for diabetes in the Americas

Country	Source count
Argentina	11
Chile	10
Uruguay	3
Canada	7
USA	39
Barbados	6
Belize	2
Cuba	3
Dominica	2
Dominican Republic	2
Jamaica	6
Saint Lucia	1
Suriname	2
Trinidad and Tobago	2
Bolivia	1
Ecuador	6
Peru	9
Colombia	7

Costa Rica	4
El Salvador	2
Guatemala	4
Honduras	2
Mexico	16
Panama	1
Venezuela	5
Brazil	23
Paraguay	3
Bermuda	1
Greenland	2
Puerto Rico	3
Virgin Islands	2

Section 7.3.1.5. Data inputs

Section 7.3.1.5.1 Overall diabetes mellitus

To incorporate all available data related to population-representative estimates of diabetes, we accepted other measures of blood sugar (glycated haemoglobin A1c, oral glucose tolerance test, post-prandial glucose test) to define diabetes and mean fasting plasma glucose (FPG) in a population when data on diabetes was not available as data inputs.

Data inputs came from four types of sources:

- Estimates of diabetes in a representative population
- Estimates of mean FPG in a representative population
- Individual-level data of fasting plasma glucose measured from surveys
- Insurance data, claims, from the USA and Taiwan (province of China)

When a study reported both mean fasting plasma glucose (FPG) and prevalence of diabetes, we used the prevalence of diabetes. Where possible, individual-level data from a cohort superseded any data described in a study. Individual-level data were collapsed and aggregated to produce estimates for each age group, sex, location, and year a survey is conducted.

We used prevalence of obesity as a covariate.

Section 7.3.1.5.2. Diabetes type 1

To incorporate all available data related to population-representative estimates of diabetes type 1, we accepted data that reported diabetes type 1, juvenile-onset diabetes, and insulin-dependent diabetes.

Data inputs comes from two types of sources:

- Estimates of type 1 diabetes mellitus in a representative population

- Diabetic registries

Section 7.3.1.5.3. Diabetes type 2

Only 20% of diabetes mellitus estimates are available by type. Furthermore, while the sources report type 2 diabetes mellitus, the diagnostic criteria in the methodological sections are not sufficiently specific. Thus, we calculated estimates of diabetes mellitus type 2 by subtracting the estimates of diabetes mellitus type 1 from estimates overall diabetes mellitus for each age, sex, and location from 1990 to 2019.

Section 7.3.1.6. Data processing

Section 7.3.1.6.1. Overall diabetes mellitus

We performed several processing steps to the data in order to address sampling and measurement inconsistencies that will ensure the data are comparable across data sources and between high fasting plasma glucose modelling efforts.

Small sample size: Estimates in a sex and age group with a sample size <30 persons were considered a small sample size. In order to avoid small sample size problems that may bias estimates, data were collapsed into the next age group in the same study until the sample size reached at least 30 persons. The intent of collapsing the data is to preserve as much granularity between age groups as possible, which determined whether the collapse occurred with a younger or older age group. If the entire study sample consisted of <30 persons and did not include a population-weight, the study was excluded from the modelling process. The estimates were re-calculated if case count and sample size were available, or the population-weighted estimate was calculated when only sample size was available.

Mean FPG processing: We used an ensemble distribution to estimate the prevalence of diabetes based on mean FPG in locations where data on prevalence of diabetes were not available. Essentially, we constructed a distribution based on unit-level data available in 31 different countries. Then we predicted out the prevalence of diabetes by age and sex. This provides the conversion of mean FPG to prevalence of diabetes defined as FPG >126 mg/dL (7 mmol/L). Because this definition is not consistent with our reference case definition (which also includes those on treatment), we then apply an adjustment to adjust these datapoints to the reference case definition. For information on how these adjustments are made, please see the section, Age splitting and bias adjustments.

In GBD 2019, we improved the bias adjustment methods to allow a more direct comparison between different case definitions and/or study designs. In GBD 2017, we constructed ratios between alternative case definitions and the reference case definition using data from surveys that measured glucose level based on different glucose tests on a single person. For insurance data, we allowed DisMod to estimate the adjustment. In GBD 2019, we constructed ratios between alternative case definitions and the reference case definition using data from surveys that measured glucose level based on different glucose tests on a single person or between survey and the insurance claims data. However, we assume that claims data in persons 15 years. We used MR-BRT analysis to adjust for bias due to commercial insurance or use of alternative case definitions. We performed this analysis in logit-space due to the high prevalence of diabetes (from simulations we learned that for prevalence greater than 50% the log ratio method is biased).

The process of adjusting for non-reference data using MR-BRT with the logit-transformation method is described below:

1. Identify datapoints with overlapping year, age, sex, and location between alternative case definition and reference case definition
2. Logit transform overlapping datapoints of alternative and reference case definitions
3. Convert overlapping datapoints into a difference in logit space using the following equation: logit(alternative)–logit(reference)
4. Use the delta method to compute standard errors of overlapping datapoints in logit space, then calculate standard error of logit difference using the following equation: √((variance of alternative)+(variance of reference))
5. Using MR-BRT, conduct a random effects meta-regression to obtain the pooled logit difference of alternative to reference
6. Apply the pooled logit difference to all datapoints of alternative case definitions using the
7. following equation: New estimate = inverse.logit((logit(alternative))-(pooled logit difference))
8. Calculate new standard errors using the delta method, accounting for gamma (between-study heterogeneity)

Table S3. MR-BRT crosswalk adjustment factors for total diabetes

Data input	Reference or alternative case definition	Adjustment factor (95% UI)**
FPG >126 mg/dl (7 mmol/L) or Tx	Reference	---
HbA1c >6.5%	Alternative	0.74 (0.33 – 1.66)
HbA1c >6.4% or Tx	Alternative	1.06 (0.57 – 1.96)
HbA1c >6%	Alternative	2.01 (0.65 – 6.20)
HbA1c >6.5% or Tx	Alternative	0.92 (0.52 – 1.63)
FPG >100 mg/dl (5.6 mmol/L) or Tx	Alternative	4.98 (2.89 – 8.58)
FPG >100 mg/dl (5.6 mmol/L)	Alternative	4.72 (2.76 – 8.08)
FPG >110 mg/dl (6.1 mmol/L) or Tx	Alternative	1.99 (1.55 – 2.54)
FPG >110 mg/dl (6.1 mmol/L)	Alternative	1.8 (1.31 – 2.47)
FPG >115 mg/dl (6.4 mmol/L) or Tx	Alternative	1.46 (1.25 – 1.70)
FPG >120 mg/dl (6.7 mmol/L)	Alternative	0.997 (0.77 – 1.29)
FPG >121 mg/dl (6.7 mmol/L)	Alternative	0.96 (0.77 – 1.20)
FPG >126 mg/dl (7 mmol/L)	Alternative	0.78 (0.60 – 1.02)
FPG >140 mg/dl (7.8 mmol/L) or Tx	Alternative	0.76 (0.62 – 0.93)
FPG >144 mg/dl (8 mmol/L) or Tx	Alternative	0.72 (0.57 – 0.91)
OGTT >180 mg/dl (10 mmol/L) or Tx	Alternative	2.28 (1.57 – 3.30)
OGTT >200 mg/dl (11.1 mmol/L)	Alternative	1.5 (1.04 – 2.15)
OGTT >200 mg/dl (11.1 mmol/L) or Tx	Alternative	1.5 (1.04 – 2.18)
FPG >110 mg/dl (6.1 mmol/L) or OGTT	Alternative	4.92 (2.94 – 8.24)

>200 mg/dl (11.1 mmol/L)		
FPG >126 mg/dl (7 mmol/L) or OGTT >200 mg/dl (11.1 mmol/L)	Alternative	1.85 (1.49 – 2.30)
FPG >126 mg/dl (7 mmol/L) or OGTT >200 mg/dl (11.1 mmol/L) or Tx	Alternative	1.86 (1.49 – 2.33)
FPG >126 mg/dl (7 mmol/L) or OGTT >220 mg/dl (12.2 mmol/L)	Alternative	1.44 (1.22 – 1.70)
FPG >144 mg/dl (8 mmol/L) or OGTT >200 mg/dl (11.1 mmol/L) or Tx	Alternative	1.53 (1.06 – 2.22)
FPG >140 mg/dl (7.8 mmol/L) or OGTT >200 mg/dl (11.1 mmol/L) or Tx	Alternative	1.54 (1.06 – 2.24)
FPG >140 mg/dl (7.8 mmol/L) or OGTT >200 mg/dl (11.1 mmol/L)	Alternative	1.53 (1.06 – 2.22)
FPG >126 mg/dl (7 mmol/L) or OGTT >200 mg/dl (11.1 mmol/L) or HbA1c >6.1%	Alternative	3.67 (1.35 – 10.00)

Age splitting and bias adjustments: Reported estimates of prevalence were split by age and sex where possible. First, if studies reported prevalence for broad age groups by sex, and by specific age groups but for both sexes combined, age-specific estimates were split by sex using the sex ratio from within the study. Second, input data reporting prevalence for both sexes that could not be split using a within-study ratio were split using a sex ratio derived from a meta-analysis of existing sex-specific data using MR-BRT. The female to male ratio for diabetes was 0.85 (0.61–1.09). Finally, after the application of bias adjustments, where studies reported estimates across age groups spanning 25 years or more, these were split into five-year age groups using the prevalence age pattern estimated by DisMod-MR 2.1 in a model that contained the subset of data with age range less than 25 years.

Section 7.3.1.6.2. Diabetes type 1

Based on assumption that claims data in persons <15 years are type 1 diabetes and that 100% of diabetics are captured in this age group, we make no adjustments to data in these ages. Claims data are reported as prevalence.

There are a number of different sources and ascertainment methods that were used to identify type 1 diabetics. The majority of data that are reported in the literature are from a diabetic registry, hospital discharge data review, physician interview, or insulin use. We assumed that there is no systematic bias between these sources and consider sources identified through these methods as reference. For the other sources that use alternative ascertainment techniques (eg, pharmacy reports, diabetic camps, school reports), there was not sufficient amount of data to perform an analysis on each individual type, and the model had relatively few datapoints in locations where these approaches were used. So we collapsed all alternative sources and treated the estimates from these sources as defined as an alternative case definition.

Section 7.3.1.7. Modelling strategy overall

Section 7.3.1.7.1. Diabetes mellitus

For GBD 2019, we estimated the overall prevalence of diabetes using DisMod MR-2.1, a Bayesian meta-regression. DisMod-MR produces estimates of the prevalence of diabetes for each age, sex, geographical location, and year. We used data that reported prevalence and incidence for diabetes mellitus.

Section 7.3.1.7.2. Diabetes mellitus type 1

For GBD 2019, we estimated the overall prevalence of diabetes also using DisMod MR-2.1. We used data that reported incidence, standardised mortality ratio, and prevalence data in claims data for persons <15 years for diabetes mellitus type 1. We decided to not include reported type 1 diabetes prevalence in non-claims sources because we found that their estimates of prevalence and incidence were inconsistent. We decided to trust the incidence data and thus had to exclude the prevalence data from the model. Similarly, we did not include prevalence of diabetes type 1 in people >15 years from claims sources, because of poor reporting on type of diabetes.

Section 7.3.1.7.3. Diabetes type 2

Only 20% of diabetes mellitus estimates are available by type. Furthermore, while the sources report type 2 diabetes mellitus, the diagnostic criteria in the methodological sections are not sufficiently specific. Thus, we calculated estimates of diabetes mellitus type 2 by subtracting the estimated prevalence of diabetes mellitus type 1 from estimated prevalence of overall diabetes mellitus for each age, sex, and location from 1990 to 2019.

Section 7.3.1.8. Outcomes

Section 7.3.1.8.1 Data seeking

Amputation due to diabetes mellitus

A systematic review of the literature was performed for GBD 2017 with the following search terms:

('Diabetes Mellitus'[MeSH Terms] OR ('diabetes'[All Fields] AND 'mellitus'[All Fields]) OR 'Diabetes Mellitus'[All Fields]) AND 'amputation'[All Fields] AND (proportion OR prevalence OR incidence) NOT gestational NOT cancer NOT mice NOT schizophrenia NOT immigrants NOT gestation NOT rat NOT kidney NOT renal NOT vitamin

Diabetic neuropathy

A systematic review of the literature was performed for GBD 2017 with the following search terms:

("Diabetes Mellitus"[MeSH Terms] OR ("diabetes"[All Fields] AND "mellitus"[All Fields]) OR "Diabetes Mellitus"[All Fields]) AND neuropathy[All Fields] AND (proportion OR prevalence OR incidence) NOT gestational NOT cancer NOT mice NOT schizophrenia NOT immigrants NOT gestation NOT rat NOT kidney NOT renal NOT vitamin)

Diabetic foot ulcer

A systematic review of the literature was performed for GBD 2017 with the following search terms:

((("Diabetes Mellitus"[MeSH Terms] OR ("diabetes"[All Fields] AND "mellitus"[All Fields]) OR "Diabetes Mellitus"[All Fields] OR "diabetes"[All Fields]) AND ("foot"[MeSH Terms] OR "foot"[All Fields]) AND ("ulcer"[MeSH Terms] OR "ulcer"[All Fields])) NOT ("neoplasms"[MeSH Terms] OR "neoplasms"[All Fields] OR "cancer"[All Fields]) NOT ("mice"[MeSH Terms] OR "mice"[All Fields]) NOT ("emigrants and immigrants"[MeSH Terms] OR ("emigrants"[All Fields] AND "immigrants"[All Fields]) OR "emigrants and immigrants"[All Fields] OR "immigrants"[All Fields]) NOT ("pregnancy"[MeSH Terms] OR "pregnancy"[All Fields] OR "gestation"[All Fields]) NOT ("vitamins"[Pharmacological Action] OR "vitamins"[MeSH Terms] OR "vitamins"[All Fields] OR "vitamin"[All Fields]) NOT renal[All Fields] NOT ("kidney"[MeSH Terms] OR "kidney"[All Fields]) AND (proportion[All Fields] OR "incidence"[All Fields] OR "prevalence"[All Fields]) NOT ("schizophrenia"[MeSH Terms] OR "schizophrenia"[All Fields]) NOT ("rats"[MeSH Terms] OR "rats"[All Fields] OR "rat"[All Fields]))

Section 7.3.1.9. Modelling strategy

For GBD 2019, we estimated amputation due to diabetes mellitus, diabetic neuropathy, and diabetic foot for diabetes mellitus type 1 and diabetes mellitus type 2 using DisMod MR-2.1. DisMod-MR produces estimates of the prevalence of diabetes for each age, sex, geographical location, and year. We then multiply all proportion draws from neuropathy/foot/amputation models by the parent diabetes model so that all estimates are in the same population-space.

We ensure that the sum of the prevalence for neuropathy due to diabetes mellitus, moderate vision loss due to diabetes mellitus, severe vision loss due to diabetes mellitus, and blindness due to diabetes mellitus does not exceed 90% of the prevalence of all diabetes mellitus. If the sum exceeds 90%, then we rescale the individual outcomes to 90%. We do not directly model vision loss. These estimates are derived as part of the vision loss impairment analyses based on data ascribing vision loss to underlying causes in population-based surveys. The diabetes process takes these estimates into account when estimating uncomplicated diabetes mellitus, amputation due to diabetes mellitus, diabetic neuropathy, and diabetic foot for diabetes mellitus type 1 and diabetes mellitus type 2.

We perform the same check to ensure that the prevalence of amputation due to diabetes mellitus and prevalence of foot ulcer due to diabetes mellitus does not exceed 90% of the prevalence of neuropathy due to diabetes mellitus. This treats foot ulcer and amputation as mutually exclusive categories by assuming a patient will not have both simultaneously.

From here, we calculate uncomplicated diabetes as the remainder of diabetes cases exclusive of neuropathy and vision loss. In addition, we estimate the prevalence of amputation due to diabetes is split into with and without treatment using scaled health systems access (HSA) values. For diabetic amputation, we calculated a distribution of treated versus untreated amputation, defined as receiving a prosthesis or not. We first rescaled the IHME estimates to be between 0 and 0.9, under the assumption that 10% of amputees will not receive a prosthetic, even in high-income countries. We based this assumption on the retrospective study by Moore et al, which found that about 80% of patients following major lower extremity amputation were fitted with prostheses in the authors' institutions from 1978 to 1986 in the USA. We then performed a population-weighted average of this country-specific value to obtain a proxy for the proportion of amputees that receive a prosthetic by super-region. Because these are rough estimates based on large assumptions, we applied confidence intervals of +/- 50% of the value to reflect our uncertainty.

Section 7.3.1.10. Severity distributions

We determined the disability weights for each sequela from the GBD disability weight survey. The table below illustrates the severity levels, lay descriptions, and associated disability weights applicable for outcomes related to diabetes mellitus type 1 and diabetes mellitus type 2:

Severity level	Lay description	DW (95% CI)
Uncomplicated diabetes mellitus	Has a chronic disease that requires medication every day and causes some worry, but minimal interference with daily activities	0.049 (0.031–0.072)
Diabetic neuropathy	Has pain, tingling, and numbness in the arms, legs, hands, and feet. The person sometimes gets cramps and muscle weakness.	0.133 (0.089–0.187)
Diabetic neuropathy with diabetic foot	Has a sore on the foot that is swollen and causes some difficulty in walking.	*a*
Diabetic neuropathy with treated amputation	Has lost part of one leg, leaving pain and tingling in the stump. The person has an artificial leg that helps in moving around.	*a*
Diabetic neuropathy with untreated amputation	Has lost part of one leg, leaving pain and tingling in the stump. The person does not have an artificial leg, has frequent sores, and uses crutches.	*a*
Moderate vision loss due to diabetes mellitus	Has vision problems that make it difficult to recognise faces or objects across a room.	0.031 (0.019–0.049)
Severe vision loss due to diabetes mellitus	Has severe vision loss, which causes difficulty in daily activities, some emotional impact (for example worry), and some difficulty going outside the home without assistance.	0.184 (0.125–0.259)
Blindness due to diabetes mellitus	Is completely blind, which causes great difficulty in some daily activities, worry and anxiety, and great difficulty going outside the home without assistance.	0.187 (0.124–0.26)

[a] *The disability weights are produced from a combination of two health states: neuropathy and diabetic foot/amputation*

Section 7.3.2. Chronic kidney disease

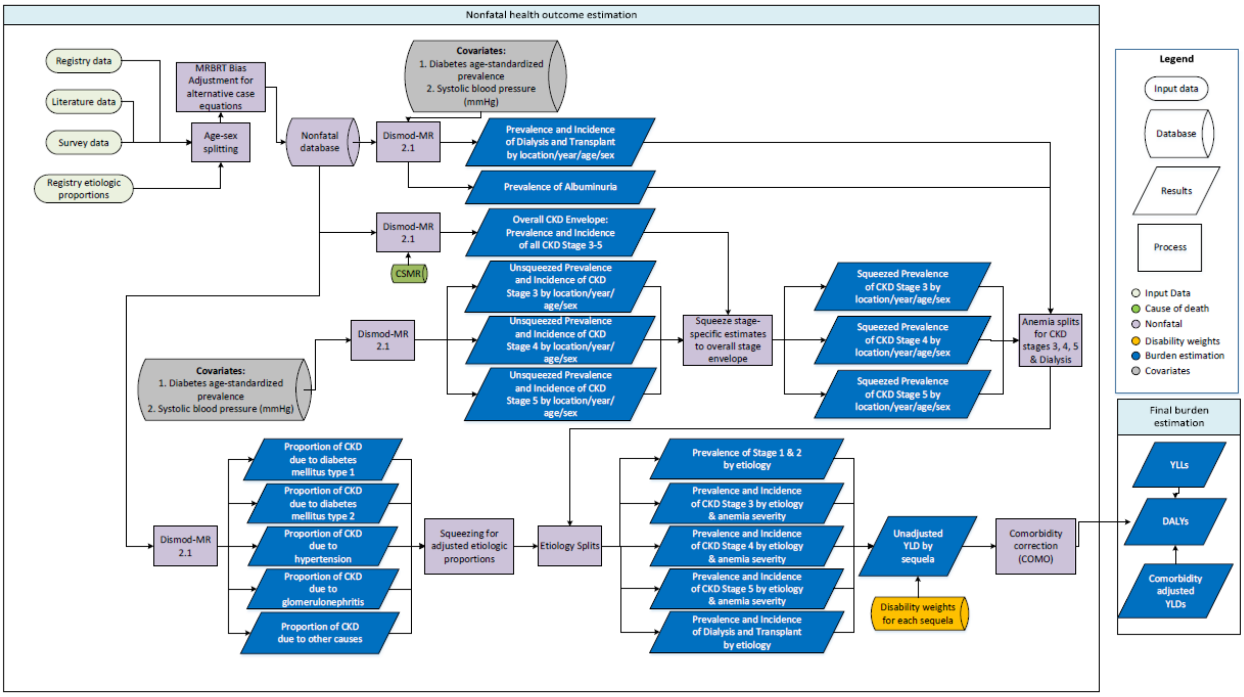

Section 7.3.2.1 Case definition

Chronic kidney disease (CKD) is defined as a permanent loss of kidney function as indicated by estimated glomerular filtration rate (eGFR) and urinary albumin to creatinine ratio (ACR). The GBD study considers six stages of CKD as defined by degree of loss of kidney function or receipt of kidney replacement therapy: CKD stages 1&2 (eGFR >60ml/min/1.73m^2 and ACR >30 mg/g), CKD Stage 3 (eGFR 30– 60 ml/min/1.73m^2), CKD Stage 4 (eGFR 15–30 ml/min/1.73m^2), CKD Stage 5 (eGFR <15ml/min/1.73 m^2, not on kidney replacement therapy), maintenance dialysis, and kidney transplantation.[1] The ICD-10 codes associated with CKD include N18.1-N18.9.

Section 7.3.2.2. Input data

Model inputs This literature search used PubMed search terms ((((("chronic kidney disease"[Title/Abstract]) AND prevalen*[Title/Abstract]) AND ("1980/1/1"[Date - Publication] : "3000"[Date - Publication])) NOT ((animals[MeSH] NOT humans[MeSH])))).

The exclusion criteria were:

- Studies clearly not representative of the national population
- Studies that did not provide primary data on epidemiological parameters, eg, a commentary piece
- Studies of a specific aetiology of CKD only

This literature search was augmented by identification of population-based surveys that measured kidney function. For maintenance dialysis and kidney transplantation, data were largely obtained from kidney registry reports.

Data inputs for chronic kidney disease

Measure	Total sources	Countries with data
All measures	1646	122
Prevalence	1204	120
Incidence	1072	90
Excess mortality rate	67	13
With-condition mortality rate	4	4
Proportion	367	55

Section 7.3.2.3. Data processing

Age-sex and sex Split

In some cases, data are reported by only age or only sex, but not both. For example, a study may have included the proportion of males and females with stage 3 CKD and then separately reported the proportion of both sexes by smaller age bins (eg, 40–44, 45–49) that have stage 3 CKD. In these cases, we perform an age-sex split by utilising proportions within the study to disaggregate the data.

When there is no information by sex in a study, we instead perform a sex-split on the data by applying separate sex proportions. In order to obtain an appropriate age-pattern with which to age-split input data, we first ran a DisMod-MR 2.1 model containing only age-specific data. We then used age-pattern by super-region from this model to age-split dialysis input data, thereby allowing for variation in the age-pattern by location. After age-splitting, we ran a model on all processed data, including age-split data and age-specific data, to obtain final estimates of dialysis incidence and prevalence by location, year, age, and sex. For dialysis, remission data for dialysis were calculated as the ratio of the incidence of kidney transplantation to prevalence of dialysis at the gender-, age-, and country-matched level.

Modelled excess mortality data

For the Stage 3-5 CKD, we implemented a new method of modelling excess mortality rate (EMR). In previous rounds, priors on EMR were estimated in DisMod by matching prevalence datapoints with their corresponding CSMR values within the same age, sex, year, location (estimating EMR by dividing CSMR by prevalence).

However, for many causes, DisMod estimated a rather unrealistic pattern of EMR compared to an expected pattern of decreasing EMR with greater access to quality health care. Such unexpected patterns often signal inconsistencies between CSMR estimates and the measures of prevalence and/or incidence. In an effort to provide greater guidance to DisMod on the expected pattern of EMR, EMR data generated in the previous round were modelled using the MR-BRT approach by age and sex with a prior on Healthcare Access and Quality (HAQ) Index having a negative coefficient. Results from MR-BRT were then predicted for each location year, sex and for ages 0, 10, 20 ….100.

We also included HAQ Index as a country-level covariate to inform EMR with a mean and standard deviation produced from MR-BRT.

Bias adjustments

In GBD 2019, we improved the bias adjustment methods by utilising a MR-BRT model outside of DisMod to allow a more direct comparison between different case definitions and/or study designs. In GBD 2017, these adjustments were performed within DisMod.

Glomerular filtration rate (GFR) can be estimated using a variety of equations that lead to different prevalence estimates. Our CKD reference equation is the CKD-Epi Creatinine equation. We also included data estimated with the Modification of Diet in Renal Disease (MDRD) and the Cockcroft-Gault (CG) equation. For children, the Schwartz equation was used as the reference.

Section 7.3.2.4. Modelling strategy

CKD Stage Models

We run a separate DisMod-MR 2.1 model to produce estimates by age, sex, year, and country for each stage of CKD, along with an aggregate CKD Stage III-V model. Each separate CKD Stage model was then rescaled to the aggregate CKD model for every age, sex, year, and country. This was done in order to enforce more consistency in the prevalence and incidence between stage models.

Section 7.3.2.5. CKD aetiology proportion models

CKD aetiology proportion models

To model aetiology proportions of CKD, we utilised two separate types of data.

The first are data from end-stage kidney registries used to estimate the proportion of each aetiology for those on dialysis or with kidney transplants.

The second set of data come from the Geisinger Health System in Pennsylvania. These data contain age-sex-stage-specific aetiology proportions that allowed differential aetiological composition of CKD across stages for disease progression. These data were used for Stages 1&2, Stage 3, Stage 4, and Stage 5 CKD. For each individual with CKD, we scanned their history of recorded ICD codes to identify ICD codes for primary kidney diseases. We used this information to map individuals to GBD aetiologies by stage of CKD; individuals with CKD but with no history of a primary kidney disease ICD code were classified as having CKD of unknown aetiology. We ran a multinomial logistic regression including sex and a non-linear term for age to predict the probability of each aetiology by age and sex for each stage of CKD (1&2, 3, and 4/5 combined). For each stage, aetiology, age, and sex, we converted this probability into the proportion of CKD due to the given aetiology and applied these proportions to the prevalence of CKD for the same stage, age, and sex category to estimate the prevalence of each stage of CKD by aetiology, age, and sex. The ICD to GBD aetiology map utilised in this analysis is as follows:

CKD aetiology	ICD-9 Codes	ICD-10 Codes
Type 1 diabetes	250.41, 250.43	E10.2, E10.21, E10.22, E10.29
Type 2 diabetes	250.40, 250.42	E11.2, E11.21, E11.22, E11.29

Glomerulonephritis	581, 581.0, 581.1, 581.2, 581.3, 581.8, 581.81, 581.89, 581.9, 582, 582.0, 582.1, 582.2, 582.4, 582.8, 582.81, 582.89, 582.9, 583, 583.0, 583.1, 583.2, 583.4, 583.6, 583.7, 583.8, 583.81, 583.89, 583.9	N02, N02.0, N02.1, N02.2, N02.3, N02.4, N02.5, N02.6, N02.7, N02.8, N02.9, N03, N03.0, N03.1, N03.2, N03.3, N03.4, N03.5, N03.6, N03.7, N03.8, N03.9, N04, N04.0, N04.1, N04.2, N04.3, N04.4, N04.5, N04.6, N04.7, N04.8, N04.9, N05, N05.0, N05.1, N05.2, N05.3, N05.4, N05.5, N05.6, N05.7, N05.8, N05.9, N06, N06.0, N06.1, N06.2, N06.3, N06.4, N06.5, N06.6, N06.7, N06.8, N06.9
Hypertension	403, 403.0, 403.00, 403.01, 403.1, 403.10, 403.11, 403.6, 403.9, 403.90, 403.91, 404, 404.0, 404.00, 404.01, 404.02, 404.03, 404.1, 404.10, 404.11, 404.12, 404.13, 404.9, 404.90, 404.91, 404.92, 404.93	I12, I12.0, I12.1, I12.2, I12.9, I13, I13.0, I13.1, I13.10, I13.11, I13.2, I13.9
Other	589, 589.0, 589.1, 589.9, 753.0, 753.1, 753.10, 753.11, 753.12, 753.13, 753.14, 753.15, 753.16, 753.17, 753.19, 753.2, 753.20, 753.21, 753.22, 753.23, 753.29, 753.3, 283.11, 710.0, 753.0, 753.21, 753.22, 753.29	N07, N07.0, N07.1, N07.2, N07.3, N07.4, N07.5, N07.6, N07.7, N07.8, N07.9, N08, N08.0, N08.1, N08.2, N08.3, N08.4, N08.5, N08.8, N15.0, Q61, Q61.0, Q61.00, Q61.01, Q61.02, Q61.1, Q61.11, Q61.19, Q61.2, Q61.3, Q61.4, Q61.5, Q61.8, Q61.9, Q62, Q62.0, Q62.1, Q62.10, Q62.11, Q62.12, Q62.2, Q62.3, Q62.31, Q62.32, Q62.39, Q62.4, Q62.5, Q62.6, Q62.60, Q62.61, Q62.62, Q62.63, Q62.69, Q62.7, Q62.8, D59.3, M31.31, M32.14, M32.15, N11.9, N13.70, N13.8, Q60.2, Q63.8, N14.0, N14.1, N14.3, N25.89, N26.9, N28.0

In order to maintain consistency between GBD estimates of type 1 diabetes prevalence estimates and CKD due to type 1 diabetes prevalence estimates and generalise the results of the Geisinger analysis to all locations, we performed a location-specific correction for the proportion of CKD due to type 1 and type 2 diabetes. Type 1 diabetes makes up a larger proportion of total diabetes in the United States than it does in other locations. For each diabetic subtype (e) for a given location (l), age (a), and sex (g), the ratio of subtype-specific diabetes prevalence to total diabetes prevalence (r) was calculated as:

$$r_{e,l,a,g} = \frac{prevalence_{e,l,a,g}}{prevalence_{dm1,l,a,g} + prevalence_{dm2,l,a,g}}$$

This ratio is used to adjust the proportion of CKD due to a given diabetic subtype (p) for a given CKD stage (s), l, a, and g by scaling the predicted proportion of CKD due to that subtype (k) by the ratio of total DM due to e in l to the ratio of total DM due to e in the United States (USA).

$$p_{s,e,l,a,g} = k_{s,a,g} \times \frac{r_{e,l,a,s}}{r_{e,USA,a,s}}$$

The stage-specific approach utilised to estimate the prevalence of CKD stages is limited by the use of data from a single geographical region.

All CKD due to diabetes were forced to be type 1 diabetes under the age of 20.

For end-stage kidney disease on dialysis and end-stage kidney disease after transplant, we ran DisMod- MR 2.1 models to obtain estimates of proportions for each subtype by location, year, age, and sex. Data for CKD due to overall DM were more widely available than data by type of DM. Models for the proportion of CKD due to hypertension and diabetes included covariates for mean systolic blood pressure and the age-standardised prevalence of diabetes, respectively.

In order to make use of all available data, we modelled the proportion of CKD due to overall DM, DM type 1, and DM type 2. Proportion of CKD due to DM type 1 and DM type 2 were then scaled to sum to the proportion of overall DM at the gender, age, and country-matched level. The results from all subtype- specific models were adjusted so that estimates across the subtypes equaled 1 at each of 1000 draws.

These adjusted proportions were applied to the DisMod models for dialysis and transplant to obtain estimates of each of these entities by aetiology.

Section 7.3.2.6. Severity splits and disability weights

Estimates of prevalence and incidence are split using CKD aetiology proportion models, resulting in CKD estimates by stage and aetiology. Then a portion of each aetiology split for CKD stages III, IV, and V is attributed a disability weight associated with mild, moderate, or severe anaemia.[2]

Severity level	Lay description	Disability weight (95% CI)
Albuminuria	Asymptomatic	--
CKD stage III without anaemia	Asymptomatic	--
CKD stage III with mild anaemia	Feels slightly tired and weak at times, but this does not<br>interfere with normal daily activities.	0.004<br>(0.001–0.008)
CKD stage III with moderate anaemia	Feels moderate fatigue, weakness, and shortness of breath after exercise, making daily activities more difficult.	0.052<br>(0.034–0.076)

CKD stage III with severe anaemia	Feels very weak, tired, and short of breath, and has problems with activities that require physical effort or deep concentration.	0.149 (0.101–0.21)
CKD stage IV without anaemia	Tires easily, has nausea, reduced appetite, and difficulty sleeping.	0.104 (0.07–0.147)
CKD stage IV with mild anaemia		0.108 (0.072–0.151)
CKD stage IV with moderate anaemia		0.15 (0.103–0.207)
CKD stage IV with severe anaemia		0.237 (0.165–0.324)
CKD stage V without anaemia	Has lost a lot of weight and has constant pain. The person has no appetite, feels nauseated, and needs to spend most of the day in bed.	0.569 (0.389–0.727)
CKD stage V with mild anaemia		0.570 (0.391–0.727)
CKD stage V with moderate anaemia		0.591 (0.414–0.743)
CKD stage V with severe anaemia		0.631 (0.456–0.782)
End-stage kidney disease, on dialysis	Is tired and has itching, cramps, headache, joint pains, and shortness of breath. The person needs intensive medical care every other day lasting about half a day.	0.571 (0.397–0.725)
End-stage kidney disease, with kidney transplant	Sometimes feels tired and down, and has some difficulty with daily activities.	0.024 (0.014–0.039)

Note: the DWs for CKD 4 and 5 stages with anaemia are derived from a multiplicative function combining the CKD stage DW and the corresponding severity of anaemia DW

Section 8. Risk factor estimation

Section 8.1. Overview

The comparative risk assessment (CRA) conceptual framework was developed by Murray and Lopez,[13] who established a causal web of hierarchically organised risks or causes that contribute to health outcomes, which allows for quantification of risks or causes at any level in the framework. In GBD 2019,as in previous iterations of the GBD study, we evaluated a set of behavioural, environmental and occupational, and metabolic risks, in which risk-outcome pairs were included based on evidence rules. These risks were organised in four hierarchical levels, where Level 1 represents the overarching categories (behavioural, environmental and occupational, and metabolic) nested within Level 1 risks; Level 2 contains both single risks and risk clusters (such as child and maternal malnutrition); Level 3 contains the disaggregated single risks from within Level 2 risk

clusters (such as low birthweight and short gestation); and Level 4 details risks with the most granular disaggregation, such as for specific occupational carcinogens, the subcomponents of child growth failure (stunting, wasting, underweight), and suboptimal breastfeeding (discontinued and non-exclusive breastfeeding). At each level of risk, we evaluated whether risk combinations were additive, multiplicative, or shared common pathways for intervention. This approach allows the quantification of the proportion of risk-attributable burden shared with another risk or combination of risks and the measurement of potential overlaps between behavioural, environmental and occupational, and metabolic risks. To date in the GBD, we have not quantified the contribution of other classes of risk factors. We do provide some insights into the potential magnitude of distal social, cultural, and economic factors through an analysis of the relationship between risk exposures and development measured by using the Socio-demographicIndex (SDI) (see appendix section 12).

Two types of risk assessments are possible within the CRA framework: attributable burden and avoidable burden. Attributable burden is the reduction in current disease burden that would have beenpossible if past population exposure had shifted to an alternative or counterfactual distribution of risk exposure. Avoidable burden is the potential reduction in future disease burden that could be achieved by changing the current distribution of exposure to a counterfactual distribution of exposure. Murray and Lopez identified four types of counterfactual exposure distributions: (1) theoretical minimum risk; plausible minimum risk; (3) feasible minimum risk; and (4) cost-effective minimum risk.[4] The TMREL is the level of risk exposure that minimises risk at the population level or the level of risk that captures the maximum attributable burden. Other possible forms of risk quantification include plausible minimum risk – which reflects the distribution of risk that is conceivably possible and would minimise population-level risk if achieved – whereas feasible minimum risk describes the lowest risk distribution that has been attained within a population, and cost-effective minimum risk is the lowest risk distribution for a population that can be attained in a cost-effective manner. Because no robust set of forecasts for all components of GBD is available, in this study we focus on quantifying attributable burden by using the theoretical minimum risk counterfactual distribution. Given the focus in this study on attributable burden, risk reversibility is not a criterion used in estimation here.

The methods described here provide a high-level overview of the analytical logic. The Methods Appendix to Global burden of 87 risk factors in 204 countries and territories, 1990–2019: a systematic analysis for the Global Burden of Disease Study 2019,[4] upon which this section of the Methods Appendix is based, provides sufficient detail on the methods and overall structure of the estimation process. This study complies with the GATHER recommendations, as documented in the above-mentioned Methods Appendix,[4] proposed by the World Health Organization (WHO) and others, which include recommendations on documentation of data sources, estimation methods, and statistical analysis.

Section 8.2. Steps in calculation of burden

The process of calculation of burden for risk factors passes through several steps, specifically:

1. effect size estimation based on relative risk data obtained from the literature,
2. exposure estimation,
3. determination of the counterfactual level of minimum risk, the TMREL,
4. estimation of the population attributable fraction,

5. estimation of summary exposure value,
6. mediation of the effects of a given risk factor by others, and
7. estimation of the attributable burden

Four key components are included in the estimation of the burden attributable to a given risk factor: the metric of burden being assessed (the number of deaths, YLLs, YLDs, or DALYs [the sum of YLLs and YLDs]); the exposure levels for a risk factor; the RR of a given outcome due to exposure; and the counterfactual level of risk factor exposure. Estimates of attributable burden as DALYs for risk-outcome pairs were generated by using the following model:

$$AB_{jasgt} = \sum_{o=1}^{w} DALY_{joasgt}\, PAF_{joasgt}$$

where AB_{jasgt} is the attributable burden for risk factor j for age group a, sex s, location g, and year t;

$DALY_{joasgt}$ is total DALYs for cause o (of w relevant outcomes for risk factor j) for age group a, sex s, location g, and year t; and PAF_{joasgt} is the PAF for cause o due to risk factor j for age group a, sex s, location g, and year t. The proportions of deaths, YLLs, or YLDs attributable to a given risk factor or riskfactor cluster were analogously computed by sequentially substituting each metric in place of DALYs inthe equation provided.

Section 8.3. Diagrams of steps involved in the estimation of attributable burden

The process is an extremely complicated one, as has been described in further detail.[4] Appendix Figure 5 presents an analytical flowchart of the comparative risk assessment for the estimation of population attributable fractions by geography, age, sex, and year for GBD 2019.

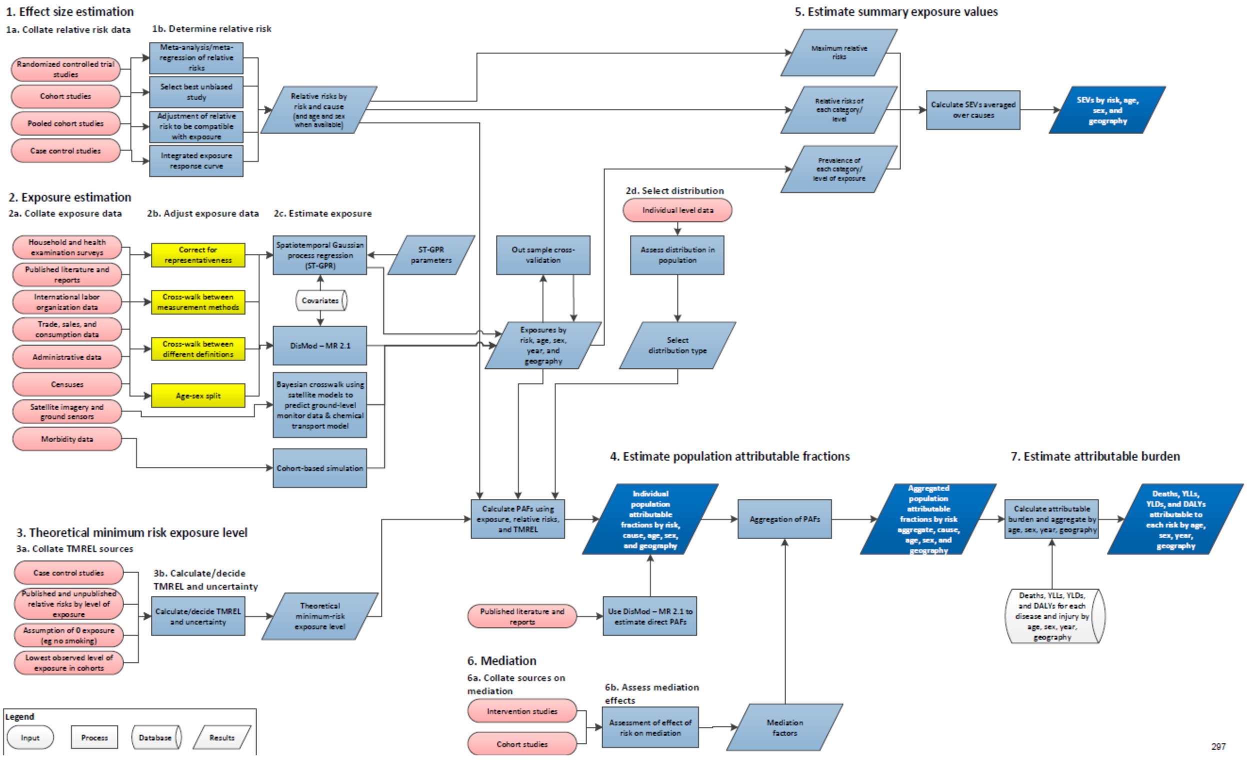

Appendix Figure 5. Analytical flowchart of the comparative risk assessment for the estimation of population attributable fractions. Ovals represent data inputs, rectangular boxes represent analytical steps, cylinders represent databases, and parallelograms represent intermediate and final results. GBD=Global Burden of Disease. SEVs=summary exposure values. TMREL=theoretical minimum-risk exposure level. PAFs=population attributable fractions. YLLs=years of life lost. YLDs=years lived with disability. DALYs=disability-adjusted life-years

Two modelling processes are central to summarising exposure information and other steps in this assessment – DisMod-MR 2.1, described above, and the spatiotemporal Gaussian process regression (ST-GPR).

Appendix Figure 6 presents the spatiotemporal Gaussian process regression (ST-GPR) flowchart

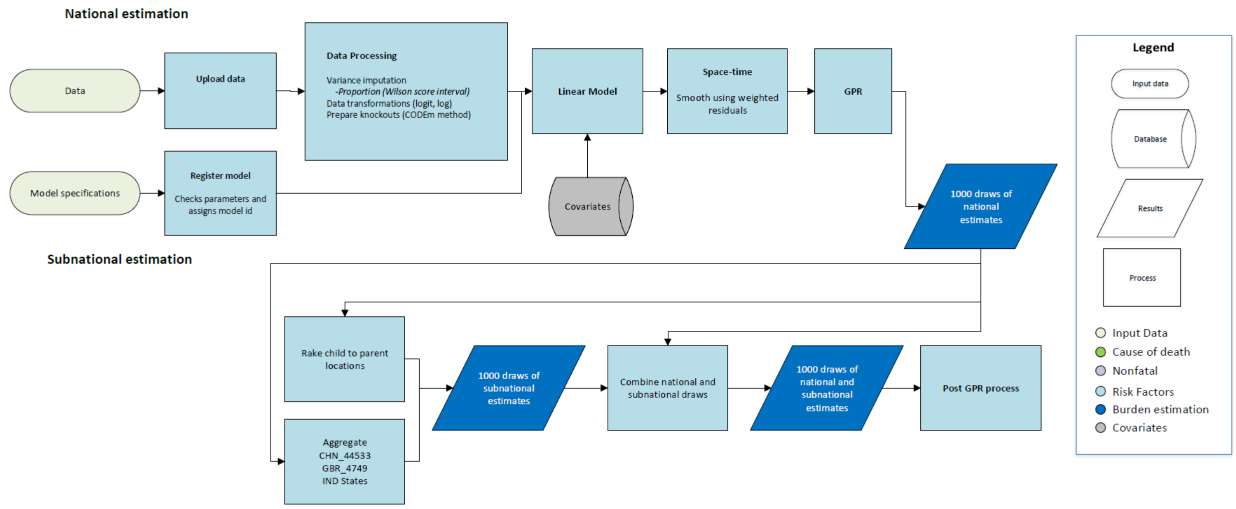

Appendix Figure 6: Spatiotemporal Gaussian process regression (ST-GPR) flowchart

Section 8.4. High fasting plasma glucose

Flowchart

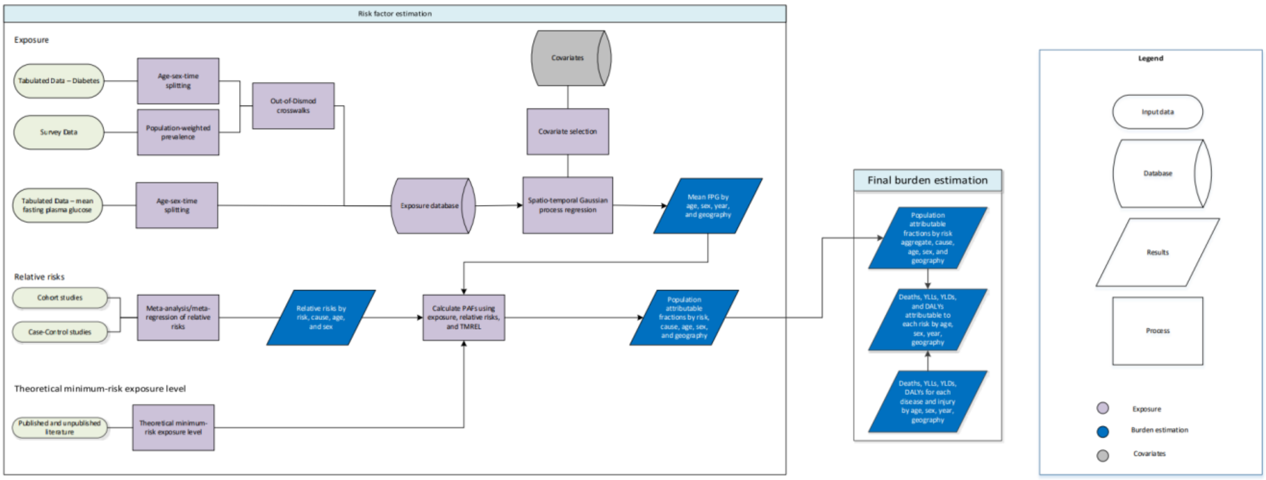

Section 8.4.1. Case definition

High fasting plasma glucose (FPG) is measured as the mean FPG in a population, where FPG is a continuous exposure in units of mmol/L. Since FPG is along a continuum, we define high FPG as any levelabove the TMREL, which is 4.8–5.4 mmol/L.

Section 8.4.2. Data seeking

We conducted a systematic review for FPG and diabetes in GBD 2019. We use all available sources on FPG and prevalence of diabetes in the FPG model.

Search terms:

Diabetes mellitus search string: (diabetes[TI] AND (prevalence[TIAB] OR incidence[TIAB])) OR ('DiabetesMellitus'[MeSH Terms] AND 'epidemiology'[MeSH Terms]) OR (diabetes[TI] AND 'epidemiology'[MeSH Terms]) NOT gestational[All Fields] NOT ('neoplasms'[MeSH Terms] OR 'neoplasms'[All Fields] OR 'cancer'[All Fields]) NOT ('mice'[MeSH Terms] OR 'mice'[All Fields]) NOT ('schizophrenia'[MeSH Terms] OR 'schizophrenia'[All Fields]) NOT ('emigrants and immigrants'[MeSH Terms] OR ('emigrants'[All Fields]AND 'immigrants'[All Fields]) OR 'emigrants and immigrants'[All Fields] OR 'immigrants'[All Fields]) NOT ('pregnancy'[MeSH Terms] OR 'pregnancy'[All Fields] OR 'gestation'[All Fields]) NOT ('rats'[MeSH Terms] OR 'rats'[All Fields] OR 'rat'[All Fields]) NOT ('kidney'[MeSH Terms] OR 'kidney'[All Fields]) NOT renal[All Fields] NOT ('vitamins'[Pharmacological Action] OR 'vitamins'[MeSH Terms] OR 'vitamins'[All Fields] OR 'vitamin'[All Fields])

and

FPG search string: ((“glucose”[Mesh] OR “hyperglycemia”[Mesh] OR “prediabetic state”[Mesh]) AND "Geographic Locations"[Mesh] NOT "United States"[Mesh]) AND ("humans"[Mesh] AND "adult"[MeSH])AND ("Data Collection"[Mesh] OR "Health Services Research"[Mesh] OR "Population Surveillance"[Mesh] OR "Vital statistics"[Mesh] OR "Population"[Mesh] OR "Epidemiology"[Mesh] OR surve*[TiAb]) NOT Comment[ptyp] NOT Case Reports[ptyp]) NOT "hospital"[TiAb]

Section 8.4.3. Data inputs

Data inputs come from three sources:

- Estimates of mean FPG in a representative population
- Individual-level data of fasting plasma glucose measured from surveys
- Estimates of diabetes prevalence in a representative population

Data sources that did not report mean FPG or prevalence of diabetes are excluded from analysis. When a study reported both mean fasting plasma glucose (FPG) and prevalence of diabetes, we use the mean FPG for exposure estimates. Where possible, individual-level data supersede any data described in a study. Individual-level data are aggregated to produce estimates for each five-year age group, sex, location, and year of a survey.

Number of sources (globally) used in exposure and relative risk models in GBD 2019

Measure	Total sources	Countries with data
Total	549	127
Relative risk	20	-
Exposure	529	127

Number of sources (in the Americas) used in exposure and relative risk models in GBD 2019

Location	Source count
Argentina	7
Chile	4
Uruguay	1
Canada	3
USA	10
Barbados	3
Belize	1
Cuba	1
Dominica	1
Dominican Republic	1
Jamaica	5
Suriname	1
Trinidad and Tobago	1
Bolivia	1
Ecuador	4
Peru	6
Colombia	5
Costa Rica	1
El Salvador	1
Guatemala	1

Mexico	7
Venezuela	3
Brazil	8
Paraguay	1
Greenland	2
Puerto Rico	2

Section 8.4.4. Data processing

We perform several processing steps to the data to address sampling and measurement inconsistencies that will ensure the data are comparable.

Small sample size

A sex and age group with a sample size <30 persons is considered a small sample size. In order to avoid small sample size problems that may bias estimates, data are collapsed into the next age group in the same study until the sample size reach at least 30 persons. The intent of collapsing the data is to preserve as much granularity between age groups as possible.If the entire study sample consists of <30 persons and did not include a population weight, the study is excluded from the modelling process.

Crosswalks

We predicted mean FPG from diabetes prevalence using an ensemble distribution. We characterised the distribution of FPG using individual-level data. Details on the ensemble distribution can be found in the next section of this Appendix. Before predicting mean FPG from prevalence of diabetes, we ensured that the prevalence of diabetes was based on the reference case definition: fasting plasma glucose (FPG) >126 mg/dL (7 mmol/L) or on treatment. For more details on how the case-definition crosswalk is conducted, please see the diabetes mellitus appendix in Global, regional, and national incidence, prevalence, and years lived with disability for 354 diseases and injuries for 195 countries, 1990–2019: a systematic analysis for the Global Burden of Disease Study 2019.

Section 8.4.5. Exposure modelling

Exposure estimates are produced for every year between 1980 to 2019 for each national and subnational location, sex, and for each five-year age group starting from 25 years. As in previous rounds ofGBD, we used a spatiotemporal Gaussian process regression (ST-GPR) framework to model the mean fasting plasma glucose at the location, year, age, and sex level. Updates to the ST-GR modelling framework for GBD 2019 are detailed elsewhere in the Appendix.

Fasting plasma glucose is frequently tested or reported in surveys aiming at assessing the prevalence ofdiabetes mellitus. In these surveys, the case definition of diabetes may include both a glucose test and questions about treatment for diabetes. People with positive history of diabetes treatment may be excluded from the FPG test. Thus, the mean FPG in these surveys would not represent the mean FPG in the entire population. In this event, we estimated the prevalence of diabetes assuming a definition of FPG>126 mg/dL (7mmol/L), then crosswalked it to our reference case definition, and then predicted mean FPG.

To inform our estimates in data-sparse countries, we systematically tested a range of covariates and selected age-specific prevalence of obesity as a covariate based on direction of the coefficient and significance level.

Mean FPG is estimated using a mixed-effects linear regression, run separately by sex:

$$\text{logit}(\text{FPG}_{c,a,t}) = \beta_0 + \beta_1 p_{\text{overweight}_{c,a,t}} + \sum_{k=2}^{16} \beta_k I_{A[a]} + \alpha_s + \alpha_r + \alpha_c + \epsilon_{c,a,t}$$

where $p_{\text{overweight}_{c,a,t}}$ is the prevalence of overweight, $I_{A[a]}$ is an indicator variable for a fixed effect on a given five-year age group, and α_s α_r α_c are random effects at the super-region, region, and country level, respectively. The estimates were then propagated through the ST-GPR framework to obtain 1000 drawsfor each location, year, age, and sex.

Section 8.4.6. Theoretical minimum-risk exposure level

The theoretical minimum-risk exposure level (TMREL) for FPG is 4.8–5.4 mmol/L. This was calculated by taking the person-year weighted average of the levels of FPG that were associated with the lowest riskof mortality in the pooled analyses of prospective cohort studies.[1]

Section 8.4.7. Relative risks

We estimate 15 outcomes due to high fasting plasma glucose (continuous risk) or diabetes (categorical risk).

Risk	Outcome
Fasting plasma glucose	Ischaemic heart disease
Fasting plasma glucose	Ischaemic stroke
Fasting plasma glucose	Subarachnoid haemorrhage
Fasting plasma glucose	Intracerebral haemorrhage
Fasting plasma glucose	Peripheral vascular disease
Fasting plasma glucose	Type 1 diabetes
Fasting plasma glucose	Type 2 diabetes
Fasting plasma glucose	CKD due to type 1 diabetes
Fasting plasma glucose	CKD due to type 2 diabetes
Diabetes mellitus	Drug-resistant tuberculosis
Diabetes mellitus	Drug-susceptible tuberculosis
Diabetes mellitus	Multidrug-resistant tuberculosis without extensive drug resistance
Diabetes mellitus	Extensively drug-resistant tuberculosis
Diabetes mellitus	Liver cancer due to NASH

Diabetes mellitus	Liver cancer due to other causes
Diabetes mellitus	Pancreatic cancer
Diabetes mellitus	Ovarian cancer
Diabetes mellitus	Colorectal cancer
Diabetes mellitus	Bladder cancer
Diabetes mellitus	Lung cancer
Diabetes mellitus	Breast cancer
Diabetes mellitus	Glaucoma
Diabetes mellitus	Cataracts
Diabetes mellitus	Dementia

Section 8.4.7.1. Relative risks for high fasting plasma glucose (continuous risk)

After a review of the chronic kidney disease literature, we determined that there is only an attributable risk of chronic kidney disease due to diabetes type 1 and chronic kidney disease due to diabetes type 2 to FPG. Thus, in GBD 2019 we removed chronic kidney disease due to glomerulonephritis, chronic kidney disease due to hypertension, chronic kidney disease due to other causes as an outcome.

Relative risks (RR) were obtained from dose-response meta-analysis of prospective cohort studies. Please see the citation list for a full list of studies that are utilised. For cardiovascular outcomes, we estimated age-specific RRs using DisMod-MR 2.1 with log (RR) as the dependent variable and median age at event as the independent variable with an intercept at age 110. Morbidity and mortality directly caused by diabetes type 1 and diabetes type 2 is considered directly attributable to FPG.

Section 8.4.7.2. Relative risks for diabetes mellitus (categorical risk)

Relative risks were obtained from meta-analysis of cohort studies.

Section 8.5. Risk factors for diabetes

Risks factors for type 2 diabetes are high body-mass index, low physical activity, diet low in fruits, diet low in whole grains, diet low in nuts and seeds, diet high in red meat, diet high in processed meat, diet high in sweetened beverages, alcohol use, smoking and secondhand smoke, ambient particulate matter, household air pollution, and non-optimal temperature. Exposure information for air pollution was obtained from several sources, including satellite data.

Methodological details of these risk factors are to be found in the Methods Appendix (Supplementary Appendix 1) of the 2019 GBD article: Global burden of 87 risk factors in 204 countries and territories, 1990–2019: a systematic analysis for the Global Burden of Disease Study 2019. *Lancet* 2020; **396:** 1223–49.[4]

Section 9. Socio-demographic Index

We used the GBD Socio-demographic Index (SDI)[2] groups to explore the difference in mortality rates between countries with different levels of development. The SDI is a composite indicator of development status strongly correlated with health outcomes. In short, it is the geometric mean of 0

to 1 indices of total fertility rate in those under 25 years old, mean education for those age 15 years or older, and lag-distributed income per capita (LDI). An index score of 0 represents the minimum level of each covariate input past which selected health outcomes can get no worse, and an index score of 1 represents the maximum level of each covariate input past which selected health outcomes cease to improve. As a composite, a location with an SDI of 0 would have a theoretical minimum level of development relevant to these health outcomes, and a location with an SDI of 1 would have a theoretical maximum level of development relevant to these health outcomes. Detailed information about SDI calculation and the SDI values for each country has been described elsewhere.[2]

Section 10. Universal Health Coverage (UHC) Effective Care Index

As applied in this analysis and explained in greater detail elsewhere,[3] the UHC effective coverage measurement framework involves 30 unique cells from a matrix of five health service types—promotion, prevention, treatment, rehabilitation, and palliation— against five population-age groups (reproductive and newborn, children younger than 5 years, children and adolescents aged 5–19 years, adults aged 20–64 years, and older adults aged ≥65 years). Treatment is sub-divided into two separate groups: first, communicable diseases and maternal, newborn, and child health; and second, non-communicable diseases. Effective coverage indicators were then mapped to these cells to represent needed health services across the life course. 23 effective coverage indicators were included in the present analysis. Data for directly measuring effective intervention coverage are rarely available across health services, locations, and over time. Subsequently, we used viable proxy measures and analytical techniques to approximate effective coverage for conditions considered amenable to health care. Criteria set forth by the WHO 13th General Work Program (GPW13) Expert Reference Group guided selection of effective coverage indicators and preferred measurement approaches. Such criteria stipulated that effective coverage indicators should be currently measurable (ie, data and methods that support indicator measurement today); reflect differences in effective health services and not factors outside the immediate scope of health systems and UHC (eg, tobacco taxation and physical infrastructure such as roads and water systems); and use indicators already encompassed within the Sustainable Development Goals (SDGs) and GPW13, or draw from data systems required for monitoring of SDGs and GPW13.

Four effective coverage indicators were measures of intervention coverage, and 19 were mortality-based measures to proxy access to quality of care. For the mortality-based measures, we primarily used mortality-to-incidence ratios (MIRs) and mortality-to-prevalence ratios (MPRs) for chronic or longer-term conditions (eg, diabetes or asthma).

Effective coverage indicators for intervention coverage were kept on their natural scale (0–100%), whereas the 19 other effective coverage indicators were transformed to values on a 0–100 scale. Across locations and from 1990 to 2019, 0 was set by values at the 97·5th percentile or higher (ie, "worst" levels of MIRs) and 100 by the 2·5th percentile or lower (ie, "best" levels of MIRs).

Population-level measures of effective coverage should represent the fraction of total health gains a health system could potentially provide, given currently available interventions that a health system actually delivers. This construct is thus grounded in the principle of comparability—all health systems ought to maximise potential health gains for their populations—but also requires accounting for local health needs and epidemiological profiles. For instance, if a country currently experiences a high burden of diabetes and a comparatively lower burden of HIV, at least equal or

even higher priority in expanding services for diabetes should occur relative to HIV in order to further support health gains.

To construct the UHC effective coverage index, we weighted each effective coverage indicator relative to their health gain weights, a metric approximating the population health gains potentially deliverable by health systems for each location-year. In brief, calculations were based on three inputs for each effective coverage indicator and corresponding population-age group: estimates on the 0–100 scale, targeted disease burden, and effectiveness categories of associated interventions or services. For effectiveness, incremental values were assumed by category (ie, 90% effectiveness for category 1, 70% for category 2, 50% for category 3, and so on).

GATHER checklist

Checklist of information that should be included in new reports of global health estimates

Item #	Checklist item	Reported on page #
**Objectives and funding**		
**1**	Define the indicator(s), populations (including age, sex, and geographic entities), and time period(s) for which estimates were made.	5, 8
**2**	List the funding sources for the work.	9
**Data Inputs**		
For all data inputs from multiple sources that are synthesized as part of the study:		
**3**	Describe how the data were identified and how the data were accessed.	6, 7,<br><br>Methods appendix section 4.1, section 6.1.1.1,<br>Section 6.1.2.1,<br>Section 6.2.1,<br>Section 7.3<br>Section 8.4
**4**	Specify the inclusion and exclusion criteria. Identify all ad-hoc exclusions.	6, 7
**5**	Provide information on all included data sources and their main characteristics. For each data source used, report reference information or contact name/institution, population represented, data collection method, year(s) of data collection, sex and age range, diagnostic criteria or measurement method, and sample size, as relevant.	Methods appendix section 4.1, section 6.1.1.1,<br>Section 6.1.2.1,<br>Section 6.2.1,<br>Section 7.3<br>Section 8.4<br><br>http://ghdx.healthdata.org/gbd-2019
**6**	Identify and describe any categories of input data that have potentially important biases (e.g., based on characteristics listed in item 5).	Methods appendix section 4.1, section 6.1.1.1,<br>Section 6.1.2.1,<br>Section 6.2.1,<br>Section 7.3<br>Section 8.4
For data inputs that contribute to the analysis but were not synthesized as part of the study:		
**7**	Describe and give sources for any other data inputs.	http://ghdx.healthdata.org/gbd-2019
For all data inputs:		
**8**	Provide all data inputs in a file format from which data can be efficiently extracted (e.g., a spreadsheet rather than a PDF), including all relevant meta-data listed in item 5. For any data inputs that cannot be shared because of ethical or legal reasons, such as third-party ownership, provide a contact	http://ghdx.healthdata.org

	name or the name of the institution that retains the right to the data.	https://vizhub.healthdata.org/gbd-compare/
**Data analysis**		
**9**	Provide a conceptual overview of the data analysis method. A diagram may be helpful.	5-9<br><br>Methods appendix section 6.1, Section 6.2, Section 7.3 Section 8.4
**10**	Provide a detailed description of all steps of the analysis, including mathematical formulae. This description should cover, as relevant, data cleaning, data pre-processing, data adjustments and weighting of data sources, and mathematical or statistical model(s).	Methods appendix Section 5, section 6.1, Section 6.2, Section 7 Section 8
**11**	Describe how candidate models were evaluated and how the final model(s) were selected.	Methods appendix section 6.1, Section 6.2, Section 7.3 Section 8.4
**12**	Provide the results of an evaluation of model performance, if done, as well as the results of any relevant sensitivity analysis.	Methods appendix section 6.1, Section 6.2, Section 7.3 Section 8.4
**13**	Describe methods for calculating uncertainty of the estimates. State which sources of uncertainty were, and were not, accounted for in the uncertainty analysis.	9<br><br>Methods appendix Section 5.1 Section 7.1.5 Section 7.2
**14**	State how analytic or statistical source code used to generate estimates can be accessed.	Code is provided in an online repository https://github.com/ihmeuw/ihme-modeling/tree/main/gbd_2019

Results and Discussion		
**15**	Provide published estimates in a file format from which data can be efficiently extracted.	Main text, methods appendix, and online data tools (data visualization tools, data query tools, and the Global Health Data Exchange, http://ghdx.healthdata.org
**16**	Report a quantitative measure of the uncertainty of the estimates (e.g. uncertainty intervals).	9-13
**17**	Interpret results in light of existing evidence. If updating a previous set of estimates, describe the reasons for changes in estimates.	13-17
**18**	Discuss limitations of the estimates. Include a discussion of any modelling assumptions or data limitations that affect interpretation of the estimates.	17-18

This checklist should be used in conjunction with the GATHER statement and Explanation and Elaboration document, found on gather-statement.org

Authors' Contributions

Managing the overall research enterprise

Ewerton Cousin, Maria Inês Schmidt, and Bruce Duncan.

Writing the first draft of the manuscript

Ewerton Cousin Maria Inês Schmidt, and Bruce Duncan.

Primary responsibility for applying analytical methods to produce estimates

Ewerton Cousin and Kanyin Liane Ong.

Primary responsibility for seeking, cataloguing, extracting, or cleaning data; designing or coding figures and tables

Ewerton Cousin

Providing data or critical feedback on data sources

Ewerton Cousin, Maria Inês Schmidt, Abdelrahman I Abushouk, Marcela Agudelo-Botero, Jacqueline Elizabeth Alcalde-Rabanal, Nelson Alvis-Guzman, Nelson J Alvis-Zakzuk, Till Winfried Bärnighausen, Sanjay Basu, Vijay Kumar Chattu, Michael H Criqui, Samath Dhamminda Dharmaratne, Mohamed M Gad, Shakiba Ghasemi Assl, Rajat Das Gupta, Nima Hafezi-Nejad, Tanvir Kahlon, Jagdish Khubchandani, Adnan Kisa, Barthelemy Kuate Defo, Ali H Mokdad, Andrew T Olagunju, Leonardo Roever, Seyedmohammad Saadatagah, Itamar S Santos, Sara Sheikhbahaei, Jasvinder A Singh, Marcos Roberto Tovani-Palone, Diana Zuleika Velazquez, and Bruce B Duncan.

Developing methods or computational machinery

Ashkan Afshin, Adnan Kisa, and Ali H Mokdad.

Providing critical feedback on methods or results

Rafael Lozano, Ashkan Afshin, Abdelrahman I Abushouk, Gina Agarwal, Marcela Agudelo-Botero, Ziyad Al-Aly, Jacqueline Elizabeth Alcalde-Rabanal, Nelson Alvis-Guzman, Nelson J Alvis-Zakzuk, Benny Antony, Till Winfried Bärnighausen, Sanjay Basu, Arielle Wilder Bell, Isabela M Bensenor, Zahid A Butt, Ismael R Campos-Nonato, Vijay Kumar Chattu, Michael H Criqui, Samath Dhamminda Dharmaratne, Daniel Diaz, Irina Filip, Mohamed M Gad, Shakiba Ghasemi Assl, Sameer Vali Gopalani, Rajat Das Gupta, Nima Hafezi-Nejad, Maryam Hashemian, Simon I Hay, Tanvir Kahlon, Jagdish Khubchandani, Ruth W Kimokoti, Adnan Kisa, Barthelemy Kuate Defo, Ted R Miller, Ali H Mokdad, Linda Morales, Shane Douglas Morrison, Yeshambel T Nigatu, Andrew T Olagunju, Seithikurippu R Pandi-Perumal, Urvish K Patel, Amir Radfar, Maria Rios-Blancas, Leonardo Roever, Seyedmohammad Saadatagah, Juan Sanabria, Thirunavukkarasu Sathish, Mahsima Shabani, Omid Shafaat, Sara Sheikhbahaei, Diego Augusto Santos Silva, Ambrish Singh, Jasvinder A Singh, Marcos Roberto Tovani-Palone, Diana Zuleika Velazquez, and Siddhesh Zadey.

Drafting the work or revising is critically for important intellectual content

Ewerton Cousin, Maria Inês Schmidt, Abdelrahman I Abushouk, Gina Agarwal, Jacqueline Elizabeth Alcalde-Rabanal, Nelson Alvis-Guzman, Nelson J Alvis-Zakzuk, Malke Asaad, Till Winfried Bärnighausen, Sanjay Basu, Arielle Wilder Bell, Isabela M Bensenor, Vijay Kumar Chattu, Michael H Criqui, Claudio Alberto Dávila-Cervantes, Edgar Denova-Gutiérrez, Samath Dhamminda Dharmaratne, Daniel Diaz, Irina Filip, Mohamed M Gad, MA Garcia-Gordillo, Shakiba Ghasemi Assl, Sameer Vali Gopalani, Rafael Alves

Guimarães, Rajat Das Gupta, Nima Hafezi-Nejad, Simon I Hay, Tanvir Kahlon, Jagdish Khubchandani, Adnan Kisa, Barthelemy Kuate Defo, Iván Landires, Ted R Miller, Ali H Mokdad, Virginia Nuñez-Samudio, Andrew T Olagunju, Urvish K Patel, Amir Radfar, Leonardo Roever, Juan Sanabria, Itamar S Santos, Thirunavukkarasu Sathish, Mahsima Shabani, Omid Shafaat, Sara Sheikhbahaei, Diego Augusto Santos Silva, Jasvinder A Singh, Marcos Roberto Tovani-Palone, Diana Zuleika Velazquez, and Bruce B Duncan.

Managing the estimation or publications process

Ashkan Afshin, Simon I Hay, and Ali H Mokdad.

References

1 Vos T, Lim SS, Abbafati C, *et al.* Global burden of 369 diseases and injuries in 204 countries and territories, 1990–2019: a systematic analysis for the Global Burden of Disease Study 2019. *The Lancet* 2020; **396**: 1204–22.

2 Wang H, Abbas KM, Abbasifard M, *et al.* Global age-sex-specific fertility, mortality, healthy life expectancy (HALE), and population estimates in 204 countries and territories, 1950–2019: a comprehensive demographic analysis for the Global Burden of Disease Study 2019. *The Lancet* 2020; **396**: 1160–203.

3 Lozano R, Fullman N, Mumford JE, *et al.* Measuring universal health coverage based on an index of effective coverage of health services in 204 countries and territories, 1990–2019: a systematic analysis for the Global Burden of Disease Study 2019. *The Lancet* 2020; **396**: 1250–84.

4 Murray CJ, Aravkin AY, Zheng P, *et al.* Global burden of 87 risk factors in 204 countries and territories, 1990–2019: a systematic analysis for the Global Burden of Disease Study 2019. *The Lancet* 2020; **396**: 1223–49.

5 Stevens GA, Alkema L, Black RE, *et al.* Guidelines for accurate and transparent health estimates reporting: the GATHER statement. *PLoS Med* 2016; **13**: e1002056.

6 GBD 2017 Risk Factor Collaborators. Global, regional, and national comparative risk assessment of 84 behavioural, environmental and occupational, and metabolic risks or clusters of risks for 195 countries and territories, 1990-2017: a systematic analysis for the Global Burden of Disease Study 2017. *Lancet Lond Engl* 2018; **392**: 1923–94.

7 Dicker D, Nguyen G, Abate D, *et al.* Global, regional, and national age-sex-specific mortality and life expectancy, 1950–2017: a systematic analysis for the Global Burden of Disease Study 2017. *The lancet* 2018; **392**: 1684–735.

8 Johnson SC, Cunningham M, Dippenaar IN, *et al.* Public health utility of cause of death data: applying empirical algorithms to improve data quality. *BMC Med Inform Decis Mak* 2021; **21**: 1–20.

9 Lozano R, Naghavi M, Foreman K, *et al.* Global and regional mortality from 235 causes of death for 20 age groups in 1990 and 2010: a systematic analysis for the Global Burden of Disease Study 2010. *The lancet* 2012; **380**: 2095–128.

10 Foreman KJ, Lozano R, Lopez AD, Murray CJ. Modeling causes of death: an integrated approach using CODEm. *Popul Health Metr* 2012; **10**: 1–23.

11 Bell RM, Koren Y. Lessons from the netflix prize challenge. *Acm Sigkdd Explor Newsl* 2007; **9**: 75–9.

12 Bell RM, Koren Y, Volinsky C. All together now: A perspective on the netflix prize. *Chance* 2010; **23**: 24–9.

13 Murray CJ, Lopez AD. Global mortality, disability, and the contribution of risk factors: Global Burden of Disease Study. *The lancet* 1997; **349**: 1436–42.

Supplementary tables and figures

List of Supplementary Tables and Figures

Supplementary Table 1. List of regions and countries of the Americas

Supplementary Table 2. Number of data sources and sample sizes (in thousands) by region and year

Supplementary Table 3. Death counts and percentage of all-cause deaths due to diabetes. The Americas, regions, and countries.

Supplementary Table 4. Age-standardised DALYs due to diabetes in the Americas by region and location: 2019 values, percentage change from 1990 to 2019, percentage of total DALYs, and percentage due to type 2 diabetes

Supplementary Table 5. All-age DALYs rates per 100,000 in 2019 and percentage change of DALYs due to diabetes by type.

Supplementary Table 6. Country-specific age-standardized DALYs rate in 2019 and percentage change 1990-2019, by type of diabetes.

Supplementary Table 7. Percentage of DALYs due to years of life lost (YLLs) and years lived with disability (YLDs) by type of diabetes for the Americas and by region, 1990 and 2019.

Supplementary Table 8. All-age high fasting plasma glucose DALYs due to specific causes in 2019 by the Americas regions.

Supplementary Table 9. Correlation of the SDI, the HAQ Index, and prevalence of diabetes with the age-standardised DALY rate due to diabetes in the Americas, 2019.

Supplementary Table 10. Diabetes prevalence and incidence rates (95% Uncertainty Intervals) in adults in the Americas in 1990 and 2019 and their change from 1990 to 2019 (95% Uncertainty Interval).

Supplementary Table 11. Prevalent and incident cases of diabetes globally, in the Americas and by region in 2019.

Supplementary Table 12. Crude population attributable fraction of DALYs due to type 2 diabetes for the main risk factors for diabetes identified by GBD 2019, by region.

Supplementary Figure 1: Correlation of the Socio-demographic Index (Panel A) and the Healthcare Access and Quality Index (Panel B) with DALYs due to type 1 diabetes.

Supplementary Figure 2: Changes in the age-standardised YLLs rate and age-standardised YLDs rate in regions of the Americas over time, from 1990 to 2019. Each point represents both rates for a given year for the regions shown. The years in each series generally advance from left to right.

Supplementary Figure 3: Age-standardised prevalence of type 2 diabetes across the range of the summary exposure value (SEV) of body-mass index (BMI) in the Americas, 2019.

Supplementary Table 1. List of regions and countries of the Americas.	
**High-Income North America**	**Caribbean**
Canada	Antigua and Barbuda
Greenland	Bahamas
United States of America	Barbados
**Central Latin America**	Belize
Colombia	Bermuda
Costa Rica	Cuba
El Salvador	Dominica
Guatemala	Dominican Republic
Honduras	Grenada
Mexico	Guyana
Nicaragua	Haiti
Panama	Jamaica
Venezuela	Saint Lucia
	Saint Vincent and the Grenadines
**Andean Latin America**	Suriname
Ecuador	Trinidad and Tobago
Peru	Saint Kitts and Nevis
Bolivia	United States Virgin Islands
**Southern Latin America**	Puerto Rico
Argentina	
Chile	**Tropical Latin America**
Uruguay	Brazil
	Paraguay

Supplementary Table 2 - Number of data sources and sample sizes (in thousands) by region and year.						
**Region**	**1990-1994**	**1995-1999**	**2000-2004**	**2005-2009**	**2010-2014**	**2015-2019**
**Non-fatal**						
**Overall Diabetes***						
Andean Latin America	1 (4,000)	1 (5.1)	0	6 (27.6)	5 (46.1)	1 (0.2)
Caribbean+	4 (17,66.9)	4 (13.5)	2 (2.6)	10 (340.6)	8 (1,152.2)	0
Central Latin America	5 (33,234.8)	2 (27.9)	6 (38.8)	12 (78.6)	9 (93,022.9)	3 (12.2)
High-Income North America+	7 (10,599.1)	7 (15,569.5)	11 (9,309.3)	6 (9,211.1)	18 (1,246.9)	1 (5.3)
Southern Latin America+	5 (34,937.0)	3 (6,361.3)	3 (3,146.3)	5 (19.1)	5 (64.5)	0
Tropical Latin America+	3 (9,713.9)	5 (473.5)	11 (535.4)	7 (364.9)	3 (28.3)	0
**Type 1 diabetes only**						
Andean Latin America	1 (4,000.0)	0	0	0	0	0
Caribbean	1 (17,169.0)	0	0	0	1 (326.8)	0
Central Latin America	1 (33,168.8)	0	0	0	0	0
High-Income North America+	4 (4,035.8)	4 (6,735.8)	5 (3,597.5)	1 (826.7)	11 (607.1)	0
Southern Latin America+	4 (24,107.2)	1	1	0	0	0
Tropical Latin America	2 (9,444.3)	1 (231.5)	1 (253.1)	1 (180.5)	0	0
**High Fasting Plasma Glucose**						
Andean Latin America	0	1 (5.1)	2 (53.3)	2 (3.3)	4 (39.9)	0
Caribbean+	2 (8.1)	3 (1.5)	1 (35.7)	7 (166.8)	3 (57.6)	0
Central Latin America+	1 (10.7)	2 (1,305.5)	6 (114.3)	4 (43,145.0)	2 (120.9)	0
High-Income North America	1 (14.2)	2 (45.4)	3 (14.9)	7 (108.2)	2 (4.7)	0
Southern Latin America	1 (0.1)	2 (17.3)	3 (71.3)	2 (2.0)	1 (100.0)	0
Tropical Latin America	1 (1.5)	2 (41.9)	2 (3.4)	3 (8.6)	1 (1.3)	0

*These surveys do not distinguish the type of diabetes.
** We chose to use end year of each source as the metric for determining which 5-year-bin a source was classified as.
+Some sources did not include sample size.

**Supplementary Table 3 - Death counts and percentage of all-cause deaths due to diabetes. The Americas, regions, and countries.**		
**Location**	**Death count** N (95% UI)	**Percent of all deaths** % (95% UI)
**The Americas**	**409000 (373000 to 443000)**	**5.9% (5.4% to 6.2%)**
High-income North America	121000 (109000 to 132000)	3.8% (3.4% to 4.1%)
Canada	8920 (7830 to 9880)	3.1% (2.7% to 3.5%)
Greenland	7.47 (5.98 to 8.99)	1.6% (1.4% to 1.8%)
USA	113000 (101000 to 122000)	3.9% (3.5% to 4.2%)
Andean Latin America	19600 (16200 to 23400)	6.8% (6.2% to 7.4%)
Bolivia	5270 (4160 to 6580)	8.2% (7.1% to 9.4%)
Ecuador	7170 (5730 to 9070)	8.5% (7.7% to 9.2%)
Peru	7170 (5370 to 9330)	5.2% (4.5% to 5.7%)
Caribbean	23500 (19900 to 27800)	6.9% (6.3% to 7.6%)
Antigua and Barbuda	69.1 (60.1 to 78.8)	11.6% (10.8% to 12.4%)
The Bahamas	184 (153 to 222)	7% (6.4% to 7.5%)
Barbados	354 (297 to 410)	11.7% (10.7% to 12.5%)
Belize	191 (168 to 217)	10.5% (9.9% to 11.2%)
Bermuda	30.6 (25.7 to 36.8)	5.3% (4.8% to 5.8%)
Cuba	2870 (2340 to 3510)	2.7% (2.5% to 3%)
Dominica	72 (60.2 to 86)	10.2% (9.3% to 11.1%)
Dominican Republic	3640 (2790 to 4660)	5.7% (5% to 6.4%)
Grenada	92.6 (84.2 to 101)	11.7% (11% to 12.5%)
Guyana	665 (527 to 830)	10.7% (10% to 11.4%)
Haiti	4930 (3480 to 7060)	6.8% (5.4% to 8.8%)
Jamaica	2730 (2230 to 3280)	14.4% (13.2% to 15.3%)
Puerto Rico	4090 (3210 to 5090)	12.5% (11.2% to 13.5%)
Saint Kitts and Nevis	43.8 (36.8 to 51.1)	9.1% (8% to 9.9%)
Saint Lucia	154 (131 to 181)	11.3% (10.4% to 12.3%)
Saint Vincent and the Grenadines	124 (109 to 141)	12.5% (11.6% to 13.3%)
Suriname	357 (300 to 422)	8.8% (8.1% to 9.6%)
Trinidad and Tobago	2040 (1570 to 2600)	17.8% (16.7% to 18.9%)
Virgin Islands	98.1 (84.1 to 112)	7.8% (7.1% to 8.5%)
Central Latin America	142000 (125000 to 161000)	10.7% (9.8% to 11.4%)
Colombia	10000 (7780 to 12600)	4.3% (3.8% to 4.8%)
Costa Rica	1020 (796 to 1330)	4.3% (3.8% to 4.9%)
El Salvador	3690 (2800 to 4710)	9.7% (8.7% to 10.6%)
Guatemala	8780 (6970 to 10900)	10.7% (10.1% to 11.4%)
Honduras	2260 (1800 to 2860)	4.8% (4% to 5.7%)
Mexico	97100 (83600 to 111000)	14% (13.1% to 14.8%)
Nicaragua	3120 (2580 to 3710)	11.8% (10.7% to 12.8%)
Panama	1610 (1270 to 2040)	8.8% (7.8% to 9.6%)
Venezuela	14500 (11200 to 18800)	8.3% (7.6% to 8.9%)
Tropical Latin America	80500 (73600 to 85400)	6% (5.4% to 6.3%)

Brazil	77400 (70800 to 82100)	5.9% (5.3% to 6.2%)
Paraguay	3100 (2430 to 3920)	9.8% (8.7% to 10.6%)
Southern Latin America	22500 (20400 to 24500)	4.7% (4.2% to 5.1%)
Argentina	15900 (14400 to 17500)	4.7% (4.3% to 5.2%)
Chile	5360 (4760 to 5920)	4.8% (4.3% to 5.3%)
Uruguay	1200 (1090 to 1310)	3.6% (3.3% to 3.9%)

Supplementary Table 4. Age-standardised DALYs per 100,000 due to diabetes in the Americas by location: 2019 values, percentage change from 1990 to 2019, percentage of total DALYs, and percentage due to type 2 diabetes.

	All-ages DALYs 2019						Age-standardised DALYs					
	Rate (per 100,000)			Percentage type 2 diabetes			2019 rate (per 100,000)			Percentage change 1990–2019		
		95% uncertainty interval			95% uncertainty interval			95% uncertainty interval			95% uncertainty interval	
**Location**	**Value**	**Lower**	**Upper**	**Value**	**Lower**	**Upper**	**Value**	**Lower**	**Upper**	**Value**	**Lower**	**Upper**
**Global**	**1,608**	**1,374**	**1,881**	**91.5%**	**89.9%**	**92.9%**	**1,589**	**1,359**	**1,856**	**23.1%**	**16.9%**	**28.5%**
**The Americas**	**2,266**	**1,930**	**2,649**	**92.6%**	**91.3%**	**93.8%**	**2,036**	**1,732**	**2,385**	**26.2%**	**20.8%**	**31.3%**
High-income North America	2,016	1,656	2,430	92.0%	90.8%	93.1%	1,513	1,238	1,829	31.2%	25.9%	36.1%
Canada	1,192	951	1,460	86.6%	84.2%	88.6%	838	667	1,030	16.0%	5.7%	26.0%
Greenland	998	795	1,241	90.8%	89.0%	92.3%	919	733	1,136	0.9%	-16.4%	18.1%
USA	2,112	1,736	2,549	92.3%	91.2%	93.4%	1,594	1,307	1,928	32.7%	27.5%	37.8%
Southern Latin America	1,648	1,395	1,942	92.5%	90.9%	93.8%	1,489	1,258	1,760	15.6%	8.3%	22.9%
Argentina	1,702	1,453	1,989	91.7%	90.0%	93.2%	1,575	1,344	1,840	13.4%	6.2%	20.9%
Chile	1,567	1,266	1,910	94.5%	93.3%	95.7%	1,385	1,120	1,688	22.4%	12.3%	31.8%
Uruguay	1,423	1,212	1,664	91.4%	90.0%	92.7%	1,094	929	1,284	27.6%	15.9%	40.2%
Andean Latin America	1,634	1,360	1,931	92.3%	90.4%	93.9%	1,825	1,523	2,153	32.7%	15.4%	50.2%
Bolivia	2,257	1,821	2,770	92.2%	89.9%	94.0%	2,794	2,263	3,425	18.0%	-3.2%	45.7%
Ecuador	2,142	1,751	2,623	92.8%	90.8%	94.5%	2,427	1,987	2,962	64.0%	39.8%	94.4%
Peru	1,184	944	1,453	92.0%	90.0%	93.5%	1,277	1,017	1,566	20.3%	-1.2%	47.2%
Caribbean	2,901	2,423	3,472	92.5%	90.8%	94.0%	2,814	2,353	3,366	7.0%	-4.5%	18.7%
Antigua and Barbuda	3,664	3,083	4,291	92.7%	90.7%	94.1%	3,660	3,093	4,276	11.3%	-0.5%	23.2%
Bahamas	2,922	2,381	3,553	90.8%	88.1%	92.8%	3,034	2,481	3,685	1.5%	-12.7%	15.9%
Barbados	4,490	3,737	5,296	94.5%	92.9%	95.5%	3,438	2,864	4,053	0.8%	-11.6%	12.6%
Belize	2,836	2,448	3,278	89.9%	87.4%	91.8%	3,650	3,163	4,199	51.5%	36.0%	68.7%
Bermuda	2,230	1,812	2,733	94.1%	92.6%	95.2%	1,525	1,239	1,862	-16.8%	-26.7%	-6.4%
Cuba	2,094	1,593	2,683	95.8%	94.3%	96.8%	1,637	1,246	2,098	-7.2%	-17.6%	2.7%
Dominica	4,810	4,001	5,758	93.4%	91.6%	94.8%	4,202	3,496	5,029	14.9%	-1.2%	32.4%
Dominican Republic	1,968	1,569	2,423	89.9%	87.7%	91.9%	2,209	1,765	2,715	58.7%	30.4%	91.8%
Grenada	4,414	3,821	5,099	93.2%	91.1%	94.6%	4,459	3,873	5,140	14.0%	3.1%	24.3%
Guyana	5,052	4,106	6,114	91.8%	89.5%	93.5%	5,840	4,769	7,034	25.0%	2.5%	51.0%

Haiti	2,935	2,233	3,867	88.7%	86.1%	91.5%	4,154	3,190	5,442	-8.6%	-25.4%	12.8%
Jamaica	4,014	3,298	4,802	94.4%	92.4%	95.6%	4,146	3,404	4,957	31.4%	13.0%	51.7%
Puerto Rico	4,741	3,845	5,769	93.7%	92.1%	95.1%	3,260	2,646	3,977	12.1%	-2.6%	28.6%
Saint Kitts and Nevis	3,816	3,119	4,525	92.4%	89.4%	94.9%	3,805	3,156	4,459	-9.3%	-21.7%	4.0%
Saint Lucia	4,416	3,672	5,247	94.0%	92.2%	95.3%	4,193	3,483	4,973	-9.4%	-19.3%	0.7%
Saint Vincent and the Grenadines	5,206	4,511	6,043	93.7%	91.9%	94.9%	4,826	4,191	5,602	10.2%	-1.6%	22.4%
Suriname	3,919	3,256	4,695	93.2%	91.1%	94.8%	3,850	3,213	4,582	44.5%	29.1%	62.3%
Trinidad and Tobago	6,347	5,067	7,889	95.6%	94.2%	96.5%	5,519	4,400	6,847	-11.5%	-27.9%	6.7%
Virgin Islands	4,478	3,777	5,351	94.2%	92.7%	95.3%	3,124	2,618	3,732	25.7%	9.3%	43.3%
Central Latin America	3,267	2,823	3,806	93.3%	91.3%	94.9%	3,485	3,017	4,059	20.4%	11.3%	29.7%
Colombia	1,676	1,283	2,113	95.7%	94.2%	96.7%	1,647	1,261	2,075	-7.7%	-18.7%	3.3%
Costa Rica	1,774	1,375	2,250	94.4%	92.1%	96.1%	1,786	1,386	2,262	16.8%	4.2%	30.3%
El Salvador	3,231	2,588	3,968	90.0%	86.4%	92.6%	3,458	2,764	4,250	118.7%	79.7%	163.9%
Guatemala	3,217	2,637	3,870	91.0%	88.7%	93.2%	4,357	3,579	5,237	162.0%	119.4%	215.1%
Honduras	1,998	1,592	2,488	91.1%	87.2%	94.0%	2,665	2,138	3,298	58.0%	41.8%	79.3%
Mexico	4,195	3,598	4,858	93.3%	91.3%	94.9%	4,505	3,874	5,214	15.7%	4.7%	26.4%
Nicaragua	2,869	2,371	3,481	91.1%	87.7%	93.8%	3,788	3,134	4,535	60.7%	41.4%	83.1%
Panama	2,387	1,909	2,929	94.4%	92.5%	95.8%	2,425	1,939	2,980	58.8%	39.5%	81.8%
Venezuela	2,880	2,314	3,536	94.1%	91.9%	95.7%	2,886	2,326	3,542	32.4%	12.3%	57.1%
Tropical Latin America	1,886	1,625	2,175	92.9%	91.7%	94.0%	1,914	1,651	2,205	-5.6%	-9.6%	-2.1%
Brazil	1,875	1,613	2,160	92.9%	91.7%	94.0%	1,895	1,632	2,179	-7.2%	-11.2%	-3.6%
Paraguay	2,251	1,798	2,728	93.1%	91.3%	94.5%	2,723	2,181	3,296	88.3%	56.4%	129.2%

Supplementary Table 5 - All-age DALYs rates per 100,000 in 2019 and percentage change of DALYs due to diabetes by type.

Location	All-age DALYs 2019<br>Rate (95% UI)	Change 1990-2019<br>% (95% UI)
**Type 1 diabetes**		
**Global**	**136 (111.5 to 162)**	**1.96% (-8.14% to 12.41%)**
**The Americas**	**166.4 (134.6 to 198.7)**	**21.44% (9.38% to 33.18%)**
High-income North America	161.4 (129.6 to 199.6)	17.37% (5.53% to 25.59%)
Central Latin America	218.4 (163.3 to 286)	67.69% (38.95% to 98.02%)
Caribbean	217.3 (170.3 to 272.4)	5.82% (-13.09% to 27.62%)
Andean Latin America	125.4 (93.2 to 164.2)	16.86% (-5.23% to 42.2%)
Tropical Latin America	134.1 (108 to 159.2)	-0.79% (-12.74% to 9.26%)
Southern Latin America	123.9 (99.1 to 150.9)	-5.41% (-13.17% to 3%)
**Type 2 diabetes**		
**Global**	**1472.3 (1247.7 to 1737.7)**	**54.04% (46.92% to 60.75%)**
**The Americas**	**2099.5 (1779.7 to 2472.7)**	**59.22% (52.75% to 65.52%)**
High-income North America	1855 (1518.6 to 2253.2)	69.75% (62.37% to 77.02%)
Central Latin America	3048.7 (2614.2 to 3579.7)	64.57% (52.39% to 76.87%)
Caribbean	2684 (2232 to 3218.7)	34.39% (20.29% to 48.81%)
Andean Latin America	1509 (1263.9 to 1791.8)	73.89% (51.75% to 96.26%)
Tropical Latin America	1751.5 (1506.2 to 2026.3)	29.68% (24.12% to 34.58%)
Southern Latin America	1524.5 (1282.6 to 1811.9)	36.05% (27.34% to 44.49%)

Supplementary Table 6 - Country-specific age-standardized DALYs rate in 2019 and percentage change 1990-2019, by type of diabetes.

Location	Type 1 diabetes		Type 2 diabetes	
	**2019 (per 100000)** Rate (95% UI)	**Change 1990-2019** % (95% UI)	**2019 (per 100000)** Rate (95% UI)	**Change 1990-2019** % (95% UI)
Canada	141.6 (108.9 to 180.3)	-0.3% (-11.8% to 13.4%)	696.9 (551 to 862.4)	20.0% (7.5% to 32.9%)
Greenland	88.9 (65.8 to 119.3)	-13.3% (-25.6% to -1.3%)	830.5 (661.5 to 1030.9)	2.7% (-15.6% to 21.4%)
USA	152.4 (122.9 to 187.1)	13.3% (1.8% to 20.5%)	1441.8 (1175.6 to 1757.4)	35.2% (29.5% to 41%)
Bolivia	193.7 (131.6 to 277.7)	-2.6% (-28.9% to 32%)	2600.4 (2112.2 to 3185.5)	19.9% (-1.2% to 46.9%)
Ecuador	160.3 (112.8 to 224.5)	36.5% (4.7% to 74.1%)	2267 (1862.2 to 2762.1)	66.4% (41.7% to 95.9%)
Peru	97.4 (70 to 131.9)	-7.1% (-29.6% to 21.7%)	1179.6 (942.9 to 1462.5)	23.3% (1.6% to 50.2%)
Antigua and Barbuda	259.3 (204.8 to 335.5)	-12.6% (-27.1% to 5.4%)	3400.7 (2872.9 to 3994.2)	13.7% (1.9% to 25.7%)
The Bahamas	263.4 (200.5 to 350.1)	-3.3% (-23.7% to 23.1%)	2770.5 (2255.6 to 3388.2)	1.9% (-11.3% to 15.8%)
Barbados	228.2 (176.8 to 297.4)	-8.2% (-25% to 13.7%)	3209.4 (2666.8 to 3818.6)	1.5% (-10.7% to 13.3%)
Belize	299 (233.5 to 386.5)	52.5% (27.3% to 81.1%)	3350.5 (2893.6 to 3859.7)	51.4% (36.1% to 68.5%)
Bermuda	109.8 (86 to 137.4)	-30.9% (-42.4% to -16.5%)	1415.2 (1136.3 to 1740.1)	-15.5% (-25.5% to -5.3%)
Cuba	81.5 (60.7 to 106.4)	-28% (-44% to -2.4%)	1555 (1177.1 to 2017.8)	-5.8% (-16.1% to 4.1%)
Dominica	310.5 (236.4 to 407.8)	4.8% (-18.2% to 34.4%)	3891 (3231.6 to 4684.2)	15.7% (0.1% to 32.8%)
Dominican Republic	204.3 (145.9 to 278.3)	34.7% (-0.5% to 78.3%)	2004.7 (1597.6 to 2464.7)	61.6% (34.2% to 93.9%)
Grenada	300.3 (233.6 to 387.4)	-9.9% (-25.6% to 9.2%)	4159.1 (3605.2 to 4837.1)	16.2% (5.4% to 26.4%)
Guyana	426.3 (308.9 to 594.2)	10.9% (-20.5% to 52%)	5413.3 (4423.9 to 6523.4)	26.3% (4.2% to 51.6%)
Haiti	362.5 (244.7 to 531.7)	-21.7% (-44.2% to 14.6%)	3791.6 (2895.5 to 4967.9)	-7.1% (-23.9% to 14%)
Jamaica	231.4 (167.5 to 322.8)	14.1% (-15.4% to 57%)	3915 (3221.5 to 4664.1)	32.5% (14.5% to 52.8%)
Puerto Rico	260.3 (194.3 to 338.4)	-5.1% (-26.8% to 21.1%)	2999.7 (2420.8 to 3669.5)	13.9% (-0.3% to 29.4%)
Saint Kitts and Nevis	274.7 (169 to 395.4)	-37.4% (-60.2% to -13.8%)	3529.9 (2927.9 to 4147.3)	-6.0% (-17.4% to 6.7%)
Saint Lucia	255 (197.4 to 328.3)	-13.8% (-29.1% to 4.5%)	3937.7 (3255.5 to 4711.9)	-9.1% (-18.9% to 0.9%)
Saint Vincent and the Grenadines	319.4 (252.6 to 405.1)	2% (-15.3% to 23.8%)	4506.7 (3885.2 to 5257.3)	10.8% (-0.7% to 22.6%)
Suriname	259.5 (193.7 to 340.5)	22.4% (-2.3% to 53.7%)	3590.5 (2979.3 to 4295.4)	46.5% (31.3% to 63.3%)
Trinidad and Tobago	262.8 (187.2 to 372.3)	-9.5% (-33.1% to 21.9%)	5256.6 (4191.2 to 6520.9)	-11.6% (-27.5% to 6.3%)
Virgin Islands	219.1 (169.6 to 283.2)	-2.8% (-24.2% to 25%)	2905.2 (2426.7 to 3478.2)	28.5% (12.4% to 45.7%)
Colombia	72.1 (52.1 to 96.9)	-29.6% (-43% to -13.3%)	1574.5 (1205.2 to 1999.9)	-6.3% (-17.5% to 4.3%)
Costa Rica	98.0 (68.3 to 139.2)	27.0% (-0.3% to 62.7%)	1688.1 (1308.2 to 2156.6)	16.2% (4.2% to 28.7%)
El Salvador	342.9 (230.7 to 507)	124.8% (64.5% to 203%)	3114.9 (2490.9 to 3813.3)	118% (80.2% to 161%)
Guatemala	323.9 (223.9 to 445.7)	106.1% (50.3% to 175.2%)	4032.9 (3298.8 to 4843.1)	167.9% (125.3% to 221%)
Honduras	210.6 (131.1 to 319.5)	43.4% (3.2% to 95.4%)	2454.6 (1972 to 3045.9)	59.4% (44.1% to 79.4%)
Mexico	283.8 (207.6 to 374.4)	60.8% (31.1% to 92.7%)	4221.7 (3630.6 to 4902.7)	13.5% (3.5% to 23.9%)
Nicaragua	286.7 (186.3 to 417.3)	57.2% (22.8% to 97%)	3501.1 (2889.4 to 4191.8)	61% (42.5% to 83%)
Panama	134.1 (94.6 to 188)	47.8% (12.7% to 87.9%)	2290.8 (1813.3 to 2831.6)	59.5% (41% to 81.4%)
Venezuela	165 (111.1 to 240.1)	42% (5% to 86.2%)	2721.5 (2193.2 to 3348.7)	31.9% (12.6% to 55%)
Brazil	131.9 (106 to 156.2)	-13.2% (-23% to -5.6%)	1762.9 (1515.7 to 2039.5)	-6.7% (-10.8% to -3%)
Paraguay	168.1 (121.1 to 233.4)	59.4% (23.3% to 110.7%)	2554.7 (2059.8 to 3104)	90.5% (58.4% to 131.1%)
Argentina	137.6 (110.2 to 166)	-8.6% (-16.2% to 0%)	1437 (1218.6 to 1697.4)	16.1% (8.5% to 23.8%)
Chile	81.0 (62.8 to 100.7)	-6.1% (-15.3% to 4.5%)	1303.6 (1051.6 to 1597.2)	24.7% (14.5% to 34.6%)
Uruguay	114.3 (89.8 to 140.3)	0.7% (-7.3% to 8.5%)	979.4 (831.1 to 1156.7)	31.7% (17.9% to 46%)

Supplementary Table 7 - Percentage of DALYs due to years of life lost (YLLs) and years lived with disability (YLDs) by type of diabetes for the Americas and by region, 1990 and 2019.

	1990		2019	
Location	YLLs % (95% UI)	YLDs % (95% UI)	YLLs % (95% UI)	YLDs % (95% UI)
**The Americas**				
**Type 1 diabetes**	**6.7% (5.4% to 8.4%)**	**2.8% (2.1% to 3.5%)**	**5.2% (4.0% to 6.7%)**	**2.2% (1.6% to 2.8%)**
**Type 2 diabetes**	**52.1% (45.4% to 59.8%)**	**38.5% (30.6% to 45.6%)**	**47.6% (40.4% to 56%)**	**45% (36.3% to 52.7%)**
HI North America				
Type 1 diabetes	6.1% (4.8% to 7.8%)	5.1% (4% to 6.5%)	4.1% (3.0% to 5.2%)	3.9% (3.0% to 5.0%)
Type 2 diabetes	43% (36.3% to 50.5%)	45.8% (37.9% to 52.8%)	36.9% (30.3% to 44.4%)	55.1% (47.0% to 62.0%)
Caribbean				
Type 1 diabetes	8.5% (6.8% to 10.2%)	0.8% (0.6% to 1.1%)	6.6% (5.0% to 8.4%)	0.9% (0.7% to 1.2%)
Type 2 diabetes	58.3% (51.6% to 65.3%)	32.4% (24.5% to 40%)	49.9% (41.0% to 58.8%)	42.6% (32.9% to 52.5%)
Central LA				
Type 1 diabetes	5.9% (4.7% to 7.6%)	0.7% (0.5% to 0.9%)	6.1% (4.5% to 8.2%)	0.6% (0.4% to 0.7%)
Type 2 diabetes	57.7% (51.2% to 65.2%)	35.7% (27.6% to 43.1%)	54.8% (47.4% to 63.4%)	38.5% (29.3% to 46.7%)
Andean LA				
Type 1 diabetes	9.4% (7.4% to 11.7%)	1.6% (1.1% to 2.2%)	6.3% (4.6% to 8.4%)	1.4% (1.0% to 1.8%)
Type 2 diabetes	61.4% (54.7% to 68.3%)	27.6% (20.3% to 35.2%)	56.9% (48% to 65.5%)	35.4% (26.2% to 45%)
Tropical LA				
Type 1 diabetes	7.3% (5.8% to 9.3%)	1.8% (1.3% to 2.4%)	4.8% (3.6% to 6.1%)	2.4% (1.8% to 3.1%)
Type 2 diabetes	56.8% (50.1% to 63.9%)	34.1% (26.5% to 41.5%)	51.6% (44.2% to 59.8%)	41.3% (33.1% to 49.1%)
Southern LA				
Type 1 diabetes	8.2% (6.3% to 10.2%)	2.3% (1.7% to 3%)	5.2% (3.9% to 6.9%)	2.3% (1.7% to 3%)
Type 2 diabetes	60.1% (53% to 67.2%)	29.5% (22.1% to 37.2%)	48.9% (40.8% to 57.5%)	43.5% (34.2% to 52.5%)

Supplementary Table 8 - All-age high fasting plasma glucose DALYs due to specific causes in 2019 by the Americas regions.

Location	Cardiovascular diseases<br>Rate (95% UI)	Neoplasms<br>Rate (95% UI)	Neurological disorders<br>Rate (95% UI)	Sense organ diseases<br>Rate (95% UI)	Tuberculosis<br>Rate (95% UI)	Type 1 diabetes<br>Rate (95% UI)	Type 2 diabetes<br>Rate (95% UI)
**The Americas**	**1469.3 (1063.6 to 2001.6)**	**294.3 (84.6 to 584.5)**	**85.9 (15.4 to 274.8)**	**11.3 (2.8 to 25.4)**	**11.8 (7.7 to 16.2)**	**179.1 (144.2 to 214.5)**	**2348.8 (1989.8 to 2765)**
HI North America	1770 (1271.7 to 2420.3)	479.9 (137 to 943.6)	114.7 (21.7 to 358)	7 (1.8 to 15.8)	1.7 (1 to 2.4)	170.6 (136.3 to 210.3)	2027.4 (1660.4 to 2460.8)
Caribbean	2132 (1508.4 to 2926.7)	259.9 (72.7 to 539.5)	74.6 (13.2 to 234)	13 (3.4 to 29.5)	32.2 (20.6 to 45.7)	234.8 (183.3 to 296.2)	3028.6 (2518.6 to 3627.2)
Central LA	1409.2 (987.6 to 1921.2)	167.9 (47.7 to 348.8)	72.2 (12.5 to 229.7)	16.3 (4.1 to 36.4)	19.8 (12.9 to 27.7)	240.5 (178.7 to 319)	3497.7 (2999.3 to 4107.5)
Andean LA	661.5 (437.1 to 960.1)	95.4 (26.1 to 205.3)	45.7 (7.7 to 149.7)	14.8 (3.6 to 34.3)	29.3 (17.8 to 43.1)	139.9 (103.3 to 185.1)	1743.5 (1459.4 to 2069.9)
Tropical LA	1163.8 (842.3 to 1630.1)	147 (40.4 to 303.1)	65.1 (11.2 to 212.7)	13.7 (3.4 to 31.1)	14.4 (9.2 to 19.9)	144.5 (115.9 to 172.1)	1972.2 (1696.4 to 2281)
Southern LA	1117.9 (784.3 to 1576.3)	281.2 (77.9 to 568.8)	68.9 (12 to 227.6)	7.9 (1.9 to 18)	9.2 (5.7 to 13.1)	133.9 (106.5 to 163.5)	1712.5 (1441 to 2036.2)

Supplementary Table 9 - Correlation of the SDI, the HAQ Index, and prevalence of diabetes with the age-standardised DALY rate due to diabetes in the Americas, 2019			
**Variable**	**Age-standardised DALYs rate**	**R^2**	**p value**
SDI (10% increase)	-411.6	0.097	0.054
HAQ Index (10% increase)	-645.4	0.36	<0.001
Prevalence of diabetes (1% increase)	322.4	0.72	<0.001

Supplementary Table 10. Diabetes prevalence and incidence rates (95% Uncertainty Intervals) in adults in the Americas in 1990 and 2019 and their change from 1990 to 2019 (95% Uncertainty Interval).

	Prevalence (age-standardised rate)			Incidence (age-standardised rate)		
**Location**	**1990 (per 100)**	**2019 (per 100)**	**% change, 1990–2019**	**1990 (per 1000)**	**2019 (per 1000)**	**% change, 1990–2019**
**Type 1 diabetes**						
**Global**	0.29 (0.24 to 0.35)	0.37 (0.30 to 0.46)	29.5 (24.6 to 34.8)	0.037 (0.027 to 0.051)	0.055 (0.039 to 0.078)	48.9 (42.3 to 55.0)
**The Americas**	0.54 (0.44 to 0.65)	0.57 (0.46 to 0.71)	6.9 (1.9 to 11.9)	0.069 (0.051 to 0.095)	0.086 (0.062 to 0.120)	24.2 (19.7 to 29.5)
High-income North America	0.86 (0.70 to 1.04)	0.96 (0.77 to 1.21)	12.0 (4.2 to 19.4)	0.120 (0.086 to 0.167)	0.163 (0.114 to 0.232)	35.8 (29.3 to 43.4)
Central LA	0.14 (0.11 to 0.17)	0.18 (0.14 to 0.22)	26.7 (24.0 to 29.4)	0.015 (0.011 to 0.022)	0.018 (0.013 to 0.027)	19.2 (14.4 to 23.4)
Caribbean	0.18 (0.15 to 0.22)	0.25 (0.20 to 0.32)	38.3 (31.3 to 46.7)	0.025 (0.018 to 0.035)	0.036 (0.026 to 0.052)	43.8 (34.4 to 52.5)
Andean LA	0.19 (0.15 to 0.24)	0.24 (0.19 to 0.31)	26.8 (23.4 to 30.6)	0.029 (0.021 to 0.042)	0.036 (0.025 to 0.050)	20.7 (16.3 to 24.5)
Tropical LA	0.32 (0.26 to 0.39)	0.46 (0.37 to 0.57)	45.9 (38.3 to 52.9)	0.047 (0.035 to 0.065)	0.077 (0.056 to 0.107)	62.6 (53.8 to 69.8)
Southern LA	0.34 (0.24 to 0.35)	0.45 (0.36 to 0.56)	35.1 (22.6 to 51.0)	0.020 (0.016 to 0.026)	0.034 (0.023 to 0.050)	68.5 (34.2 to 110.2)
**Type 2 diabetes**						
**Global**	5.6 (5.1 to 6.1)	8.3 (7.6 to 9.1)	48.9 (47.0 to 50.6)	2.9 (2.6 to 3.1)	4.0 (3.7 to 4.3)	40.5 (38.9 to 42.1)
**The Americas**	7.1 (6.6 to 7.7)	10.4 (9.6 to 11.1)	45.2 (42.5 to 48.4)	3.8 (3.5 to 4.1)	5.2 (4.8 to 5.6)	36.7 (34.7 to 39.2)
High-income North America	6.9 (6.3 to 7.5)	10.6 (9.8 to 11.4)	54.0 (48.1 to 60.8)	3.6 (3.3 to 3.9)	5.3 (4.9 to 5.7)	49.3 (44.3 to 54.9)
Central LA	10.2 (9.5 to 11.0)	13.4 (12.3 to 14.4)	30.6 (26.8 to 34.1)	5.6 (5.2 to 6.0)	6.4 (6.0 to 6.9)	15.8 (12.2 to 19.1)
Caribbean	8.3 (7.7 to 8.9)	11.8 (10.9 to 12.7)	42.3 (37.1 to 48.2)	4.3 (4.0 to 4.5)	5.6 (5.2 to 6.1)	31.5 (26.6 to 36.6)
Andean LA	4.0 (3.7 to 4.3)	6.7 (6.1 to 7.3)	68.3 (61.7 to 75.1)	2.4 (2.2 to 2.5)	3.7 (3.4 to 4.0)	55.1 (49.3 to 61.2)
Tropical LA	6.9 (6.3 to 7.4)	7.9 (7.2 to 8.6)	14.4 (11.2 to 18.1)	3.8 (3.5 to 4.1)	4.2 (3.8 to 4.5)	10.5 (7.7 to 13.7)
Southern LA	4.4 (4.1 to 4.8)	8.0 (7.2 to 8.7)	79.8 (68.5 to 91.3)	2.7 (2.5 to 2.9)	4.3 (3.9 to 4.7)	60.6 (51.5 to 70.7)

Supplementary Table 11 - Prevalent and incident cases of diabetes globally, in the Americas and by region in 2019.		
**Location**	**Prevalent cases (in thousands) N (95% UI)**	**Incident cases (in thousands) N (95% UI)**
**Type 1 diabetes**		
**Global**	**19300 (15400 to 23900)**	**281 (201 to 398)**
**The Americas**	**4200 (3400 to 5170)**	**59.4 (43.6 to 82.9)**
High-income North America	2800 (2240 to 3510)	40.4 (29.9 to 56)
Central Latin America	287 (227 to 358)	3.02 (2.05 to 4.45)
Caribbean	80 (63.6 to 100)	1.14 (0.808 to 1.6)
Andean Latin America	95.8 (75.5 to 120)	1.45 (1.02 to 2.07)
Tropical Latin America	725 (577 to 905)	11.9 (8.67 to 16.5)
Southern Latin America	214 (170 to 262)	1.56 (1.08 to 2.25)
**Type 2 diabetes**		
**Global**	**436000 (400000 to 475000)**	**21200 (19500 to 23000)**
**The Americas**	**81300 (75200 to 87200)**	**3880 (3590 to 4190)**
High-income North America	38100 (35300 to 41100)	1650 (1520 to 1790)
Central Latin America	20700 (19000 to 22200)	1040 (960 to 1120)
Caribbean	3820 (3540 to 4130)	182 (169 to 197)
Andean Latin America	2430 (2230 to 2640)	139 (128 to 151)
Tropical Latin America	12300 (11200 to 13400)	666 (612 to 729)
Southern Latin America	4070 (3670 to 4450)	208 (189 to 227)

Supplementary Table 12 - Crude population attributable fraction of DALYs due to type 2 diabetes for the main risk factors for diabetes identified by GBD 2019, by region.

Location	Air pollution % (95% UI)	Dietary risks % (95% UI)	High body-mass index % (95% UI)	Low physical activity % (95% UI)	Non-optimal temperature % (95% UI)	Tobacco % (95% UI)
**The Americas**	**10.8% (7.3% to 14.8%)**	**27.5% (23.1% to 31.6%)**	**63.2% (48.3% to 75.7%)**	**7.3% (3.7% to 12.3%)**	**3.2% (2.2% to 4.4%)**	**13.1% (9.4% to 16.3%)**
High-income North America	5% (2.3% to 8.7%)	34.5% (29.3% to 39.4%)	66.4% (51.3% to 78.1%)	5.8% (2.4% to 10.6%)	3.9% (2.7% to 5.7%)	14.6% (11.4% to 17.9%)
Central Latin America	15.5% (11.1% to 20%)	22.2% (17.9% to 26.3%)	62.1% (46% to 75.6%)	4.6% (1.8% to 8.9%)	3.4% (2.3% to 4.6%)	11.1% (7.2% to 14.7%)
Caribbean	14.4% (9.3% to 19.8%)	19.4% (14.7% to 23.5%)	56.9% (43.2% to 69.2%)	11.3% (5.9% to 18.1%)	0.5% (-0.2% to 1.3%)	12.4% (8.9% to 15.5%)
Andean Latin America	16.1% (11.4% to 20.7%)	19.4% (15% to 23.6%)	62.3% (47% to 74.9%)	4.7% (1.9% to 9%)	3.7% (2.2% to 5.4%)	7.6% (4.7% to 10.2%)
Tropical Latin America	10.8% (6.7% to 15.5%)	26.7% (22.2% to 30.9%)	63.1% (49.8% to 75.1%)	15.7% (9% to 22.8%)	1.6% (0.2% to 4.4%)	14.1% (9.9% to 17.9%)
Southern Latin America	11.6% (7.5% to 16.3%)	33.4% (28.4% to 37.8%)	55.8% (39.6% to 70.5%)	2.8% (1.2% to 5.4%)	5.4% (2.9% to 8.4%)	17% (12.3% to 20.9%)

Supplementary Figure 1: Association of the Socio-demographic Index (Panel A) and the Healthcare Access and Quality Index (Panel B) with DALYs due to type 1 diabetes

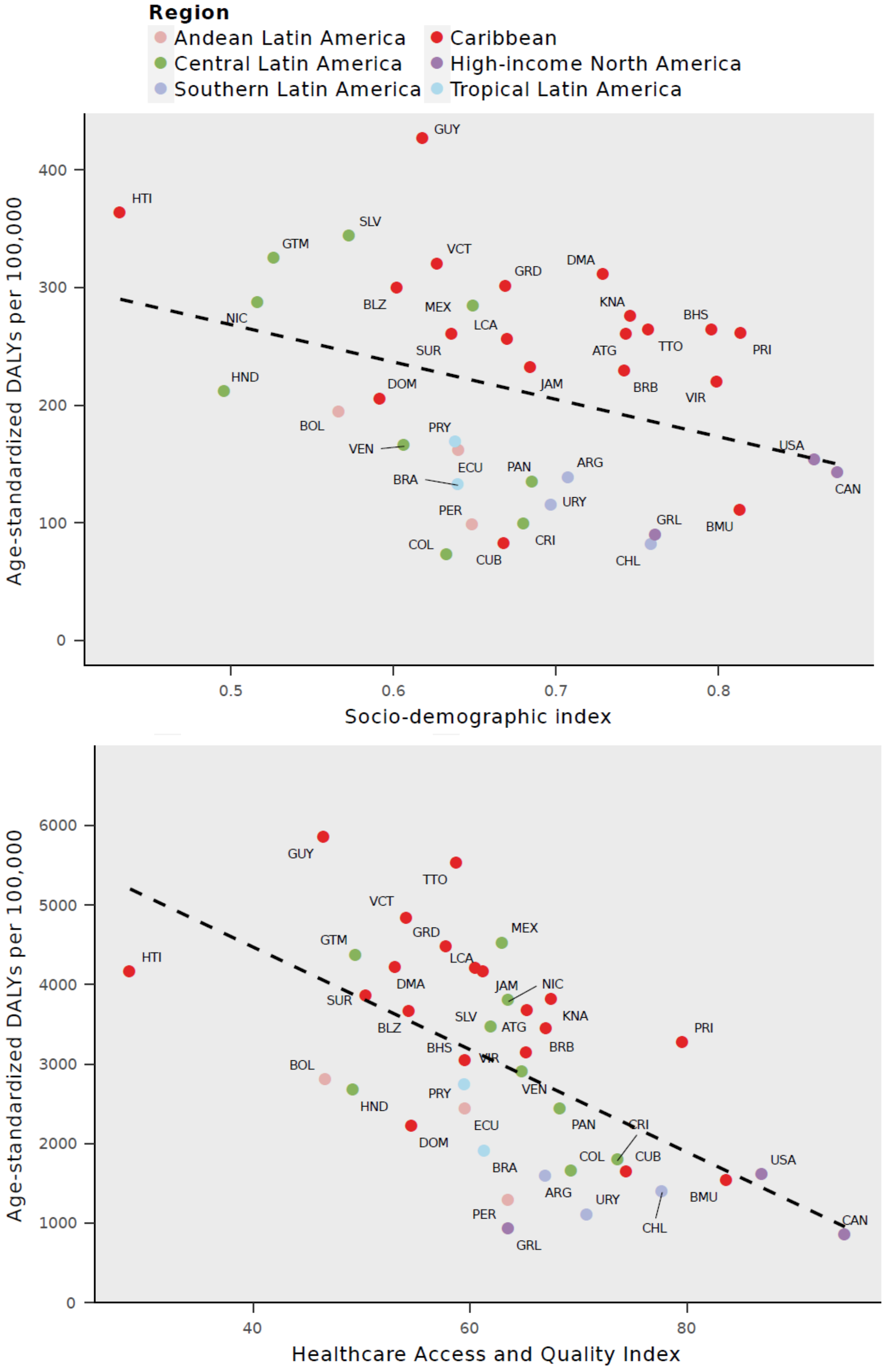

Supplementary Figure 2. Changes in the age-standardised YLLs rate and age-standardised YLDs rate in regions of the Americas over time, 1990 to 2019. Each point represents both rates for a given year for the regions shown. The years in each series, except for initial years for high-income North America, advance from left to right.

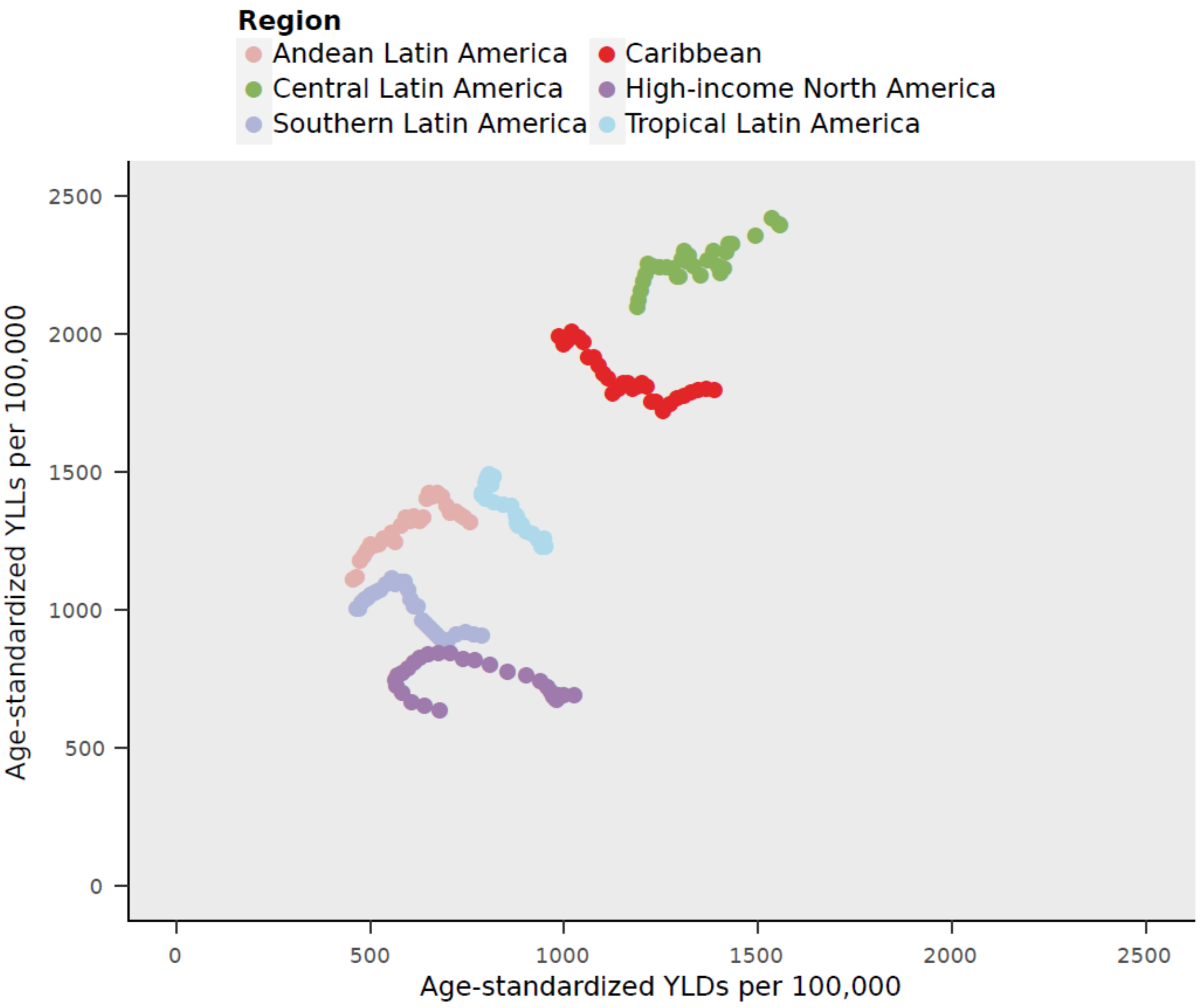

Supplementary Figure 3: Age-standardised prevalence of type 2 diabetes across the range of the prevalence of obesity in the Americas, 2019

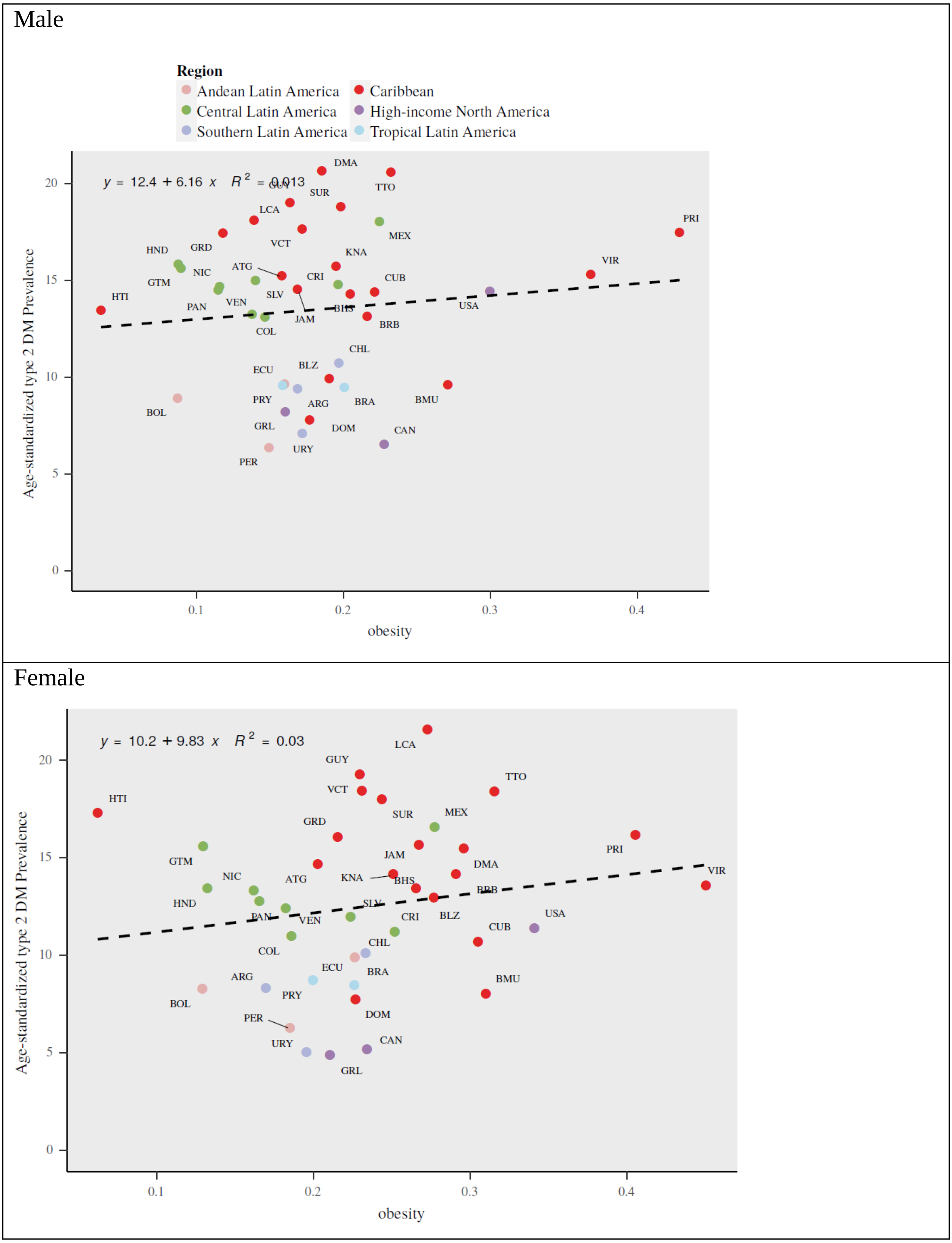

Supplement: Supplementary appendix [file mmc1.pdf]
